# Pharmacological potential of natural chalcones: a recent studies and future perspective

**DOI:** 10.3389/fphar.2025.1570385

**Published:** 2025-06-17

**Authors:** Suman Adhikari, Priyatosh Nath, Vishal Kumar Deb, Niranjan Das, Antara Banerjee, Surajit Pathak, Asim K. Duttaroy

**Affiliations:** ^1^ Department of Chemistry, Govt. Degree Collage, Dharmanagar, Tripura, India; ^2^ Department of Otolaryngology/Head and Neck Surgery, Louisiana State University Health Shreveport, Shreveport, LA, United States; ^3^ School of Health Sciences and Technology, UPES, Dehradun, Uttarakhand, India; ^4^ Department of Chemistry, Ramthakur College, Agartala, Tripura, India; ^5^ Faculty of Allied Health Sciences, Chettinad Academy of Research and Education (CARE), Chettinad Hospital and Research Institute (CHRI), Chennai, India; ^6^ Department of Nutrition, Institute of Medical Sciences, Faculty of Medicine, University of Oslo, Oslo, Norway

**Keywords:** chalcones, biosynthesis, pharmacological activity, structural features and antimicrobial activity, anti-inflammatory, anti-cancer

## Abstract

Chalcones isolated from natural sources are the primary metabolites of numerous biologically intriguing and pharmacologically essential drugs. Chalcones’ pharmacological properties are believed to result from a double bond conjugated to carbonyl functionality. This review aims to summarise the research findings, showing naturally occurring chalcones as a preferred scaffold in medicinal chemistry. Natural chalcones have an intense antimicrobial activity that targets many pathogens, including viruses, bacteria, fungi, and protozoa. Strong antibiotic qualities are exhibited by chalcones, including 4-hydroxyderricin, licochalcone A and C, isobavachalcone, and pinocembrin chalcone. Furthermore, chalcones are promising pharmacological agents for cancer treatment; they inhibit angiogenesis, decrease metastasis, and induce death in tumor cells via diverse mechanisms. Chalcones are also considered promising therapeutic agents for diabetes, neurodegenerative diseases, and cardiovascular diseases because of their anti-inflammatory and antioxidant characteristics and ability to modify enzyme functioning. This review emphasizes several aspects, such as the biosynthesis of chalcones, preparation of chalcone derivatives, isolation of chalcones, structural features of chalcones, structure-activity relationship study, the role of natural chalcones in managing various diseases and illustrates their action mechanism to control disease progression.

## 1 Introduction

**FIGURE 1:**
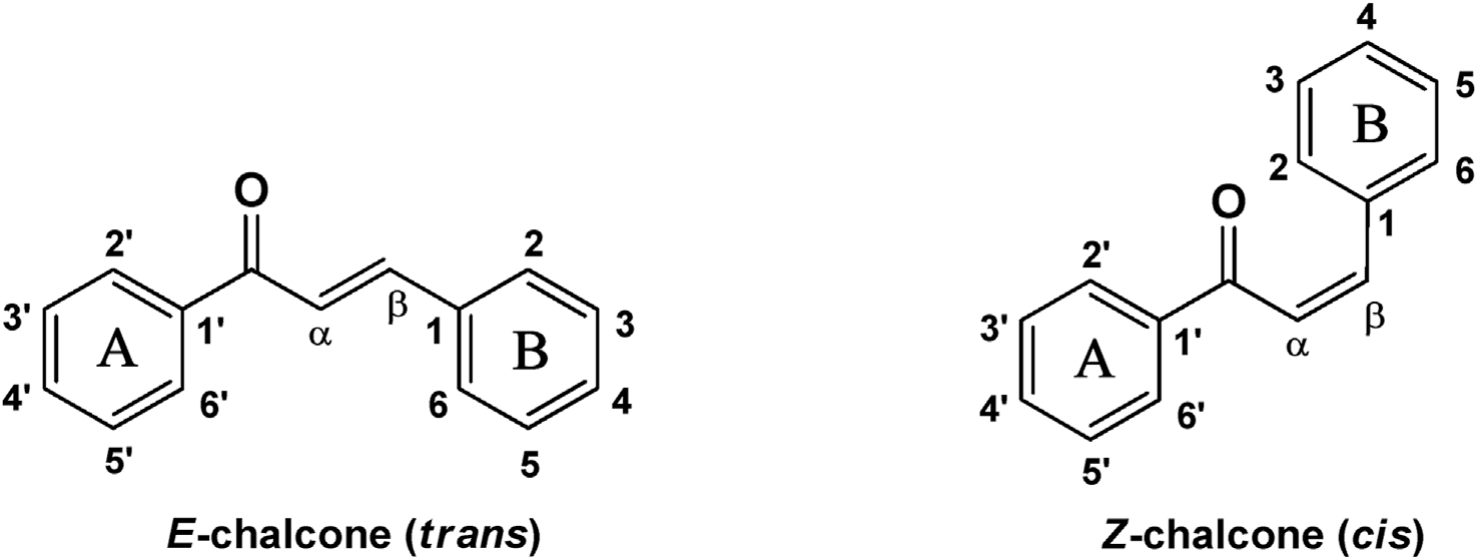
Chemical structure of chalcone (*cis* and *trans*).

**FIGURE 2:**
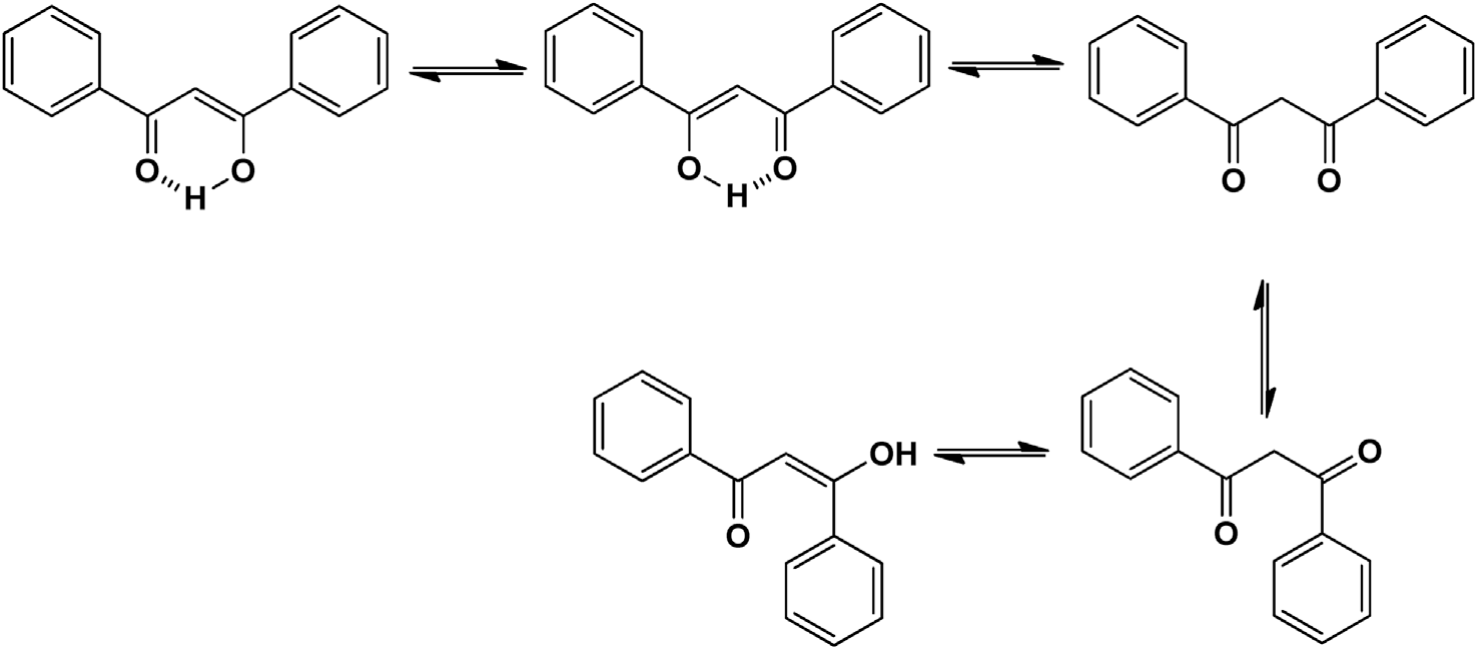
Tautomeric isomers and hydrogen bonded form of β-hydroxychalcone.

Naturally occurring metabolites have historically been the principal origin of medications for treating human disease. Plants’ therapeutic properties have been recorded in Egyptian civilizations, Chinese medicine, Indian Ayurveda, and on Assyrian clay tablets dated around 2000 B.C. Natural metabolites originate from plants, marine life, and microorganisms and continue to be crucial in developing medications for treating most human diseases. Over half of the clinical medications permitted by the US Food and Drug Administration (FDA) have been developed from natural metabolites or their corresponding synthetic analogs (Newman and Cragg, 2016). For instance, around 200 natural antibiotics derived from microbial sources have been employed as medications (Wright, 2017). Chalcones are simple pharmacological scaffolds of several naturally occurring metabolites, and plants comprising chalcones have also been utilized in traditional medicine for decades (Zhuang et al., 2017a). Chalcones belong to the open chain flavonoid family, and chalcones are chemically 1,3-diaryl-2-propen-1-ones (Figure 1), in which a three-carbon α, β-unsaturated carbonyl scaffold connects the two aromatic rings (A and B). The primed numbers are assigned to the A ring, written to the left, and the unprimed numbers are assigned to the B-ring carbons (Figure 1). Bridge carbons are marked relative to the carbonyl function. Chalcones can exist as two isomers, *trans* (*E*) and *cis* (*Z*), however the *trans* (*E*) isomer has superior thermodynamic stability since there is no steric crowding amid the carbonyl group and ring B (Figure 1). The two aromatic rings of chalcones having a π-electron system experiences delocalization with the conjugated double bonds, which results in a negligible redox potential and a better possibility of enduring electron transfer. The pharmacological properties of chalcones are assumed to be due to the existence of a double bond in conjugation with carbonyl moiety, as steric hindrance or saturation of the double bond renders the activity significantly. The aromatic rings of naturally occurring chalcones are polyhydroxylated. β-Hydroxy chalcones (also known as dibenzoylmethanes) belong to a unique type of natural metabolites, and only a few β-hydroxy chalcones (such as pongamol, pongagallone a, pongagallone b, etc.) have been isolated from plants. They are typically found as diketo-ketoenolic tautomeric mixtures with *E* and *Z* configuration (Figure 2). The *Z*-isomer kinetically controlled product, which isomerizes to the thermodynamically more stable *E*-isomer. X-ray crystallographic study also supported this isomerization (Bukhari et al., 2013). The presence of an *E* isomer was also supported by mass spectral analysis, which showed mass ions, M-OMe or M-OH, depending on the substituent at the C-6′ site of the A-ring. The existence of a downfield H-bonded -OH proton near δ 15–17 and one olefinic proton near δ 7.0–8.5 in the ^1^H-NMR spectra is the characteristic of β-hydroxy chalcones having *Z* configuration (Nielsen and Houlihan, 2011a).

**FIGURE 3:**
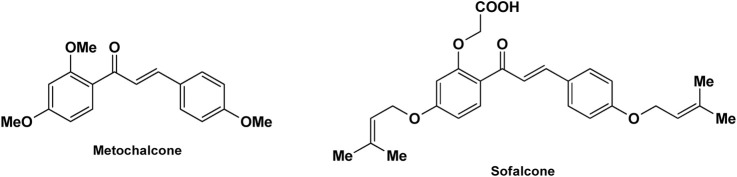
Metochalcone and sofalcone, two clinically permitted chalcone-based drugs.

Scientists have been fascinated by chalcones, the building blocks of several pharmacologically intriguing metabolites extracted from natural sources, for decades. Researchers are still fascinated by the chemistry of chalcones in the 21st century, owing to their easy preparation and several replaceable hydrogens that generate an extensive range of derivatives and intriguing biological functions (Mazumder et al., 2024; Samota et al., 2024). Chalcones are present in various foods, including fruits, teas, vegetables, and several plants, which are synthetic precursors to the biosynthesis of isoflavonoids and flavonoids (Samota et al., 2024). The most significant number of naturally occurring chalcones has been extracted from species of the Asteraceae, Leguminosae, and Moraceae families. Chalcones family has been employed to treat numerous sicknesses for thousands of years, including diabetes, inflammation, and cancer, using botanical drugs and plants (Batovska and Todorova, 2010; Karthikeyan et al., 2014; Zhou, 2015). Recently, the chalcone derivatives have sparked a lot of consideration owing to their various pharmacological attributes, including anti-tumor, anti-inflammatory, and antimicrobial properties (Singh et al., 2014; Matos et al., 2015; Mahapatra et al., 2019; Ramadan et al., 2024). Metochalcone and sofalcone are chalcone-based drugs approved for clinical use (Figure 3) (Shigeru et al., 1991; Nowakowska, 2007a; Tanaka et al., 2009; Sahun et al., 2012a). The radical quenching characteristics of several chalcones’ phenolic moieties have sparked attention to employing the chalcone-rich plant extracts as medicines or preservatives of foods (Dhar DN, 1981). Butein, a chalcone derivative having four additional hydroxy groups at the 2′, 3, 4, and 4′position, has been usually utilized in Japan, Korea, and China for treating stomach cancer, pain, parasitic infections, thrombotic disease gastritis in addition to a food additive (Kang et al., 2004; Lee et al., 2004). Isoliquiritigenin, a liquorice chalcone, treats cardiovascular disorders as a phosphodiesterase III inhibitor (Wegener and Nawrath, 1997). Xanthine oxidase (SOGAWA et al., 1994), epoxide hydrolase (Morisseau et al., 1998), aldose reductase (IWATA et al., 1999), quinone reductase (Miranda et al., 2000a), and protein tyrosine kinase (Yang et al., 2001; Nerya et al., 2004a), are just a few of the essential enzymes found in biological systems that have been reported to be inhibited by derivatives of chalcone. In addition, several other pharmacological attributes of chalcones, including anti-inflammatory, antimicrobial, cytotoxic, and anti-cancer properties, find their medicinal applications for treating different diseases (Elias DW et al., 1999; Go et al., 2005). Aside from the various therapeutic characteristics of chalcone, it has a strong skin protection effect, which is an important component in enthanopharmacological research. In this regard, long-term UV (ultraviolet light) exposure on skin cells may result in chronic damage. In this context, an *in-vitro* investigation found that dihydrochalcones such as aspalathin and nothofagin extracted from *Aspalathus linearis* (Rooibos) could protect HaCaT and SK-MEL-1 skin cells. As a result, it was determined that these chalcones pre-treatment may be associated with greater cellular adaptability by decreasing lipid peroxidation and caspase 3 expression, potentially reducing UVB-mediated oxidative stress in human skin cells (Akinfenwa et al., 2021). Furthermore, an *in-vivo* model-based study found that hesperidin methyl chalcone (HMC) inhibits UVB-induced inflammation and oxidative stress. In this context, exposing hairless mice to a UVB irradiation level of 4.14 J/cm^2^ resulted in oxidative stress and skin inflammation. After treating the HMC, it was investigated that superoxide anion formation from UVB irradiation is reduced with a lower quantity of lipid hydroperoxides (Martinez et al., 2015). In addition to disease protection, isolated chalcones from plants may work as a skin protector. Numerous reviews have been published on synthetic and natural chalcones (Nasir Abbas Bukhari et al., 2012; Kamal et al., 2013; Sharma et al., 2013; Leon-Gonzalez et al., 2015; Mahapatra et al., 2015b; Das and Manna, 2016). This review will focus on recent breakthroughs in medicinal chemistry that have used naturally occurring chalcone as a privileged pharmacological scaffold and aims to initiate more pharmacological and medicinal research into the realm of chalcone chemistry.

The scope of the present review is wide-ranging and comprehends a multidisciplinary investigation of naturally occurring chalcones in the context of their clinical potential against various diseases. Focusing primarily on research articles published in the last 25 years, this review article attempts to showcase the most current developments using therapeutic potentials of naturally occurring chalcones in medicinal chemistry. The review presents the comprehensive features of naturally occurring chalcones, including their biosynthesis, synthetic approaches, antimicrobial, anti-cancer, antioxidant, anti-inflammatory, enzyme actions, antiobesity, cardioprotective activity, antidiabetic, and neuroprotective activity. In addition, this review also emphasizes the structural features of chalcones, structure-activity relationship (SAR: defines the relationship between the chemical structure and biological activity) studies, mechanism of actions, and marketed and clinically approved chalcones. The insights provided here aim to guide future research in exploring naturally occurring chalcones with enhanced pharmacological effectiveness against various diseases. Furthermore, this scientific literature review has been designed with essential studies on chalcone across 31-year (1993–2024) based on chemical structure, molecular mechanisms, and its application to various diseases as a therapeutic potential. A complete literature search was conducted using databases such as PubMed, Research Gate, ScienceDirect, and Springer Link to discuss the details and insights.

## 2 Biosynthesis of chalcone

**FIGURE 4:**
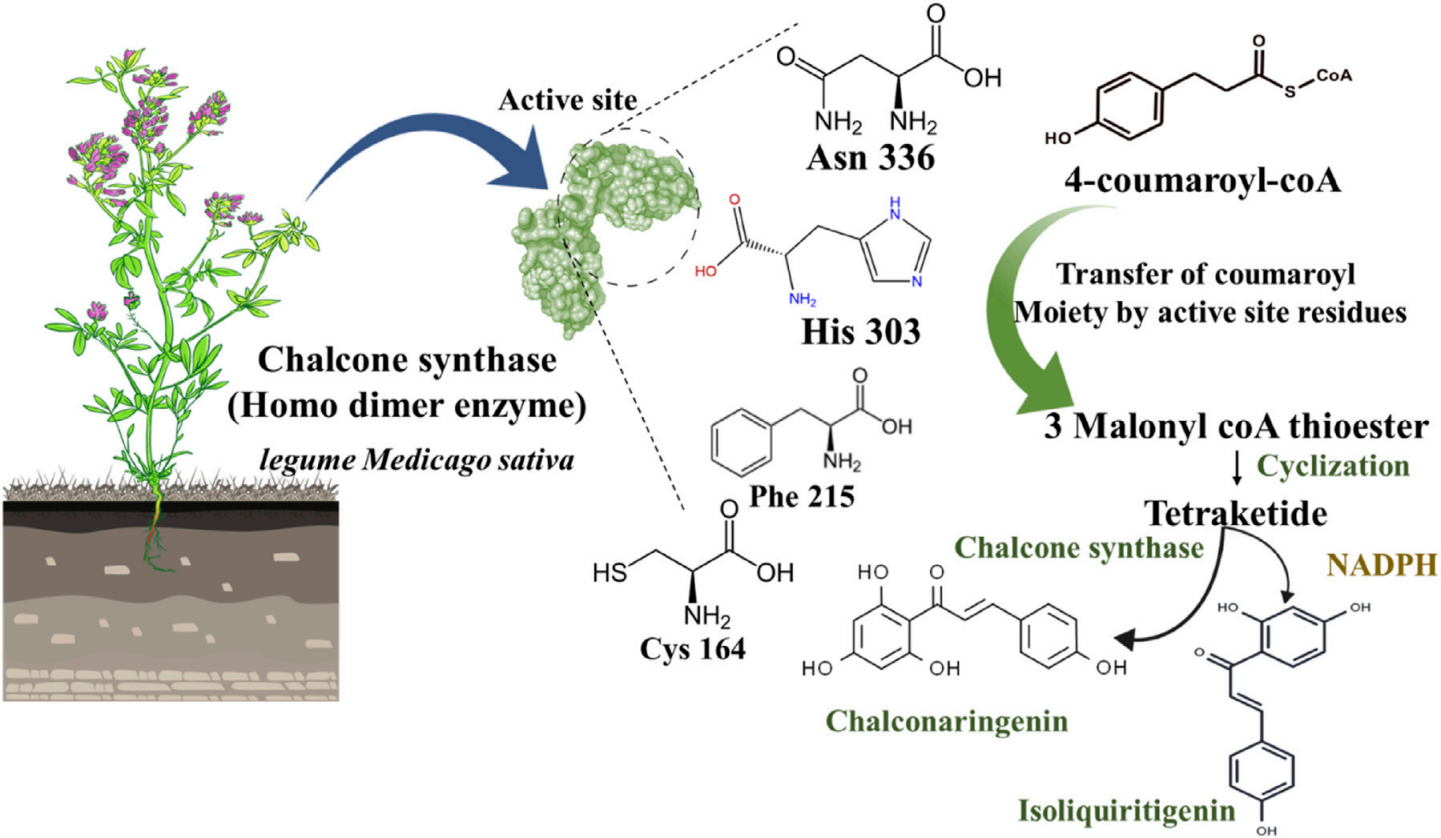
Schematic representation of chalcone biosynthesis in chalcone synthase and NADPH presence.

Noel P Joseph et al. described the mechanism of the chalcone biosynthesis process by chalcone synthase in *legume Medicago sativa* plant (Jez and Noel, 2000). Chalcone synthase is a polyketide synthase type III enzyme found in all higher plants. This enzyme is also found in lower plants like liverwort *Marchantia polymorpha*. Structurally, it is a homodimer where a single monomer has 42–45 kDa molecular weight. Notably, some amino acid residues are also identified as situated in the active site of this enzyme, including Cys164, Phe215, His303, and Asn336 (Figure 4). In the biosynthetic mechanism, chalcone synthase transfers the coumaroyl scaffold from one 4-coumaroyl-coenzyme A (CoA) to its active site residue Cys164. Subsequently, the polyketide reaction occurs where an intermediate product forms as three malonyl-CoA thioesters. After this thioester-linked tetraketide formation, a cyclization reaction occurs generating a naringenin chalcone.

Further, this naringenin chalcone converts into the 6′-deoxy naringenin chalcone through chalcone reductase and chalcone synthase (Zhuang et al., 2017b). To delve into the depth of this biosynthetic pathway, a phenylpropanoid CoA (4-coumaroyl CoA) endures in a condensation reaction with three malonyl-CoA to form a tetraketide precursor which further goes into a cyclization reaction through a different pathway (Figure 4). Out of two distinct pathways, the primary path undergoes a cyclization process through chalcone synthase only to generate chalconaringenin. In the second pathway, the presence of nicotinamide adenine dinucleotide phosphate (NADPH: a co-enzyme that donates the hydrogens and electrons in anabolic metabolism) aids in the reduction reaction of tetraketide, and then it undergoes cyclization by chalcone synthase to form a 6′-deoxy chalcone (Figure 4) (Rammohan et al., 2020). Simultaneously, other molecules like phloroglucinols, benzophenones, and stilbenes are also synthesized as secondary metabolites. In this biosynthetic pathway, naringenin chalcone as a substrate produces flavonoids and isoflavonoids by chalcone synthase and chalcone isomerase (Zhuang et al., 2017b).

## 3 Various synthetic methods for the preparation of chalcones

Chalcones are considered a privileged scaffold and are typically employed in several pharmacological activities associated with drug discovery. As a result, researchers have kept looking for new advanced techniques and low-cost procedures for synthesizing chalcones and their derivatives. Chalcones are often synthesized by base or acid-catalyzed condensation processes. Conventional Claisen-Schmidt condensation is another method for the preparation of chalcone derivatives attributable to get higher yields than other procedures (Rammohan et al., 2020). The Suzuki coupling, Heck coupling, Wittig reaction, Friedel-Crafts acylation with cinnamonoyl chloride, Photo-Fries rearrangement of phenyl cinnamates, etc., are some well-known methods for the preparation of chalcone derivatives (Bukhari et al., 2013).

### 3.1 Claisen-schmidt reaction

The preparation of chalcone derivatives by the Claisen-Schmidt reaction comprises the condensation of derivatives of acetophenone and aldehyde in polar solvents in the presence of catalysts (acid or base). Usually, aq. NaOH or KOH or ethanolic NaOEt or potassium *tert*-butoxide is used to carry out the base-catalyzed Claisen-Schmidt reaction (Table 1) (Rammohan et al., 2020). The hydroxyl-substituted chalcone synthesis is commonly carried out using the base-catalyzed Claisen-Schmidt reaction, which usually provides good to outstanding yields. In the base-catalyzed Claisen-Schmidt reaction, the chalcone is formed from the aldol via the dehydration of enolate. In contrast, in an acid-catalyzed reaction, the chalcone is formed through an enol mechanism (Nielsen and Houlihan, 2011b).

**TABLE 1:** Reaction scheme for synthesizing chalcone with different solvent systems.

Name of the reaction	Scheme	Reaction conditions	Solvent	Reference
Claisen-Schmidt Condensation	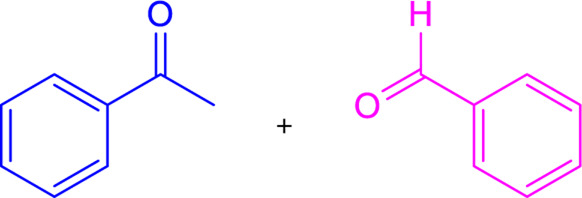	Base catalysed (KOH, KOH, Ba(OH)_2_, Ca(OH)_2_, Sr(OH)_2_, CaO, NaH, LiHMDS, LiOH etc.) Acid catalysed (AlCl_3_, HCl, BF_3-_Et_2_O, SOCl_2_, *p*-TsOH etc.)	Ethanol, methanol, THF Ethanol, methanol, dioxane, acetic acid, carbon disulphide	Gaonkar and Vignesh (2017), Zhuang et al. (2017b)
Suzuki Coupling	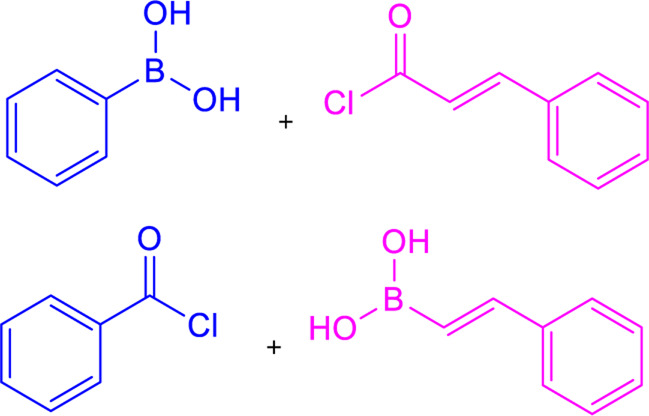	Catalyst: 3% PdCl_2_, base: Na_2_CO_3_ Catalyst: tetrakis(triphenylphosphine)palladium(0), base: CeCO_3_	Acetone/water = 3/1 Anhydrous toluene	Bumagin and Korolev (1999), Haddach and McCarthy (1999), Eddarir et al. (2003)
Heck coupling	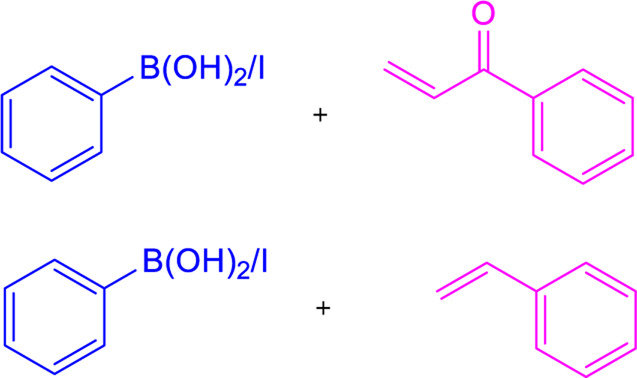	Palladium catalyst Palladium catalyst, CO	DMF, CH_3_CN Toluene	Hird et al. (1993), Brennführer et al. (2009)
Wittig Reaction	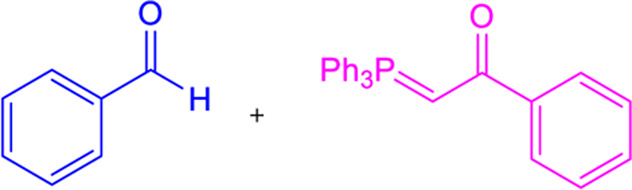		Benzene, THF	Ramirez and Dershowitz (1957)
Sonogashira isomerization coupling	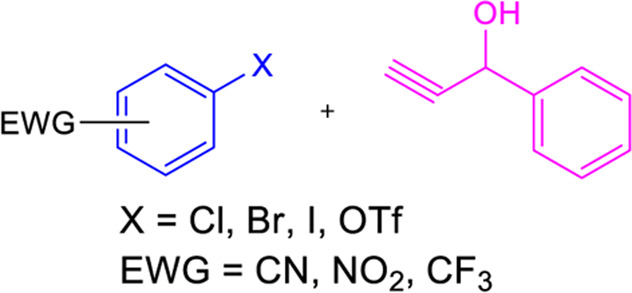	PdCl_2_(PPh_3_)_2_, CuI	THF	Rammohan et al. (2020)
Julia-Kocienski Olefination	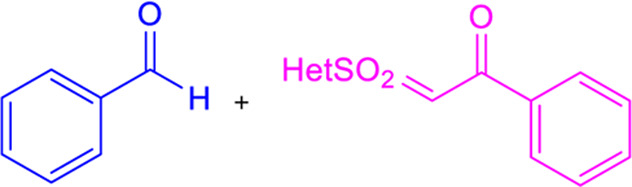	1,8-Diazabicyclo[5.4.0]undec-7-ene (DBU), LiHMDS	DCM, THF, CHCl_3_, CH_3_CN	Kumar et al. (2010)
Friedel–Crafts Acylation with Cinnamoyl Chloride	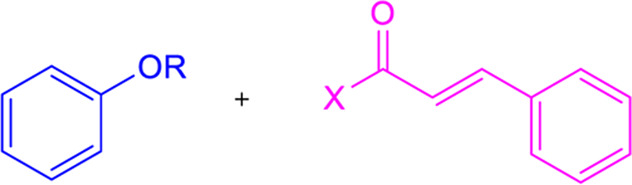	AlCl_3_		Shotter et al. (1978)
Photo-Fries Rearrangement of Phenyl Cinnamates	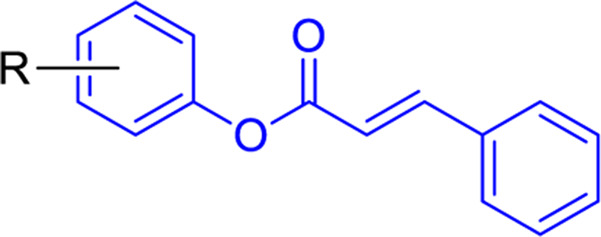	h^٧^, Inert atmosphere (N_2_)	Benzene, CHCl_3_	Obara et al. (1969), Ramakrishnan and Kagan (1970)

### 3.2 Suzuki coupling

Two possible methods for synthesizing chalcone derivatives by Suzuki coupling are combining benzoyl chloride with phenylvinylboronic acid or cinnamoyl chloride with phenylboronic acid (Eddarir et al., 2003). The conditions of the Suzuki coupling reaction have an impact on the yield. For instance, coupling cinnamoyl chloride with phenylboronic acids under these conditions (acetone/water = 3/1; 3% PdCl_2_; Na_2_CO_3_) results in a moderate yield (23%–37%), whereas isolated yields of ∼50 and ∼90% are obtained under these conditions (anhydrous toluene; tetrakis (triphenylphosphine) palladium (0); CeCO_3_) (Table 1) (Haddach and McCarthy, 1999). Chalcones having electron-withdrawing or electron-donating moieties can also be synthesized via an extended Suzuki coupling procedure.

### 3.3 Heck coupling

Heck coupling provides an efficient way to synthesize chalcones by combining aryl vinyl ketones and aryl boronic acids over the formation of carbon-carbon bonds (Table 1) (Rammohan et al., 2020). Under catalytic conditions [Pd (OAc)_2_, Ph_3_P, K_2_CO_3_, DMF], aryl vinyl ketones are combined with ArI or aryl boronic acids to generate chalcones in good yields (Hird et al., 1993; Bumagin and Korolev, 1999). Chalcones have also been prepared by carbonylative Heck coupling using palladium catalysts and the carbonylative vinylation of aryl halides with styrene in carbon monoxide. While the metal-catalyzed Heck reaction is considered an extremely effective method for synthesizing chalcones, its use is restricted due to the scarcity of aryl vinyl ketones and the requirement for pressurized CO (Wu et al., 2010).

### 3.4 Wittig reaction

Chalcones can be synthesized via the Witting olefination reaction. The reaction between triphenylbenzoylmethylene phosphorane and benzaldehyde in tetrahydrofuran (THF) produced chalcones with 70% yield (Table 1) (RAMIREZ and DERSHOWITZ, 1957). Furthermore, a microwave-assisted synthesis of chalcones with a fast reaction time (5-6 min) and excellent yields was discovered. To obtain high yields, this creative endeavor enhances the reaction rates of the Wittig olefination reaction while decreasing the reaction time (Xu et al., 1995).

### 3.5 Sonogashira isomerization coupling

In the Sonogashira isomerization coupling reaction, the chalcone derivatives are prepared by treating ArX and aryl or alkenyl 1-propargyl alcohols in equimolar amounts catalyzed by PdCl_2_(PPh_3_)_2_ in THF (Table 1) (Rammohan et al., 2020).

### 3.6 Julia–Kocienski olefination

Julia–Kocienski olefination produces *E*-chalcones as the major product even at low temperatures. It involves directly coupling heteroaryl sulfonyl phenylethanone and aromatic aldehydes under basic conditions (Table 1) (Kumar et al., 2010).

### 3.7 Friedel-Crafts acylation with cinnamoyl chloride

By Friedel-Crafts acylation of an aromatic ether and cinnamoyl chloride, chalcone derivatives can be synthesized in the presence of a Lewis acid catalyst (AlCl_3_) (Table 1) (Shotter et al., 1978). Although this process was utilized to prepare highly substituted chalcones, it is a less popular procedure for the synthesis of chalcones.

### 3.8 Photo-fries rearrangement of phenyl cinnamates

Photo-Fries rearrangement was used to prepare 2-hydroxy substituted chalcones from phenyl-cinnamate under an inert atmosphere (N_2_) using benzene as a solvent (Table 1) (Obara et al., 1969). Alcohols and chloroform solvents can also perform the photo-fries rearrangement reaction of chalcones, increasing yields by up to 50% (Ramakrishnan and Kagan, 1970). This process is not commonly used because of its limitations, such as longer reaction time, poor yield, etc.

## 4 Role of naturally occurring chalcones in different pharmacological activities

Since natural metabolites have been revealed to have positive outcomes on an inclusive range of common and general diseases, such as cancer, cardiovascular disease, parasitic illnesses, type 2 diabetes mellitus, infectious diseases, and illnesses of the central nervous system, interest in and attraction toward naturally occurring metabolites have been steadily growing (Das et al., 2023c; Debnath et al., 2024; Sinha et al., 2024; Maity et al., 2025). These naturally occurring metabolites result from millions of centuries of evolution and natural selection, display efficacy and selectivity in interaction with biomolecular targets, and can efficiently avoid current antibiotic resistance. The chalcone-rich botanical drugs and plants were employed in traditional medicinal practice for eras. Naturally occurring chalcones were extracted for the first time in 1910 and attracted a lot of consideration because of their significant pharmacological attributes (Shimokoriyama M, 1962). Many chalcones also got formal medical approval for clinical trials against cancer, viral infections, and cardiovascular disorders (Salehi et al., 2021). In medicinal chemistry, chalcones are regarded as prime compounds for developing novel therapeutics (Zhuang et al., 2017b).

### 4.1 Antimicrobial activity of natural chalcones

Roughly 7.7 million of the approximately 13.7 million fatalities caused by infectious disease in 2019 were interrelated to 33 prevalent pathogens. *Pseudomonas aeruginosa, Streptococcus pneumoniae, Klebsiella pneumoniae, Staphylococcus aureus*, and *Escherichia coli* are responsible for 54.9% of these deaths, and the majority of deaths globally are caused by *S. aureus* infections (Adhikari et al., 2020; Ikuta KS et al., 2022; El-Helw et al., 2024). Furthermore, in medicinal chemistry, new advances in potential bioactive chalcone hybrids have been explored to play a vital role as antibacterial agents (Maurya and Agrawal, 2024). It has been found that chalcones are effective towards numerous gram-positive and negative bacteria, fungi, protozoa, and even viruses. Chalcone compounds like pinocembrin chalcone, 4^′^,6′-dihydroxy-3′,5′-dimethyl-2′-methoxychalcone, licochalcone A and C, isobavachalcone, 4-hydroxyderricin, xanthoangelol, xanthoangelol F, bavachalcone, broussochalcone B, panduratin A etc. are potent antibiotic in nature. They were found to exhibit their activity against numerous microbes, including *staphylococcus, bacillus, mycobacterium, legionella, micrococcus, enterococcus* and *streptococcus,* etc. (Bremner and Meyer, 1998; Sugamoto et al., 2011). Meyer and Bremner isolated, characterized and evaluated the antibacterial activity of pinocembrin chalcone and its isomer 5,7-dihydroxy flavanone. The pinocembrin chalcone was extracted from *Helichrysum trilineatum* and characterised by mass and NMR spectra (^1^H and ^13^C NMR). Antibacterial tests revealed that pinocembrin chalcone (1.0 μg) was effective in preventing *S. aureus* from growing but inactive against *Candida* species (Table 2) (Bremner and Meyer, 1998). The antibacterial property of pinocembrin chalcone might be aided by the presence of three phenolic -OH group and an α,β unsaturated ketone structure.

**TABLE 2:** Plant source, doses, and antimicrobial roles of different natural chalcones.

Name and structure of chalcone	Source	Smiles	Activity	Concentration and duration of treatment	Target organism	References
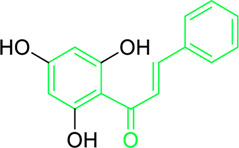 Pinocembrin chalcone	*Helichrysum trilineatum* DC (Family: *Asteraceae*)	OC1=CC(O)=CC(O)=C1C(/C=C/C2=CC=CC=C2)=O	Antibacterial.	1.0 μg	*Staphylococcus aureus*	Bremner and Meyer (1998)
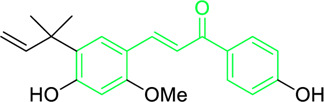 Licochalcone A 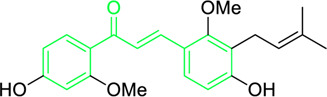 Licochalcone C	*Glycyrrhiza inflata* (Family*: Fabaceae*)	OC1=C(C(C)(C)C=C)C=C(/C=C/C(C2=CC=C(O)C=C2)=O)C(OC)=C1 OC1=CC=C(C(/C=C/C2=CC=C(O)C(C/C=C(C)\C)=C2OC)=O)C(OC)=C1	Antibacterial. Cause complete inhibition of the outgrowth of *B. subtilis* spores.	2-3 μg/mL.	*Bacillus subtilis*	Tsukiyama et al. (2002)
Licochalcone A	*Glycyrrhiza uralensis* Fisch. ex DC. (Family*: Fabaceae*)	OC1=C(C(C)(C)C=C)C=C(/C=C/C(C2=CC=C(O)C=C2)=O)C(OC)=C1	Antibacterial. Exerts antibacterial activity against human pathogenic *Mycobacteria* and *Legionella* species.	1-4 mg/L.	*Mycobacterium tuberculosis, Mycobacterium bovis; Legionella species (L. pneumophila, L. longbeacheae, L. bozemanii, L. wadsworthi, L. dumoffii, and L. feelei.*	Friis-Møller et al. (2002)
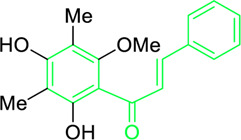 4′,6′-Dihydroxy-3′,5′-dimethyl-2′-methoxychalcone	*Dalea versicolor* Zucc. (Family: *Fabaceae*)	OC1=C(C)C(O)=C(C(/C=C/C2=CC=CC=C2)=O)C(OC)=C1C	Antibacterial.	30 μg/mL.	*Staphylococcus aureus, Bacillus cereus.*	Belofsky et al. (2004)
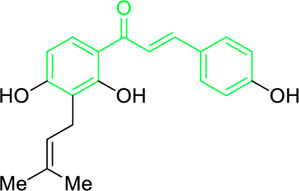 Isobavachalcone	*Angelica keiskei* (Miq.) Koidz. (Family: *Apiaceae*)	OC1=CC=C(C(/C=C/C2=CC=C(O)C=C2)=O)C(O)=C1C/C=C(C)\C	Antibacterial	MIC 4 µg/mL.	*Bacillus sublitis, Staphylococcus epidermidis, Micrococcus luteus.*	Sugamoto et al. (2011)
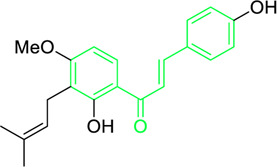 4-hydroxyderricin	*Angelica keiskei* (Miq.) Koidz. (Family: *Apiaceae*)	OC1=C(C(/C=C/C2=CC=C(O)C=C2)=O)C=CC(OC)=C1C/C=C(C)/C	Antibacterial	MIC 2 µg/mL.	*Bacillus sublitis, Staphylococcus epidermidis, Micrococcus luteus.*	Sugamoto et al. (2011)
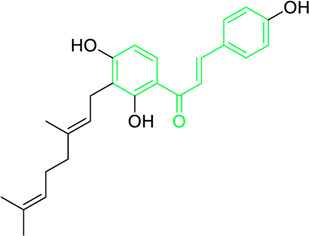 Xanthoangelol	*Angelica keiskei* (Miq.) Koidz. (Family: *Apiaceae*)	OC1=C(C/C=C(C)/CC/C=C(C)/C)C(O)=C(C(/C=C/C2=CC=C(O)C=C2)=O)C=C1	Antibacterial	MIC 4 µg/mL.	*Bacillus sublitis, Staphylococcus epidermidis, Micrococcus luteus.*	Sugamoto et al. (2011)
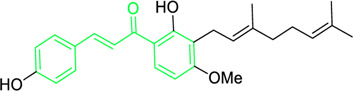 Xanthoangelol F	*Angelica keiskei* (Miq.) Koidz. (Family: *Apiaceae*)	C/C(CC/C=C(C)/C)=C\CC1=C(OC)C=CC(C(/C=C/C2=CC=C(O)C=C2)=O)=C1O	Antibacterial	MIC 64 µg/mL.	*Bacillus sublitis, Staphylococcus epidermidis, Micrococcus luteus.*	Sugamoto et al. (2011)
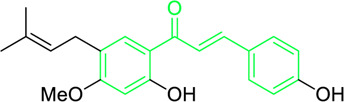 Bavachalcone	*Angelica keiskei* (Miq.) Koidz. (Family: *Apiaceae*)	OC1=CC(OC)=C(C/C=C(C)/C)C=C1C(/C=C/C2=CC=C(O)C=C2)=O		MIC 4 µg/mL.	*Bacillus sublitis, Staphylococcus epidermidis, Micrococcus luteus.*	Sugamoto et al. (2011)
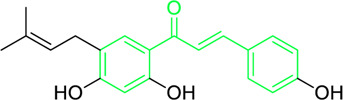 Broussochalcone B	*Angelica keiskei* (Miq.) Koidz. (Family: *Apiaceae*)	OC1=C(C/C=C(C)/C)C=C(C(/C=C/C2=CC=C(O)C=C2)=O)C(O)=C1		MIC 8-16 µg/mL.	*Bacillus sublitis, Staphylococcus epidermidis, Micrococcus luteus.*	Sugamoto et al. (2011)
Licochalcone A	*Glycyrrhiza uralensis* Fisch. ex DC. (Family*: Fabaceae*)	OC1=C(C(C)(C)C=C)C=C(/C=C/C(C2=CC=C(O)C=C2)=O)C(OC)=C1	Inhibits growth of protozoan. Reduced the rate of infection in macrophages -derived from human peripheral blood monocyte and U937 cells.	5 μg/mL.	*Leishmania donovani,* and *promastigotes.*	Chen et al. (1993)
Licochalcone A	*Glycyrrhiza uralensis* Fisch. ex DC. (Family*: Fabaceae*)	OC1=C(C(C)(C)C=C)C=C(/C=C/C(C2=CC=C(O)C=C2)=O)C(OC)=C1	Antiprotozoal. Diminished the development of chloroquine-resistant (Dd2) and chloroquine-susceptible (3D7) *Plasmodium falciparum* strains.	0.1-0.5 μg/mL (*in vitro.*) 10-15 mg/kg (*in vivo.*)	*Chloroquine-susceptible (3D7) and chloroquine-resistant (Dd2) Plasmodium strains* *P. falciparum,* *and P. yoelii.*	Chen et al. (1994)
Licochalcone A	*Glycyrrhiza uralensis* Fisch. ex DC. (Family*: Fabaceae*)	OC1=C(C(C)(C)C=C)C=C(/C=C/C(C2=CC=C(O)C=C2)=O)C(OC)=C1	Antiprotozoal. Licochalcone A caused ultrastructural alteration in a dose-dependent manner without causing harm of macrophages.	1 mg/mL.	*Leishmania sp.*	Zhai et al. (1995)
Licochalcone A	*Glycyrrhiza uralensis* Fisch. ex DC. (Family*: Fabaceae*)	OC1=C(C(C)(C)C=C)C=C(/C=C/C(C2=CC=C(O)C=C2)=O)C(OC)=C1	Antiprotozoal. It repressed the bc1 complex and complex II of *Plasmodium falciparum* mitochondria.	IC_50_ value for SQR activity inhibition is reported 1.30 µM. IC_50_ value for bc1 complex and DHOD inhibition is found between 0.077 to 0.10 µM.	*Plasmodium falciparum.*	MI-ICHI et al. (2005)
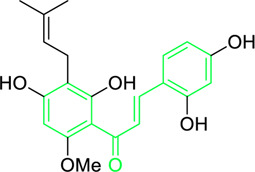 Xanthohumol	Hop extract (Species not mentioned) (Family: *Cannabaceae*)	O=C(/C=C/C1=C(O)C=C(O)C=C1)C2=C(OC)C=C(O)C(C/C=C(C)\C)=C2O	Antiviral	BVDV (TI = 6.0), HSV-1 (TI = >1.9), HSV-2 (TI = >5.3)	Bovine viral diarrhoea virus, Herpes simplex virus-1, and Herpes simplex virus-2	Buckwold et al. (2004)
Xanthohumol	*Humulus lupulus* L. (Family: *Cannabaceae*)	O=C(/C=C/C1=C(O)C=C(O)C=C1)C2=C(OC)C=C(O)C(C/C=C(C)\C)=C2O	Antiviral	EC_50_ = 20.74 µg/mL	Human immunodeficiency viruses-1	Wang et al. (2004)
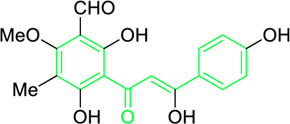 2-Methoxy-3-methyl-4,6-dihydroxy-5-(3′-hydroxy)cinnamoylbenzaldehyde	*Desmos* spp. [*Desmos chinensis* Lour. (Family: *Annonaceae*); *Desmos grandifolius* (Finet & Gagnep.) C.Y.Wu ex P.T.Li (Family: *Annonaceae*); *Desmos dumosus* (Roxb.) Saff. (Family: *Annonaceae*); and *Desmos yunnanensis* (Hu) P.T.Li (Family: *Annonaceae*)]	O=C(/C=C(O)/C1=CC=C(O)C=C1)C2=C(O)C(C)=C(OC)C(C=O)=C2O	Antiviral	EC_50_ = 0.022 µg/mL	Human immunodeficiency viruses	Wu et al. (2003)
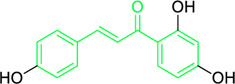 Isoliquiritigenin	*Glycyrrhiza uralensis* Fisch. ex DC. (Family*: Fabaceae*)	OC(C=C1O)=CC=C1C(/C=C/C2=CC=C(O)C=C2)=O	SAR based inhibition of neuraminidase activity.	IC_50_ = 9.0 µM	Influenza virus	Ryu et al. (2010b)
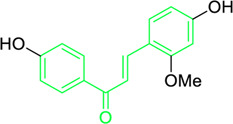 Echinantin	*Glycyrrhiza inflata* Batalin (Family*: Fabaceae*)	O=C(/C=C/C1=C(OC)C=C(O)C=C1)C2=CC=C(O)C=C2	Antiviral	IC_50_ = 2.49 ± 0.14 µg/mL	H1N1 influenza, H274Y mutant form of H1N1.	Dao et al. (2011)
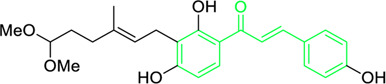 Xanthokeistal A	*Angelica keiskei* (Miq.) Koidz. (Family: *Apiaceae*)	O=C(/C=C/C1=CC=C(O)C=C1)C2=C(O)C(C/C=C(C)/CCC(OC)OC)=C(O)C=C2	Antiviral, causes inhibition of neuraminidase activity	IC_50_=12.3 μM	Influenza virus	Park et al. (2011)
Licochalcone A	*Glycyrrhiza inflata* Batalin (Family*: Fabaceae*) *Glycyrrhiza glabra* L. (Family*: Fabaceae*)	OC1=C(C(C)(C)C=C)C=C(/C=C/C(C2=CC=C(O)C=C2)=O)C(OC)=C1	Antibacterial	25–250 µg/mL (*G. inflata)* 12.5–25µg/mL (*G. glabra)*	*Streptococcus mutans*, *Lactobacillus buchneri*, and *Staphylococcus aureus*.	van Dinteren et al. (2022)
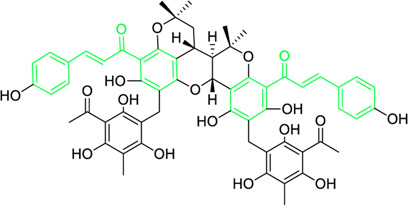 Kamalachalcone E 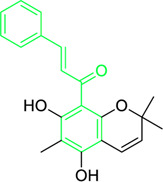 1-(5,7-dihydroxy-2,2,6-trimethyl-2H-1-benzo-pyran-8-yl)-3-phenyl-2-propen-1-one 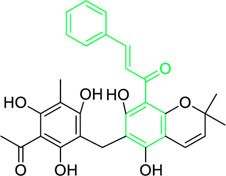 Rottlerin 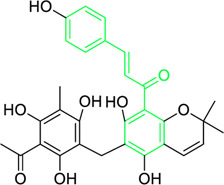 4′-hydroxyrottlerin.	*Mallotus philippinensis* (Lam.) Muell.Arg. (Family: *Euphorbiaceae*)	OC(C=C1)=CC=C1/C=C/C(C2=C(OC(C)(C)C[C@@]34[H])C3=C(O[C@]5([H])[C@]4([H])C(C)(C)OC6=C5C(O)=C(CC7=C(O)C(C)=C(O)C(C(C)=O)=C7O)C(O)=C6C(/C=C/C8=CC=C(O)C=C8)=O)C(CC9=C(O)C(C(C)=O)=C(O)C(C)=C9O)=C2O)=O O=C(C1=C(O)C(C)=C(O)C2=C1OC(C)(C)C=C2)/C=C/C3=CC=CC=C3 O=C(C1=C(O)C(CC2=C(O)C(C)=C(O)C(C(C)=O)=C2O)=C(O)C3=C1OC(C)(C)C=C3)/C=C/C4=CC=CC=C4 O=C(C1=C(O)C(CC2=C(O)C(C)=C(O)C(C(C)=O)=C2O)=C(O)C3=C1OC(C)(C)C=C3)/C=C/C4=CC=C(O)C=C4	Antifungal	8, 4 and 16 µg/mL	*Cryptococcus neoformans, and Aspergillus fumigatus*	Kulkarni et al. (2014)
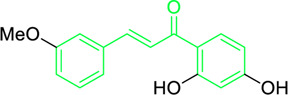 2ʹ,4ʹ-dihydroxy-3ʹ-methoxychalcone, 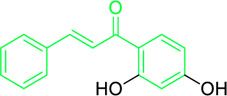 2ʹ,4ʹ-dihydroxychalcone	*Zuccagnia punctata* Cav. (Family: *Caesalpiniaceae*)	OC(C=C1)=CC(O)=C1C(/C=C/C2=CC(OC)=CC=C2)=O OC(C=C1)=CC(O)=C1C(/C=C/C2=CC=CC=C2)=O	Antifungal	400 µg/mL	Candida Species (*C. guilliermondii, C. tropicalis C. krusei, C. parasilopsis C. glabrata , C. albicans* )	Gabriela et al. (2014)

Chalcone-induced suppression of O_2_ consumption in sensitive bacteria and prevention of NADH oxidation in bacterial membranes are the sources of chalcones’ antibacterial activity (Haraguchi et al., 1998b). The rhizome and root of the *Glycyrrhiza* species, liquorice, is a generally used botanical drug to cure a variety of ailments, such as gastrointestinal issues and arthritis (Pastorino et al., 2018). More than 600 bioactive metabolites were extracted from liquorice to date, including many retrochalcones such as licochalcone A, B, C, D, E, etc., (Yoon et al., 2007). The absence of a -OH moiety at the C-2′ and C-6′ sites make these retrochalcones differ from regular chalcones and makes them members of an uncommon phenolic family (Xiao et al., 2019). Retrochalcones are recognized for their photo reactivity due to α,β unsaturation. They can undergo photo-induced *trans*-to-*cis* isomerization via delocalization of electron, which is made possible by the conjugated carbonyl function. Licochalcone A effectively inhibits Tumor Necrosis Factor (TNF)-α, Interleukin (IL)-1β, and IL-6, three markers of inflammation. Licochalcone A, B, C, and D have demonstrated antiviral, antitrypanosomal, anti-cancer, anti-inflammatory, antidiabetic, and antibacterial properties (Rudrapal et al., 2021). In 1975, Saitoh discovered licochalcone A, a phenolic chalcone with two aromatic rings acting as the main structural unit from the root of *Glycyrrhiza uralensis*. Two chemically reactive double bonds are present in licochalcone A and its isomers: (1) the α,β unsaturation, which promotes *trans*-to-*cis* isomerization by absorbing long wavelength light; and (2) aliphatic side chain unsaturation, which can result in ring-closing with the -OH group at C-4 (Rozmer and Perjési, 2016; Ara et al., 2024). One of the main chalcones isolated from liquorice, licochalcone A, has been exposed to have numerous advantageous pharmacological activities, such as anti-inflammation, antioxidation, anti-cancer, antimicrobial properties etc., (Li M.-T. et al., 2022). Tsukiyama et al. reported that salt-, heat-, and protease-resistant licochalcone A exhibited antibacterial properties towards Gram-positive bacteria, particularly *Bacillus* species. The authors noted that *in vitro*, licochalcone A completely suppressed *Bacillus subtilis’*s vegetative cell development at concentrations of up to 3 μg/mL (Table 2). With minimum inhibitory concentrations (MICs) of 2 ∼ 3 μg/mL, licochalcone A exhibited efficacy towards all tested gram-positive bacteria, particularly against *Bacillus* species. However, at 50 μg/mL, it was ineffective against gram-negative bacteria (Tsukiyama et al., 2002). Moreover, licochalcone A, especially extracted from the *Glycyrrhiza uralensis* explored as an antimicrobial metabolite as it inhibits the growth of several species of *Mycobacterium* as well as *Legionella* with concentrations of 1–4 mg/L (Table 2) (Friis-Møller et al., 2002).

Belofsky et al. isolated and characterized 4′,6′-dihydroxy-3′,5′-dimethyl-2′-methoxychalcone along with six metabolites from the organic extracts of *Dalea versicolor*. Using NMR and HRMS methods, the extracted metabolite structures were identified. At very small doses (∼3.3 μg/mL), 4′,6′-dihydroxy-3′,5′-dimethyl-2′-methoxychalcone completely inhibited the growth of *S. aureus* when combined with a subinhibitory quantity of berberine (Table 2). Furthermore, 4′,6′-dihydroxy-3′,5′-dimethyl-2′-methoxychalcone was observed to enhance the effects of prescribed antibiotics berberine and some antibiotics (erythromycin and tetracycline); action mechanism of 4′,6′-dihydroxy-3′,5′-dimethyl-2′-methoxychalcone was consistent with blocking the NorA MDR efflux pump in *S. aureus* (Belofsky et al., 2004).

First isolated from *Psoralea corylifolia* in 1968, isobavachalcone is a prenylated chalcone (Bhalla et al., 1968). Sugamoto et al. synthesized and characterised prenyl or geranyl groups containing naturally occurring chalcones such as xanthoangelol F, bavachalcone, 4-hydroxyderricin, deoxyxanthoangelol H, xanthoangelol, xanthoangelol H, isobavachalcone, and broussochalcone B and assessed their antibacterial activities towards both gram-negative (*Pseudomonas fluorescens, Proteus mirabilis, Escherichia coli*) and gram-positive bacteria (*Staphylococcus epidermidis, Bacillus subtilis, Micrococcus luteus*). The chalcones were also prepared by the use of montmorillonite K10 as a catalyst in the [1,3]-sigmatropic rearrangement of 2′-prenyloxyacetophenone, 2′-prenyloxychalcones, or 2′-geranyloxychalcones. Although xanthoangelol, 4-Hydroxyderricin, bavachalcone, isobavachalcone, xanthoangelol F, and broussochalcone B were active against gram-positive bacteria, but displayed no activities towards gram-negative bacteria (Table 2). SAR study designated that prenyl group on the A-ring contributes to a rise in antibacterial action and 3′-geranylchalcone with 4′-hydroxy moiety containing xanthoangelol exhibited strong activity (Sugamoto et al., 2011). Isobavachalcone, kamalachalcone E, and geranyl-substituted chalcone derivatives etc. have been tested against various pathogenic fungal strains like *Candida albicans, Cryptococcus neoformans, Trichophyton mentagrophytes, Cladosporium cladosporioides, Aspergillus fumigates,* etc., (Bhakuni and Chaturvedi, 1984; ElSohly et al., 2001; Jayasinghe et al., 2004; Kulkarni et al., 2014). The ability of chalcone compounds to interact with intracellular thiols determines their antimicotic activity against *Candida albicans*. Many of the natural and synthetic chalcones inhibit the conversion of tubulin into microtubules, making them toxic for the growth and survival of fungus (Elias et al., 1999; Go et al., 2005).

Chalcone derivatives are found active against many protozoan species of genus *Leishmania*, *Plasmodium* responsible for leishmaniasis and malarial disease, respectively in humans (Sen and Chatterjee, 2011; Kumar et al., 2013). The antiprotozoal activity of licochalcone A is very well studied. It is reported to inhibit the development of *Leishmania major* and *Leishmania donovani* promastigotes and amastigotes germs and markedly reduces the contamination of cells (Table 2) (Chen et al., 1993). When it was administered in *Plasmodium yoelii* infected mice through intraperitoneal or oral route, the mice survived from the fatal *Plasmodium yoelii* infection (Table 2) (Chen et al., 1994). Licochalcone A reported to bring ultrastructural changes in leishmania cells, impairs respiratory function by inhibiting mitochondrial dehydrogenase, bc1 complex, complex II etc., (Zhai et al., 1995; MI-ICHI et al., 2005). First reported in 1993, Chen and his group provided proof of the antimalarial attributes of licochalcone A with strong activity towards human pathogenic protozoan *Leishmania* species, highlighting the potential of chalcones as an antimalarial drug. Furthermore, it was found that licochalcone A inhibited the growth of *Plasmodium falciparum* which is susceptible and resistant to chloroquine. In mice infected with *Plasmodium yoelii* YM, intraperitoneal injection of 15 mg/kg four times a day for 3 days resulted in a 93% clearance of parasites without any side effects. In the same experiment, oral lichochalcone A dosages of 450, 150, and 50 mg/kg/day were shown to almost completely eradicate the parasitemia, and by the end of the 21-day trial, there was no mortality (Table 2) (Chen et al., 1994).Lichochalcone A preferentially inhibits fumarate reductase (FRD: an enzyme that binds to membrane to catalyze the reduction of fumarate to succinate) in the respiratory system of the parasite, changing the ultrastructure as well as the mitochondrial function of the parasite (Zhai et al., 1995). Licochalcone A had an inhibitory impact on human pathogenic *Legionella* and *Mycobacteria* species. *Legionella dumoffii*, *Legionella bozemanii*, and other species were suppressed at concentrations of 1–4 mg/L, whereas *Mycobacterium bovis*, *Mycobacterium tuberculosis*, and BCG were repressed by less than 20 mg/L (Friis-Møller et al., 2002). The antimalarial effectiveness of licochalcone A was further demonstrated by Mi-Ichi et al. when they reported that the parasite *Plasmodium yoelii* was eliminated in mice by licochalcone A without causing any harmful side effects. The negligible IC_50_ results (0.10 µM) for licochalcone A suggested that the suppression of the *Plasmodium* bc_1_ complex (ubiquinol-cytochrome *c* reductase) may account for a significant portion of its antimalarial action (Table 2) (MI-ICHI et al., 2005).

Other chalcones reported for antiprotozoal activity include kanzonol C, isocordin, 5-prenylbutein, 5-deoxyabyssinin II, crotaorixin, medicagenin, xanthohumol, etc., (Christensen et al., 1994; Torres-Santos et al., 1999; Narender and Gupta, 2004; 2005; Yenesew et al., 2004; Frölich et al., 2005; Salem and Werbovetz, 2005; Borges-Argáez et al., 2007; Garcia et al., 2021). Some of these compounds impair with uptake of hypoxanthine, thymidine, interfere with the biosynthesis of polyamines, and haemin degradation leads to death of protozoan cell. Verzele et al. first characterized the structure of xanthohumol, but it was in the 1990s that the pharmacological benefits of xanthohumol were recognized (Verzele et al., 1957). The structure of xanthohumol comprised of a chain of flavonoids, one unsaturated double bond (α, β), a prenyl motif, and two aromatic rings substituted with -OH and -OCH_3_ moities organized in a *trans* position. Because of the existence of α,β-unsaturated ketone moiety, xanthohumol possesses pharmacological properties. Prenyl units and the -OCH_3_ group replace the aromatic ring in this molecule, making it more lipophilic and having a strong affinity for biological systems’ membranes (Oledzka, 2024). Xanthohumol and *iso*-xanthohumol exerts antiviral activity towards bovine viral diarrhea virus (BVDV), Hepatitis C virus (HCV), Rhinovirus, Herpes simplex virus type 1 (HSV-1) and type 2 (HSV-2) at micro-molar concentration (BUCKWOLD et al., 2004). The antiviral property of crude hop extracts and purified hop constituents was examined by Buckwold et al. None of the extracts were able to stop Human immunodeficiency viruses (HIV), Influenza (Flu)-A and B, Respiratory syncytial virus (RSV), or Yellow Fever Virus (YFV) from replicating. With an IC_50_ in the negligible µg/mL range, a xanthohumol contained hop extract showed mild to average antiviral efficacy towards BVDV (therapeutic index (TI) = 6.0), HSV-2 (TI = >5.3), Rhino (TI = 4.0), and HSV-1 (TI = >1.9). Xanthohumol was shown to be responsible for the antiviral action seen in the xanthohumol contained hop extract towards BVDV, HSV-1, and HSV-2 using ultra-pure preparations (>99% pure). Structure activity relationship study indicated that compared to the isomer *iso*-xanthohumol, xanthohumol was more effective antiviral agent towards several viruses. Furthermore, xanthohumol demonstrated antiviral efficacy towards CMV, indicating the possibility of a broader anti-herpesvirus antiviral effect (Table 2) (BUCKWOLD et al., 2004).

**FIGURE 5:**
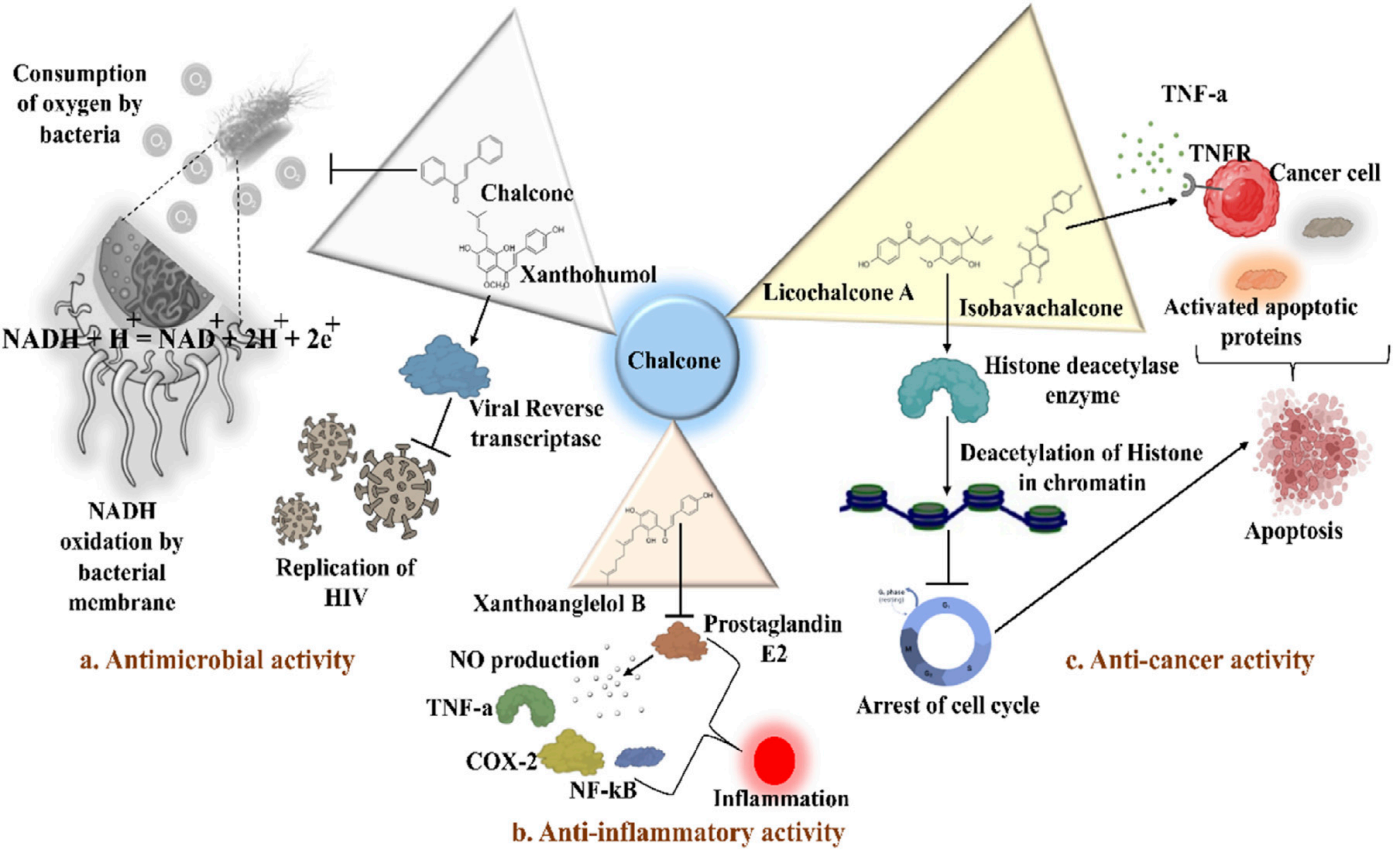
Distinct roles of naturally isolated chalcones as an antimicrobial, anti-inflammatory, and anti-cancer agent. Mechanism of different chalcones isolated from the natural sources. **(a)** Modulating the microbial membrane’s electron transport mechanism to inhibit the growth of microbes; **(b)** Regulating the enzymatic activity to control the inflammation; **(c)** Controlling the expression of several signaling proteins, and cell cycle phase transitions to eradicate the cancer cells.

Xanthohumol (Figure 5) and other natural chalcones inhibit HIV-1 replication by modifying the action of viral reverse transcriptase (Figure 5) and inhibit HIV-1-induced cytopathic effects (Wu et al., 2003; WANG et al., 2004). Zheng and his group extracted xanthohumol from the hop *Humulus lupulus* and assessed its anti-HIV-1 efficacy. The authors attribute that, at non-cytotoxic concentrations, xanthohumol suppressed reverse transcriptase, viral p24 antigen synthesis, and cytopathic effects generated by HIV-1 in C8166 cells. The EC_50_ values for RT generation and the inhibition of HIV-1 p24 antigen synthesis were 0.50 μg/mL (1.22 µM) and 1.28 μg/mL (3.21 µM), respectively. Furthermore, with an EC_50_ of 20.74 μg/mL, xanthohumol suppressed HIV-1 replication in peripheral blood mononuclear cell (PBMC) (Table 2). Lee and his colleagues isolated sixteen flavonoids and their derivatives from *Desmos* spp. and in H9 lymphocyte cells for their ability to prevent HIV replication. It was found that β-Hydroxy chalcone 2-Methoxy-3-methyl-4,6-dihydroxy-5-(3′-hydroxy)cinnamoylbenzaldehyde showed a favorable therapeutic index (TI) and strong anti-HIV property (EC_50_ = 0.022 μg/mL) (Table 2) (Wu et al., 2003). The SAR study demonstrated that the chalcone skeleton’s C-2 methoxy group might be essential for its anti-HIV properties.

Isoliquiritigenin, echinatin, and many other chalcones have been found to act against the influenza infection. They show strong inhibitory activity against neuraminidase activation and enhance the efficacy of antiviral drugs like oseltamivir (Ryu Y. B. et al., 2010; Dao et al., 2011; Park et al., 2011). Ryu et al. extracted eighteen polyphenols, including four chalcones from methanol extracts of *Glycyrrhiza uralensis* roots, and explored their neuraminidase repressive action. Experimental results suggested that isoliquiritigenin with an IC_50_ values of 9.0 µM among the chalcones had potent inhibitory activity. SAR studies demonstrated that the properties of chalcones are higher compared to their corresponding glycosides. Furthermore, methylation at the 2-OH reduced the inhibitory action, while increased -OH moieties at the 2 and 4′positions of chalcones augmented the repressive action (Table 2) (Ryu et al., 2010). Dao and associates isolated a novel licochalcone G and seven recognized chalcones from the acetone extract of *Glycyrrhiza inflata* and examined their anti-influenza activities. The chalcones’ structure was characterised by 1D and 2D NMR analysis, and it was validated by contrasting the spectroscopic and physicochemical analysis with those reported in the literature. With an IC_50_ = 2.49 ± 0.14 μg/mL, the most active chalcone echinantin, suppressed the neuraminidase (NA) produced from the new H1N1 influenza. Interestingly, echinantin maintained its potency in suppressing the H274Y mutant form’s activity, having an IC_50_ = 2.19 ± 0.06 μg/mL (Table 2) (Dao et al., 2011). Furthermore, the repressive activity of oseltamivir, a recognized competitive inhibitor in the presence of echinantin (at 1.35 μg/mL or 5 µM) was boosted remarkably on NAs of H9N2 (3.6-fold), H1N1 (7.0-fold), novel flu (WT) (3.7-fold), and tamiflu-resistant novel flu (H274Y) (52.6-fold) having IC_50_ from 4.94, 39.74, 21.09, and 5,132.85 ng/mL to 1.39, 5.69, 1.96, and 97.67 ng/mL, respectively. The authors assume that echinantin and oseltamivir may bind to distinct locations on the free and product-bound enzymes, each of which may function through a different inhibitory mechanism to cooperatively decrease NA activity.

Park et al. isolated a new chalcone xanthokeistal A having rare alkyl substitution with 6,6-dimethoxy-3-methylhex-2-enyl moiety along with five chalcones from *Angelica keiskei* and evaluated their potency against influenza virus neuraminidase inhibition (Table 2) (Park et al., 2011). With an IC_50_ of 12.3 µM, the most effective repressive effect was demonstrated by 2-hydroxy-3-methyl-3-butenyl alkyl (HMB) substituted chalcone xanthoangelol D. SAR studies indicated that for NA inhibition, the potency of substituted alkyl groups was as follows: HMB > 6-hydroxyl-3,7-dimethyl-octa-2,7-dienyl > dimethylallyl > geranyl.

Phenolic chalcones, for example, licochalcone A present in *Glycyrrhiza spp*. (particularly in the root region), have been investigated for their potential antimicrobial action towards *Streptococcus mutans*, *Lactobacillus buchneri*, and *Staphylococcus aureus*. *Glycyrrhiza inflata*, one of the two species of *Glycyrrhiza*, exhibits antimicrobial activity (MIC) at 25–250 μg/mL, while *Glycyrrhiza glabra* exhibits antimicrobial activity at 12.5–25 μg/mL (Table 2) (van Dinteren et al., 2022).

A study was conducted to isolate the new dimeric chalcone, kamalachalcone E, together with other compounds, including 1-(5,7-dihydroxy-2,2,6-trimethyl-2H-1-benzo-pyran-8-yl)-3-phenyl-2-propen-1-one, rottlerin, and 4′-hydroxyrottlerin, and studied the antifungal properties towards the *Cryptococcus neoformans*, and *Aspergillus fumigatus*. The structure of newly isolated kamalachalcone E was systematically characterised through 1D and 2D NMR studies, including HSQC, HMBC, COSY and ROESY experimentations. Interestingly, kamalachalcone E and 1-(5,7-dihydroxy-2,2,6-trimethyl-2H-1-benzo-pyran-8-yl)-3-phenyl-2-propen-1-one exhibited inhibitory property towards *Aspergillus fumigatus*, and *Cryptococcus neoformans*, and respectively with concentrations of 8, 4, and 16 μg/mL (Table 2). Interestingly, 4′-hydroxyrottlerin inhibited Thp-1 cell line proliferation by 54% at 100 μg/mL (Kulkarni et al., 2014). Similarly, another study was also conducted for antifungal study against different *candida* species for instance, *C. krusei, C. albicans, C. glabrata, C. guilliermondii, C. parasilopsis*. To delve into this study, *Zuccagnia punctata* Cav was taken and prepared it’s extract where 2ʹ,4ʹ-dihydroxy-3ʹ-methoxychalcone and 2ʹ,4ʹ-dihydroxychalcone have been isolated. These isolated chalcones were evaluated to prevent the growth of the above *candida* species. The MIC to eradicate 50% of the *candida* species population was 400 μg/mL (Table 2) (Gabriela et al., 2014).

Naturally occurring chalcones exhibit potent antibacterial, antifungal, antiviral, and antiprotozoal properties. Specific chalcones like pinocembrin chalcone, licochalcones, xanthohumol, and isoliquiritigenin have shown effectiveness against various bacteria, fungi, viruses, and protozoa. According to recent SAR studies, chalcone’s lipophilicity is influenced by the prenyl moiety on the A-ring and phenolic -OH groups, which is the reason for its antibacterial activity (Wang et al., 2023). Furthermore, the -OH group in the A ring’s 2-position and the prenyl group in the A ring’s 3-position boost the activity. In the 5′-position of the B ring, the propyl, prenyl, and hexyl groups are advantageous. Removing the prenyl moiety from the 5′-position of the B ring and methylating the -OH in the 4-position of the A ring both reduce activity. Prenyl moieties at the 3′and 2-positions of B rings and glycosyl in the A ring decrease the activities; if the prenyl group at the chalcones A-ring is further cyclized or oxygenated, the action will drop dramatically (Cui et al., 2015). They work by inhibiting microbial growth, disrupting cellular functions, and enhancing the efficacy of existing antimicrobial drugs. Their broad-spectrum activity highlights their potential as powerful natural antibiotics and therapeutic agents.

### 4.2 Tumor cell toxicity and chemopreventive attributes of naturally occurring chalcones

Cancer is the 2nd leading cause of mortality worldwide and is accountable for nearly one in every four premature deaths (22.8%) among those caused by noncommunicable diseases (NCDs: Such types of diseases that cannot be transmitted from one person to another) (Kocarnik et al., 2022). Globally, cancer claimed 9.7 million deaths in 2022, with an estimated 20 million new cases having been diagnosed (Siegel et al., 2022; Adhikari et al., 2025). Numerous factors might lead to cancer, and one of the most significant ones is chronic inflammation, which is related to the progression of cancer metastasis through dysregulating several cell signalling pathways (Nigam et al., 2023; Nath et al., 2025). Although platinum-based medication is one of the most advanced and widely used medications in clinical settings for treating a variety of human cancer types, it has significant adverse effects that limit its therapeutic usefulness (Nath et al., 2022; Adhikari et al., 2024a). As a result, drug resistance is becoming more and more widespread (Adhikari et al., 2019; Bhattacharjee et al., 2022; Das et al., 2023a; Nath et al., 2024). Plant-based drug development also gave rise to a stage for harmless anti-tumor medications by fully understanding the synergistic relationship between several anti-tumor botanical drugs or metabolites (Kaddah et al., 2021; Asma et al., 2022). The anti-cancer activities of more than 3,000 plant-based natural metabolites have been found. Among them, chalcone derivatives have demonstrated more cytotoxicity against various cancer cells than normal cells in both *in vitro* and *in vivo* studies, showing promising potential for anti-cancer therapeutics development (Ouyang et al., 2021). Furthermore, according to epidemiological research, eating a diet high in chalcones may lower your chance of developing malignancies in the breast, colon, lung, prostate, and pancreas (Prakash et al., 2013).

Tumor cytotoxicity and chemoprevention are among the enjoyable pharmacological activities of chalcones. Chemoprevention means preventing cancer from developing or delaying it with the use of various substances that impede cancer-initiating events (Benetou et al., 2015; Das et al., 2023b; Adhikari et al., 2024b; Bhattacharjee et al., 2024). It was reported that chalcones show inhibitory properties at micromolar concentrations by showing antimitotic activity; they arrest the cell cycle progression, inhibit transcription factors, induce mitochondrial uncoupling, and cause cellular apoptosis (Sharma et al., 2015). Chalcone treatment often leads to apoptosis of tumor cells via DNA disruption pathway characterized by nuclear condensation, DNA fragmentation, hypodiploid state, and upregulation of retinoblastoma (Rb) protein in tumor cells (Ramaiah et al., 2011). Several chalcones, such as Isobavachalcone (Figure 5), butein, licochalcone A, and xanthohumol, have been reported to enhance apoptosis in tumor cells by recruiting tumor necrosis factor-related apoptosis-inducing ligand (TRAIL: protein that may associate with certain different molecules in some cancer cells and responsible for inducing the apoptosis) (Figure 5) (Szliszka et al., 2009). Chalcones also inhibit histone deacetylase enzymes (HDACs: One type of evolutionarily conserved enzyme that aids in removing the acetyl groups from histones), blocking the deacetylation of histones in chromatin, causing changes in gene expression, resulting in cell cycle arrest, differentiation, and apoptosis of tumor cells (Kahyo et al., 2008; ORLIKOVA et al., 2012). Chalcones also hinder the initiation of nuclear factor kappa B (NF-κB: transcription factor that regulates the variety of cellular functions associated with promoter and enhancer regions of genes) as HDACs control the expression of the transcription factor NF-κB (ORLIKOVA et al., 2012).

Chalcones have been reported to hinder angiogenesis and cancer metastasis by controlling multiple signaling pathways. Natural chalcones originated from regulating the expression of many angiogenic factors, including epidermal growth factor receptor (EGFR: transmembrane protein of epidermal growth factor family), matrix metalloproteinases (MMPs: calcium-dependent zinc-containing endopeptidases that remodel the extracellular matrix proteins), vascular endothelial growth factor (VEGF), and also inhibit many numbers of signaling paths, for example, extracellular signal-regulated kinase (ERK)-1/2, NF-κB, and phosphoinositide-3-kinase–protein kinase B (P13-K/Akt: Cell signaling proteins that is responsible to enhance the growth of cancer cells) (MOJZIS et al., 2008). Isoliquiritigenin is an important chalcone derived from licorice root with promising anti-cancer action towards several malignant cells (Li et al., 2009; Bruyère et al., 2011; Wang et al., 2021). Isoliquiritigenin prevents migration and invasion in various tumor cells and demonstrates strong anti-cancer efficacy via several pathways, including apoptosis induction, the reduction of proliferation, and/or autophagy. Another study revealed that isoliquiritigenin, extracted from the *Glycyrrhiza glabra* showed the inhibitory activity of human acute promyelocytic leukemia cell line (HL-60) proliferation as well as decreased ROS production with induction of monocytic differentiation in leukemia cells. The reported effective concertation of this metabolite on HL-60 cells is 10 μg/mL (Table 3) (Li et al., 2009). In human U373glioblastoma cells, isoliquiritigenin exhibited cytostatic activity because it could overcome the cancer cells’ innate resistance to pro-apoptotic stimuli (Table 3) (Bruyère et al., 2011). Treatment with isoliquiritigenin cause apoptosis induction in cancer cells by preventing their proliferation and reducing inflammation. By reducing Psi(m) that causes apoptosis and inhibiting proliferation via the ERK/p38MAPK pathway, Zhang et al. reported that isoliquiritigenin (IC_50_ = 87.0 µM) repressed the C4-2, LNCaP prostate melanoma cells (Table 3) (Zhang et al., 2010).

**TABLE 3:** Plant source, doses, and anti-cancer roles of different natural chalcones.

Name and structure of chalcone	Source	Smiles	Activity	Drug concentration	Cell-line/Animal model of cancer	Mechanism of action	References
Isoliquiritigenin	*Glycyrrhiza glabra* L. (Family: *Fabaceae*)	OC(C=C1O) = CC = C1C(/C=C/C2 = CC = C(O)C=C2) = O	Inhibit the cell proliferation and decrease production of intracellular ROS. Also encourage the monocytic differentiation in HL-60 leukemia cells	10 μg/mL	HL-60 (Cell line of Human acute promyelocytic leukemia)	Not known	Li et al. (2009)
Isoliquiritigenin	Synthesized, however the parent compound was from *Calotropis procera* (Aiton) W.T. Aiton (Family: *Asclepiadaceae*)	OC(C=C1O) = CC = C1C(/C=C/C2 = CC = C(O)C=C2) = O	Cytostatic effect of isoliquiritigenin delays the growth of U373glioblastoma	IC_50_ = 68 µM	U373 (Humanglioblastoma cell line)	Not known	Bruyère et al. (2011)
Isoliquiritigenin	*Glycyrrhiza glabra* L. (Family: *Fabaceae*)	OC(C=C1O) = CC = C1C(/C=C/C2 = CC = C(O)C=C2) = O	Prevent the proliferation of prostate tumor cells Effectively reduces ROS production	10–100 μmol/L	C4-2 and LNCaP	Activation of pathways including adenosine monophosphate (AMP)-activated protein kinase (AMPK) and ERK cascades	Zhang et al. (2010)
Isoliquiritigenin	*Glycyrrhiza uralensis* Fisch. ex DC. (Family: *Fabaceae*)	OC(C=C1O) = CC = C1C(/C=C/C2 = CC = C(O)C=C2) = O	Downregulate proliferation of human umbilical vein endothelial induced by VEGF.	5–20 µM	MDA-MB-231 and MCF-7 cells	It attenuated VEGF expression by inducing HIF-1a in breast cancer cells	Wang et al. (2013)
Xanthohumol	*Humulus lupulus* L. (Family: *Cannabaceae*)	O=C (/C=C/C1 = C(O)C=C(O)C=C1)C2 = C(OC)C=C(O)C(C/C=C(C)\C) = C2O	Anti-tumor activities with attenuation of colony formation, induced apoptosis, and reducing cell viability	20 μM	A549, H520, and H358/old athymic nude mice	Dephosphorylation of forkhead box class O 3a (FOXO3a) and p53 upregulated modulator of apoptosis (PUMA) genes inhibits the Akt activity	Li et al. (2022b)
Xanthohumol	*Humulus lupulus* L. (Family: *Cannabaceae*)	O=C (/C=C/C1 = C(O)C=C(O)C=C1)C2 = C(OC)C=C(O)C(C/C=C(C)\C) = C2O	It induces apoptosis by increasing the DNA-damage response	0.1–85 µM	SW620, SW480, and HT29	Activation ATM signaling pathway	Scagliarini et al. (2020)
Licochalcone A	*Glycyrrhiza inflata* Batalin (Family: Fabaceae)	OC1 = C(C(C) (C)C=C)C=C (/C=C/C(C2 = CC = C(O)C=C2) = O)C(OC) = C1	Antioxidant and anti-cancer activity	58.79 ± 0.05 μg/mL (with PBS) 46.29 ± 0.05 μg/mL (without PBS)	L-02 and HepG2	Attenuation of p38/JNK/ERK signaling path and initiation of apoptotic cell death	Chen et al. (2017)
Licochalcone A	*Glycyrrhiza uralensis* Fisch. ex DC. (Family: *Fabaceae*)	OC1 = C(C(C) (C)C=C)C=C (/C=C/C(C2 = CC = C(O)C=C2) = O)C(OC) = C1	Reduction of the proliferation of lung melanoma cells and induction of apoptosis	20 µM	A549 and H460	Suppress the expression of XIAP, Survivin, c-FLIPL, c-IAP1, c-IAP2, and RIP1 genes and attenuates the stability of Survivin, XIAP, RIP1 Suppression of ERK signaling proteins Downregulated activities of JNK pathway	Luo et al. (2021)
Licochalcone A	*Glycyrrhiza uralensis* Fisch. ex DC. (Family: *Fabaceae*)	OC1 = C(C(C) (C)C=C)C=C (/C=C/C(C2 = CC = C(O)C=C2) = O)C(OC) = C1	Exerted HIF-1 repressive action in hypoxic tumor cells	IC_50_ = 10.6 and 13.7 μM	HCT116, H1299, and H322	Reduction of hypoxia-induced HIF-1α accumulation It reduces the mitochondrial respiration-facilitated ATP production rate	Park et al. (2021)
Licochalcone A	*Glycyrrhiza glabra* L (Family: *Fabaceae*)	OC1 = C(C(C) (C)C=C)C=C (/C=C/C(C2 = CC = C(O)C=C2) = O)C(OC) = C1	Inhibition of PD-L1 expression	IC_50_ = 54 μM	A549, HeLa and Hep3B/BALB/c male nude mice	Inhibition of the interaction between p65 and Ras blocked the expression of PD-L1 Enhanced the activity of cytotoxic T cells to combat against the cancer cells	Liu et al. (2021)
Licochalcone A		OC1 = C(C(C) (C)C=C)C=C (/C=C/C(C2 = CC = C(O)C=C2) = O)C(OC) = C1	Anti-osteosarcoma activity	IC50 = 10.4 µM (MG63 cells)	MG63 cells	Induce cell cycle arrest at G2-M phase, and trigger the apoptosis	Rossi et al. (2022)
Licochalcone A		OC1 = C(C(C) (C)C=C)C=C (/C=C/C(C2 = CC = C(O)C=C2) = O)C(OC) = C1	Anti-osteosarcoma activity	IC50 between 5 and 20 µM (143B)	143B cells MG63 cells	Activate anti-cancer activity by inducing apoptosis and autophagy	Rossi et al. (2024)
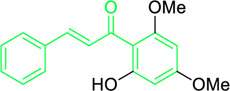 Flavokawain B	*Alpinia pricei* Hayata (Family: *Zingiberaceae*)	O=C(C1 = C(O)C=C(OC)C=C1OC)/C=C/C2 = CC = CC = C2	Flavokawain B caused apoptosis induction in melanoma cells, accumulation of cells in G2/M stage and autophagy	Significant activity at 25 and 50 μM concentration	HCT116	ROS production and GADD153 upregulation causes activation of mitochondrial apoptosis	Kuo et al. (2010)
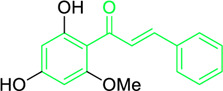 Cardamonin	*Artemisia absinthium* L (Family: *Asteraceae*)	OC1 = C(C(/C=C/C2 = CC = CC = C2) = O)C(OC) = CC(O) = C1	Anti-cancer activity	A375 (IC_50_ = 2.43 μM) NHEM (IC_50_ = 12.87 μM)	A375, NHEM, and NHDF cell lines	The dose-dependent enhanced caspase-3 activities and PARP cleavage Induce apoptosis in tumor cells	Berning et al. (2019)
2′,4'-dihydroxy-6′-methoxy-3′,5'-dimethylchalcone (DMC)	*Cleistocalyx operculatus* (Roxb.) Merr. & L.M.Perry (Family: *Myrtaceae*)	OC1 = C(C)C(O) = C(C(/C=C/C2 = CC = CC = C2) = O)C(OC) = C1C	Cytotoxicity and anti-proliferation activity	IC_50_ = 14.2 ± 0.45 μM EC_50_ = 3.3 ± 0.14 μM	K562 cell line	Suppress the Bcl-2 protein’s expression Not able to influence the Bax protein’s expression Lower ratio of Bcl-2/Bax and apoptosis induced	Ye et al. (2005)
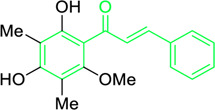 2′,4'-dihydroxy-6′-methoxy-3′,5'-dimethylchalcone (DMC)	Leaves of *Syzygium samarangense* (Blume) Merr. and L.M.Perry. (Family: *Myrtaceae*)	OC1 = C(C)C(O) = C(C(/C=C/C2 = CC = CC = C2) = O)C(OC) = C1C	Induction of cell proliferation, cell-cycle distribution, and apoptosis	40 μM	HCT116 and LOVO	Activated the cell cycle arrest at the G2/M phase	Ko et al. (2011)
2′,4'-dihydroxy-6′-methoxy-3′,5'-dimethylchalcone (DMC)	*Cleistocalyx operculatus* (Roxb.) Merr. and L.M.Perry (Family: *Myrtaceae*)	OC1 = C(C)C(O) = C(C(/C=C/C2 = CC = CC = C2) = O)C(OC) = C1C	Anti-cancer activity	IC_50_ = 10.5 ± 0.8 (PANC-1) and 12.2 ± 0.9 (MIA PACA2) µM	PANC-1 and MIA PACA2	Induced caspase-3 activation leads to apoptosis of PANC-1 cells Triggered degradation of caspase-3 as well as proteolytic initiation of caspase-3 and -9	Tuan et al. (2019)
2′,4'-dihydroxy-6′-methoxy-3′,5'-dimethylchalcone (DMC)	*Syzygium nervosum* (DC.) Kosterm (Family: *Myrtaceae*)	OC1 = C(C)C(O) = C(C(/C=C/C2 = CC = CC = C2) = O)C(OC) = C1C	Anti-cancer activities towards cervical cancer cells	IC50 = 15.76 ± 1.49 (C-33A), 10.05 ± 0.22 (HeLa), and 18.31 ± 3.10 (SiHa) µM	C-33A, HeLa, and SiHa	DNA disruption and cell cycle arrest in the G0/G1 phase by DMC treatment	Utama et al. (2022)
2′,4'-dihydroxy-6′-methoxy-3′,5'-dimethylchalcone (DMC)	*Cleistocalyx operculatus* (Roxb.) Merr. and L.M.Perry (Family: *Myrtaceae*)	OC1 = C(C)C(O) = C(C(/C=C/C2 = CC = CC = C2) = O)C(OC) = C1C	Potent cytotoxic effect against multi-drug resistant BEL-7402/5- FU cells	IC_50_ = 47.24 ± 0.46 μM (BEL-7402) and 44.23 ± 3.50 μM (BEL-7402/5-FU)	BEL-7402 and BEL-7402/5-FU (multi-drug resistance cell line)	Induced apoptosis leads to the enhancement of ROS generation Cell cycle arrest in the G1 stage Enhanced p53 gene’s expression with suppression of NF-κB signaling cascades	Ji et al. (2019)
2′,4'-dihydroxy-6′-methoxy-3′,5'-dimethylchalcone (DMC)	*Cleistocalyx nervosum* var. *paniala* (Roxb.) J.Parn. and Chantaranothai (Family: *Myrtaceae*)	OC1 = C(C)C(O) = C(C(/C=C/C2 = CC = CC = C2) = O)C(OC) = C1C	Anti-carcinogenic enzyme-inducing activity	IC_50_ = 15.76 µM (MRC5), and 15.10 ± 2.51 (SW620) µM	A549, HepG2, SW620, and MRC5/Male Wistar rats	Upregulation of detoxifying enzyme in rat livers	Vachiraarunwong et al. (2023)
2′,4'-dihydroxy-6′-methoxy-3′,5'-dimethylchalcone (DMC)	*Cleistocalyx operculatus* (Roxb.) Merr. and L.M.Perry (Family: *Myrtaceae*)	OC1 = C(C)C(O) = C(C(/C=C/C2 = CC = CC = C2) = O)C(OC) = C1C	Anti-cancer activities towards triple-negative breast cancer	IC_50_ = 34.95 ± 1.17 μM	MDA-MB-231, MCF 10A, and BT549 cells	Encouraged effective cell cycle arrest at G2/M stage Impair the microtubule polymerization through binding to β-tubulin protein Upregulation of pro-apoptotic proteins Bcl-2 related X protein (Bax) and caspase 3	Yu et al. (2024)
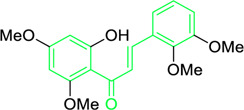 2′-hydroxy-2,3,4′,6′-tetramethoxychalcone (HTMC)	*Caesalpinia pulcherrima* (L.) Sw (Family: *Fabaceae*)	O=C (/C=C/C1 = C(OC)C(OC) = CC = C1)C2 = C(OC)C=C(OC)C=C2O	Arrest cell cycle in G1 phase. Suppression of A549 cell growth in *vitro* condition along with A549 cells facilitated tumor in Balb/c mice Inhibit phosphorylation of cell cycle regulatory protein cdc2 and Rb and cause accretion of tumor suppresser genes p53 and p21	12.5 µM *in-vitro* 1 mg/kg body weight of mice	A549 cell line Subcutaneously injected A549 cells mediated tumor in Balb/c mice	Suppression of phosphorylation of cell cycle regulatory protein cdc2/CDK1 and Rb	Rao et al. (2010)
2′,4′-dihydroxy-3′,5′-dimethyl-6′-methoxychalcone	*Syzygium samarangense* (Blume) Merr. and L.M.Perry (Family: *Myrtaceae*)	OC1 = C(C)C(O) = C(C(/C=C/C2 = CC = CC = C2) = O)C(OC) = C1C	Displayed potent antioxidant and cytotoxic activity	IC_50_ = 10 µM	SW-480	Not reported	Simirgiotis et al. (2008)
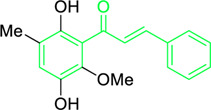 Stercurensin	*Syzygium samarangense* (Blume) Merr. and L.M.Perry (Family: *Myrtaceae*)	OC1 = C(C(/C=C/C2 = CC = CC = C2) = O)C(OC) = C(O)C=C1C	Displayed potent antioxidant and cytotoxic activity	IC_50_ = 35 µM	SW-480	Not known	Simirgiotis et al. (2008)
Cardamonin	*Syzygium samarangense* (Blume) Merr. and L.M.Perry (Family: *Myrtaceae*)	OC1 = C(C(/C=C/C2 = CC = CC = C2) = O)C(OC) = CC(O) = C1	Displayed potent antioxidant and cytotoxic activity	IC_50_ = 35 µM	SW-480	Not known	Simirgiotis et al. (2008)
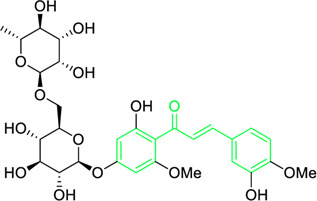 Hesperidin methyl chalcone	Semi synthetic	OC1 = C(C(/C=C/C2 = CC(O) = C(OC)C=C2) = O)C(OC) = CC(O [C@@H]3O [C@H](CO [C@@H]4 [C@@H](O)[C@@H](O)[C@H](O)[C@@H](C)O4)[C@@H](O)[C@H](O)[C@H]3O) = C1	Inhibit the cell viability of A549 melanoma cells	IC_50_ = 51.12 µM	Ehrlich ascites carcinoma murine model	Not Reported	(M.D. Rizvi et al., 2023)
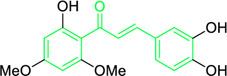 2′,3,4-trihydroxy-4′,6′-dimethoxychalcone (Chalcotatina)	*Chromolaena tacotana* (Klatt) R.M.King and H.Rob. (Family: *Asteraceae*)	O=C (/C=C/C1 = CC = C(O)C(O) = C1)C2 = C(O)C=C(OC)C=C2OC	Anti-proliferative, autophagic, and apoptotic activity	IC_50_ = 42.8 µM	Breast cancer cells (MDA-MB-231)	Constantly interacted with anti-apoptotic proteins Bcl-2	Mendez-Callejas et al. (2023)
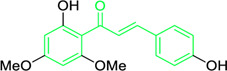 2′,4-dihydroxy-4′,6′-dimethoxy Chalcone	*Chromolaena tacotana* (Klatt) R.M.King and H.Rob. (Family: Asteraceae)	O=C (/C=C/C1 = CC = C(O)C=C1)C2 = C(O)C=C(OC)C=C2OC	Anti-breast cancer activity	IC_50_ = 52.5 (MCF-7) µM and 66.4 (MDA-MB-231) µM	MCF-7 and MDA-MB-231 cells	Induction of cell cycle arrest in the G0/G1 stage Activation of autophagic protein microtubule-related protein 1A/1 B-light chain 3-II (LC3-II)	Mendez-Callejas et al. (2024)

Isoliquiritigenin inhibits VEGF-induced proliferation of human umbilical vein endothelial cells (HUVECs) and also suppresses the sprouting of new blood vessels from VEGF-treated aortic rings in *ex vivo* studies. In addition, the administration of isoliquiritigenin in a dose-dependent manner in the mice with MDA-MB-231 xenograft tumor was able to diminish the growth of the tumor from day 16, with a 50%–65% reduction ratio than the vehicle groups. It is found to promote HIF-1α (Hypoxia-inducible factor-1α) to inhibit the expression of VEGF in breast cancer cells substantially and also interacted with VEGF receptor-2 (VEGFR-2) to block its kinase action (Table 3) (Wang et al., 2013).

Research has demonstrated the powerful antiaging, diabetic, inflammatory, antimicrobial, and cancer-preventing effects of xanthohumol, a prenylated chalcone in hop (*Humulus lupulus* L.). Growing data in recent years has indicated that xanthohumol has potent anti-cancer activities for several cancers, including glioblastoma, pancreatic cancer, hepatocellular carcinoma (HCC), thyroid cancer, cervical cancer, glioma, leukemia, breast cancer, cholangiocarcinoma (CCA), thyroid cancer, and ovarian cancer (Vesaghhamedani et al., 2022). Xanthohumol inhibits the development of cancer cells by inhibiting DNA synthesis, cell cycle arrest, and induction of apoptosis inhibition of aromatase activity (Miranda et al., 1999; Monteiro et al., 2007; Jiang et al., 2018). Li et al. and colleagues examined xanthohumol’s anti-cancer potential against human non-small cell lung cancer cells in both an *in vitro* and an *in vivo* model (Li et al., 2022). When xanthohumol was administered to A549, H520, and H358 cells in a dose-dependent method, the cells’ viability was considerably diminished. When exposed to the highest dose of 20 μM for 72 h, more than 85% of the cell viability was reduced, nearly preventing the cell growth. The number of colonies was significantly reduced after exposure to the highest dose of 20 μM for 72 h, with an inhibition rate on colony development of 95%. In the A549 (tumor volume of 221 mm^3^ compared to 632 mm^3^ in the control group) and H358 (tumor volume of 315 mm^3^ compared to 746 mm^3^ in the control group) xenograft models, xanthohumol at a dose of 10 mg/kg demonstrated excellent anti-tumor action, as the development of tumor was noticeably reduced (Scagliarini et al., 2020) (Table 3). In non-small cell lung cancer cells, xanthohumol triggered mitochondrial apoptosis by upregulating the expression of the p53-upregulated modulator of apoptosis. Anti-cancer attributes of xanthohumol towards colon cancer cells have also been evaluated (Scagliarini et al., 2020). In all three examined cell lines (SW480, SW620, and HT29), xanthohumol caused a potent and time-dependent reduction of cancer cell development starting at 5 µM. However, at concentrations greater than the previously established IC_50_, such as 30 μM, xanthohumol seemed toxic and inhibited many cells. Among the examined cell lines, xanthohumol was the most active towards SW620 cells (IC_50_ = 7 ± 1.38 μM after 72 h of treatment) (Table 3) (Scagliarini et al., 2020).

Interestingly, the DRI values of the anti-cancer drug 7-ethyl-10-hydroxycamptothecin indicated that synergistic interactions of xanthohumol with 7-ethyl-10-hydroxycamptothecin promoted the mortality of SW480 cells while potentially lowering the concentration of 7-ethyl-10-hydroxycamptothecin. The mechanistic study revealed that by triggering the ataxia telangiectasia mutated (ATM) pathway, xanthohumol exhibited its anti-cancer potential. Therefore, colorectal carcinoma (CRC) cells may become more sensitive to the anti-cancer drug 7-ethyl-10-hydroxycamptothecin, as a result of xanthohumol’s capacity to repair DNA disruption in melanoma cells.

Licochalcone A isolated from *Glycyrrhiza glabra* arrests cell cycle (Figure 5) in the G2/M stage, and causes apoptosis induction in several tumor cells (Deng et al., 2023). Treatment with licochalcone A inhibits phosphorylation of Rb, declines expression of transcription factor E2F, simultaneously reduces cyclin D1, and downregulates cyclin-dependent kinases (CDKs: cell cycle-regulating checkpoint proteins) 4 and 6, etc., (Fu et al., 2004). Chen and his group reported that HepG2 cells were repressed by licochalcone A in a dose-dependent way (Table 3) (Chen et al., 2017). This suppression was achieved by stopping the proliferation of cells and triggering apoptosis. In HepG2 cells, licochalcone A directly reduced MAPK signaling pathways, preventing proliferation and triggering apoptosis.

A study examined the anti-neoplastic activity of licochalcone A towards non-small cell lung carcinoma (NSCLC) cells (A549, H460, SPC-A1, H23, and H1299) (Table 3) (Luo et al., 2021). Using flow cytometry, it was confirmed that licochalcone A-induced apoptosis in A549 and H460 cells. In A549 and H460 cells, licochalcone A distinctly and time-dependently stimulated p38 and ERK. In addition, licochalcone A reduced the autophagy that was triggered by licochalcone A and inhibited jun N-terminal kinase (JNK: a cell signalling kinase protein that regulates the regulation of cellular senescence) activity. It also repressed the expression of cellular inhibitor of apoptosis protein 1 (c-IAP1), c-IAP2, X-linked inhibitor of apoptosis protein (XIAP), Survivin, cellular FLICE (FADD-like Il-1β-converting enzyme)-inhibitory protein (c-FLIPL), and receptor-interacting protein-1 (RIP1).

Mitochondrial malfunction is closely allied with the initiation of the mitochondrial apoptosis pathway. Park and colleagues reported that licochalcone A is the most prevailing bioactive metabolite in *G. uralensis*, which reduced the cancer cells’ growth and the activation of HIF-1α mediated by hypoxia (Park et al., 2021). Among the tested five major constituents of *Glycyrrhiza uralensis,* licochalcone A most effectively repressed HCT116 cell viability, having a GI_50_ value of 10.5 μM (Table 3). Moreover, licochalcone A demonstrated decreased viability of cells linked to tumor angiogenesis, such as smooth muscle cells (IC_50_ = 13.7 μM) and vascular endothelial cells (IC_50_ = 10.6 μM). Licochalcone A (2.5–25 μM) decreased ATP production and triggered mitochondrial disruption in H1299 and H322 lung melanoma cells by suppressing hypoxia-induced HIF-1α accretion and the expression of target genes glucose transporter 1 (GLUT1) and phosphoinositide-dependent kinase 1 (PDK1), leading to the instigation of the mitochondrial apoptosis and cancer cell apoptosis.

Liu and associates studied the anti-cancer activity both *in vitro* and *in vivo* of licochalcone A (Liu et al., 2021). In a tumor and T cell coculture model, licochalcone A inhibited the expression of programmed cell death ligand 1 (PD-L1), restoring T lymphocyte function. Flow cytometry result revealed that as the concentration of licochalcone A increased, the percentage of programmed cell death ligand 1 positive HCT116 cells decreased from 20.3% to 9.9%. Importantly, mice bearing HCT116 xenograft tumors were given licochalcone A, which suppressed tumor growth without causing cytotoxicity (Table 3). Additionally, it was also observed that licochalcone A inhibited the Ras/Raf/MEK and NF-κB signaling pathway, which is responsible for the proliferation of tumor cells.

Recently Rossi et al. investigated the anti-cancer efficacy of licochalcone A and several chalcone derivatives against multicellular tumor spheroids from MG63 and 143B osteosarcoma cell lines. In this study, it was also observed that licochalcone A able to arrest the cell cycle at G2-M phase in osteosarcoma cancer cells. Further, it induces the apoptosis to eradicate proliferation (Table 3) (Rossi et al., 2022). Remarkably, most of the chalcones had IC_50_ values between 5 and 20 µM against 143B osteosarcoma cell lines, indicating that they are all efficacious. Against the MG63 cells, licochalcone A at 10 µM inhibited the cell number to ∼40% compared to the control within 48 h. Furthermore, licochalcone A exhibited remarkable IC_50_ values of 10.4 µM against the MG63 cells (Table 3) (Rossi et al., 2024). Additionally, it was observed that after treating osteosarcoma cell lines with licochalcone A, it may function as an anti-proliferative agent by reducing cell invasion and activating apoptosis and autophagy.

Other potential chalcones with anti-cancer activity are isobavachalcone, xanthoangelol F, flavokawain B, cardamonin etc., (Akihisa et al., 2003; Kuo et al., 2010; Berning et al., 2019). Flavokawain B is a *trans*-chalcone substituted by -OH moiety at positions 2′and -OMe moieties at positions 4′and 6'. Flavokawain B, extracted from the *Alpinia pricei Hayata* displayed significant activity at concentrations of 25 and 50 μM to induce apoptosis and arrest the human colon cancer cell, HCT116, from G2 to M stage (Table 3). Moreover, this isolated metabolite is also capable of ROS generation with upregulation of the growth arrest and DNA damage-inducible gene 153 (GADD153). Therefore, Flavokawain B activates apoptosis based on mitochondria (Kuo et al., 2010). The chalcone cardamonin (2′,4′-dihydroxy-6′-methoxychalcone) was initially extracted from the flowers of *Artemisia absinthium* and it is frequently isolated from many plants in the Zingiberaceae family (Hatziieremia et al., 2006). Cardamonin can be isolated from plant sources using the micellar electrokinetic chromatography (MEKC) method (Liu et al., 2007). Kamiński and his group first reported the solid-state structure of cardamonin (Budziak et al., 2020). Two symmetry-independent molecules in the cardamonin crystal lattice are connected by hydrogen bonding and π···π stacking contacts, ensuing in two distinct conformations of the cardamonin molecules in the crystal structure. Furthermore, unlike in EtOH, where cardamonin mainly exists as monomers, cardamonin occurs in a dimeric state in water solutions. Cardamonin has been the topic of several analyses demonstrating its anti-cancer properties attributable to its capability to cause apoptosis in cancer cells. Cardamonin unveiled anti-cancer properties in different melanoma cell lines like lung cancer cells (A549, H460), ovarian cancer cells (SKOV3, A2780), gastric cancer cells (AGS, MGC-803, BGC-823), colon cancer cells (HCT-116, SW480, DLD1, LS174T), breast cancer cells (BT-549, SUM190, MCF7, COMA-1), prostate cancer cells (PC-3), colorectal cancer cells (HCT-15, HCT116, SW480, SW620), as well as nasopharyngeal cancer cells (CNE-1, CNE-2, HONE-1, SUNE-2), and leukemia (WEHI-3) etc., (Nawaz et al., 2020). Cardamonin suppresses the NF-κB pathway, which is known to generate reactive oxygen species (ROS). This affects cell development and triggers cell death in melanoma cells (Li et al., 2017). Additionally, it prevents the growth of cells by downregulating phosphorylated mammalian target of rapamycin (p-mTOR), protein kinase B (Akt/PKB), p70 Ribosomal Protein S6 Kinase (P70S6K), phosphatidylinositol 3-kinase (p-PI3K), and B cell lymphoma −2 (Bcl-2) (Shi et al., 2018). Berning and colleagues explored the anti-proliferative effect of cardamonin against A375 cancer cell lines and normal human epidermal melanocytes (NHEM) along with normal human dermal fibroblasts (NHDF) cell lines (Table 3) (Berning et al., 2019). Cardamonin had the most cytotoxic against A375 tumor cells (IC_50_ = 2.43 μM) and had less harmful effects against normal NHEM cell lines (IC_50_ = 12.87 μM). After 24 h of treatment, only around 5% of the tumor cells were viable, indicating that 20 µM of cardamonin had the maximum cytotoxic effect. The dose-dependent upsurge in caspase-3 actions and poly (ADP-ribose) polymerase (PARP) cleavage in the A375 cancer cells confirmed the induction of apoptosis, which was further established by the time-dependent rise in membrane blebbing following cardamonin treatment (Berning et al., 2019).

In recent day’s chemical engineering of chalcones manipulating the structure at aryl rings, addition of heteroaryl scaffolds, and conjugation with other molecules of pharmacological importance enhance the anti-cancer properties of chalcones (Karthikeyan et al., 2014). 2′,4′-Dihydroxy-6′-methoxy-3′,5′-dimethylchalcone (DMC), a chalcone present in leaves of *Syzygium samarangense*, seeds of *Syzygium nervosum* and buds of *Cleistocalyx operculatus*, exhibits potent anti-cancer properties against leukemia, liver, colorectal, pancreatic, and breast cancers (Ye et al., 2005). Similarly, Yang and his colleagues investigated the anti-cancer properties of 2′,4′-Dihydroxy-6′-methoxy-3′,5′-dimethylchalcone in LOVO and HCT116 human colorectal cancer cells (Table 3) (Ko et al., 2011). DMC repressed the cells growth dependent on concentration and time. Compared to the vehicle control, DMC suppressed cell growth in HCT116 and LOVO cells by 40% and 37%, respectively, after a 24-h treatment at a dosage of 40 μM. The authors ascribed that DMC can trigger autophagy and reduce the growth of HCT116 and LOVO cells by delaying the G2/M stage of the cell cycle. Tran and colleagues extracted 2′,4′-Dihydroxy-6′-methoxy-3′,5′-dimethyl chalcone from the *Cleistocalyx operculatus* buds and characterized by mass and NMR (^1^H and ^13^C) spectroscopy and inspected anti-cancer properties on pancreatic cancer cell lines (Tuan et al., 2019). *In vitro* analysis indicated that in concentration-dependent ways, DMC noticeably repressed the proliferation of PANC-1 (IC_50_ = 10.5 ± 0.8 μM) and MIA PACA2 (IC_50_ = 12.2 ± 0.9 μM) cells (Table 3). The authors attribute that by activating caspase-3, DMC caused PANC-1 to undergo apoptosis. DMC increased the level of bak protein, caused the proteolytic initiation of caspase-3 and -9, degraded the substrate proteins caspase-3, and reduced the expression of bcl-2 in PANC-1 cells. DMC was also extracted from the seeds of *S. nervosum* and was characterized by ^1^H-NMR, ^13^C-NMR spectra, and 2D-NMR experiments, including COSY, HSQC, and HMBC (Utama et al., 2022). DMC showed promising anti-cancer activities against HeLa (IC_50_ = 10.05 ± 0.22), C-33A (IC_50_ = 15.76 ± 1.49), and SiHa cells (IC_50_ = 18.31 ± 3.10 µM), while cisplatin showed an IC_50_ = 9.93 ± 0.16 µM against HeLa cells (Table 3). HeLa cells treated with DMC exhibited DNA disruption, reduced cell division, and instigated apoptosis. DMC’s anti-cancer potential against a multidrug-resistant HCC cell line was reported by Lu and colleagues (Ji et al., 2019). In comparison to the generally utilized anti-cancer drug 5-fluorouracil (5-FU) (IC_50_ = 69.96 ± 10.69 μM against BEL-7402 and IC_50_ = 5,662.82 ± 245.77 μM against BEL-7402/5-FU), DMC exhibited concentration-dependently growth inhibition of BEL-7402 (IC_50_ = 47.24 ± 0.46 μM) and BEL7402/5-FU cells (IC_50_ = 44.23 ± 3.50 μM), suggesting that the BEL-7402/5-FU cells were more sensitive towards DMC (Table 3). BEL-7402/5-FU (5-FU resistant cancer cell line) cells had a resistance index of 80.95 against 5-FU, indicating that while both cell lines were susceptible to DMC, BEL-7402/5-FU cells have been unaffected to 5-FU. The authors attribute that the mechanism of DMC towards the multidrug-resistant BEL-7402/5-FU hepatocellular cancer cells was found to be via upregulating the ROS production within the cells and the mitochondria-dependent apoptotic pathway. Furthermore, it was shown that DMC inhibited the advancement of the cell cycle during the G1 stage through lowering the expression levels of associated proteins, such as phospho-glycogen synthase kinase 3 beta (p-GSK3β), cyclin D1, and CDK4. Vachiraarunwong and co-workers accessed the anti-cancer activities against colorectal carcinoma of CH_2_Cl_2_ extract of DMC extracted from the seeds of *Cleistocalyx nervosum* (Vachiraarunwong et al., 2023). *In vitro* studies revealed that DMC-suppressed colorectal carcinoma SW620 cells (IC_50_ = 15.10 ± 2.51 μM) were comparable to 5-FU (IC_50_ = 16.70 ± 6.74 μM), but less cytotoxic than 5-FU against noncancerous MRC-5 cells having IC_50_ of DMC (15.76 µM) was threefold more compared to 5-FU (5.78 µM) (Table 3). By promoting the metabolization of xenobiotics and inhibiting cell growth, DMC showed a chemopreventive result in the initial phases of colorectal carcinogenesis. Recently, it was reported that DMC exhibited a potent concentration-dependent cytotoxic effect in MDA-MB-231 cells (IC_50_ = 34.95 ± 1.17 μM) compared to MCF10A, MCF-7, BT549, and MDA-MB-468 cells (Table 3) (Yu et al., 2024). DMC worked as an anti-tumor agent by causing G2/M arrest in MDA-MB-231 cells. It also triggered G2/M stage arrest by blocking microtubule polymerization by binding with β-tubulin. Furthermore, it increased the production of ROS by suppressing catalase activity, which in turn controlled the PI3K signaling pathway and jointly caused cell cycle arrest.

In addition to MRC-5, SV-40 transfected Beas2B, and WI-38 cells normal cell lines, 2′-hydroxy-2,3,4′,6′-tetramethoxychalcone (HTMC) derived from *Caesalpinia pulcherrima* was tested for its anti-cancer properties against A549, H1299, and H1355 cancer cells (Rao et al., 2010). According to *in vitro* research, 2′-hydroxy-2,3,4′,6′-tetramethoxychalcone selectively killed cancer-derived cells instead of normal cell lines. The sensitivity of HTMC to A549 (IC_50_ = 47 μM) was higher than that of H1299 (IC_50_ = 48 μM) and H1355 (IC_50_ = 76 μM) cells (Table 3). In line with its *in vitro* efficacy, HTMC demonstrated powerful anti-cancer properties in the A549 tumor model in Balb/c mice, resulting in a noteworthy 33% decrease in tumor volume and no drop in body weight, health issues, or behavioral abnormalities. The mechanistic study indicated that cell cycle arrest in the G1 stage by inhibiting phosphorylation of cell cycle regulatory protein cdc2/CDK1 and Rb leads to a reduction of A549 cell growth (Rao et al., 2010). *Syzygium samarangense* has been recognized as a source of pharmacologically active chalcones. Various *C*-methylated chalcones such as stercurensin, cardamonin, and 2′,4′-dihydroxy-3′,5′-dimethyl-6′-methoxychalcone extracted from *Syzygium samarangense* displayed potent antioxidant and cytotoxic activity in human colon cancer cell SW-480 (IC_50_ = 10, 35, and 35 μM, respectively) (Table 3) (Simirgiotis et al., 2008). The methylated byproduct of hesperidin extracted from citrus foods is hesperidin methyl chalcone, which has an open ring with several -CH_3_ group substitutions. The semi-synthetic hesperidin methyl chalcone is derived from hesperidin (hesperidin-7-rhamnoglucoside), which undergoes methylation in alkaline condition to produce hesperidin methyl chalcone. Methylation produces a more water-soluble molecule and thus increases its absorption and bioavailability. It has been investigated for use as an analgesic and anti-inflammatory in treating numerous disorders (Guazelli et al., 2021). Rizvi and colleagues explored the anti-cancer activities of hesperidin methyl chalcone *in vitro* and *in vivo*. With an IC_50_ of 51.12 µM, it exhibited strong anti-cancer efficacy in lung cancer cell lines similar to that of the anti-cancer drug hesperetin (IC_50_ = 49.12 µM) (Table 3). In the *in vivo* studies, it was found that the survival of Ehrlich ascites carcinoma (EAC)-bearing mice was noticeably extended by 15 days of treatment with hesperidin methyl chalcone than EAC-bearing untreated mice. Furthermore, in EAC-bearing mice, hesperidin methyl chalcone effectively exhibited a strong anti-cancer activity, as seen by a prominent reduction in tumor volume and weight (M.D. Rizvi et al., 2023). Flavonoids having antioxidant and anti-cancer attributes are found in *Chromolaena tacotana* (Klatt). Gina Mendez-Callejas and co-workers recently isolated and characterized a novel chalcone 2′,3,4-trihydroxy-4′,6′-dimethoxychalcone (chalcotatina) from the *C. tacotana* plant. This novel chalcone exhibits anti-proliferative properties in the MDA-MB-231 triple-negative breast cancer cell line, having an IC_50_ of 42.8 µM (Table 3). Chalcotanina demonstrated a remarkable selectivity for the triple-negative breast cancer (TNBC) cell line, as evidenced by selectivity index values of 6.9, 9.1, and 5.3 in comparison to MCF-12F, MRC-5, and BHK-21 cells, respectively. Chalcotanina triggers apoptosis by activating caspases 3/7 and induces autophagy by altering the mTOR protein’s structural shape. Additionally, it alters the potential of the mitochondrial membrane and downregulates Bcl-2 anti-apoptotic members, which activates the intrinsic pathway (Mendez-Callejas et al., 2023). The same research group isolated and characterized 2′,4-dihydroxy-4′,6′-dimethoxy-chalcone (DDC) from the *C. tacotana* plant. The structure of the extracted chalcone was characterized by NMR (^1^H NMR and ^13^C NMR) spectra and high-resolution electrospray ionization mass spectrometry (HR-ESI-MS). Promising anti-cancer properties were exhibited by DDC against breast cancer cell lines MCF-7 (IC_50_ = 52.5 µM) and MDA-MB-231 (IC_50_ = 66.4 µM) compared non-tumor MCF-12F cells (IC_50_ = 232.8 µM) (Table 3). Selectivity index (SI) results for MCF-7, and MDA-MB-231 were 4.4 and 3.5, respectively than positive controls, resveratrol and paclitaxel. These findings imply that DDC may target breast cancer cells more successfully while having less of an effect on healthy cells. In breast cancer cells, DDC causes cell cycle arrest in the G0/G1 stage, alters the mitochondrial outer membrane potential (∆ψm), and initiates the mitochondrial route of death (Mendez-Callejas et al., 2024).

The paragraphs summarize the anti-cancer properties of various chalcones, focusing on their capability to prevent tumor development by suppressing the Ras/Raf/MEK and NF-κB signalling pathways. The SAR investigations indicated that structural modifications of both aryl rings, substitution of heteroaryl scaffolds for aryl rings, and hybridization by conjugation with other pharmacologically potential motifs significantly increase the anti-cancer properties of chalcones (Karthikeyan et al., 2014).

Furthermore, methoxy substitutions on both A and B rings and their substitution pattern significantly impact the anti-cancer activity of chalcones (Mahapatra et al., 2015b). Licochalcone A inhibits PD-L1 expression and restores T lymphocyte function, slowing tumor growth without toxicity. Chalcones like cardamonin and DMC also showed strong anti-cancer activity across numerous cancer cells, including lung, colorectal, and breast cancers. Notably, HTMC selectively killed cancer cells over normal cells, showing higher sensitivity in A549 lung cancer cells. HTMC also demonstrated significant tumor reduction in mice without adverse effects. Other chalcones like cardamonin, hesperidin methyl chalcone, and chalcotatina displayed significant anti-cancer activity in several melanoma cells. These findings suggest the potential of naturally occurring chalcones in cancer treatment.

### 4.3 Antioxidant and anti-inflammatory properties of natural chalcones

Chalcones are extensively recognized due to their anti-inflammatory and antioxidant activities. Natural chalcones are phenolic in nature and include one or more phenolic -OH in their structure. This generally allows them to scavenge free radicals naturally, which can be beneficial when dealing with oxidative stress. Numerous studies conducted in this field have confirmed the relation between inflammation, oxidative stress, and carcinogenesis. Persistent oxidative stress has been shown to cause chronic inflammation, which in turn may act as a mediator for numerous chronic illnesses, such as cancer. Numerous transcription aspects, such as NF-κB, HIF-1α, β-catenin/Wingless and Int-1 (Wnt), nuclear factor erythroid 2-related factor 2 (Nrf2), and many more, are induced by oxidative stress. This can result in the expression of hundreds of different gene products, including chemokines, growth factors, cell cycle regulatory biomolecules, inflammatory cytokines, and anti-inflammatory biomolecules (Reuter et al., 2010). Such misshapen gene expression sometimes leads to the alteration of a normal cell into neoplastic cells with altered proliferation and survival characteristics.

Chalcones have been found to counter many cancer-initiating processes and prevent carcinogenesis. The chemopreventive capacity of chalcone compounds is linked to their strong antioxidant and anti-inflammatory activities in biological systems (Orlikova et al., 2011). Owing to their potent antioxidant activities, chalcone compounds can scavenge free radicals and check oxidative stress; therefore, they can modulate many biological processes like diabetes, aging, inflammation, ischemic injury, cancer, and neurodegenerative illnesses. Free radicals and ROS (a strong reactive oxygen species like diatomic oxygen (O_2_), and hydrogen peroxide that is responsible for generating oxidative stress by damaging the DNA, lipids, and proteins) are related in various phases of carcinogenesis (Liou and Storz, 2010). ROS is well known for its cancer-promoting activity. Chalcones’ ability to reduce ROS generation is therefore crucial in preventing carcinogenesis. Various chalcones isolated from plants like licochalcone B and D, broussochalcone A, xanthokeismins A, B, and C, xanthoangelol B, etc. eported to have strong superoxide anion and 2,2-diphenyl-1-picrylhydrazyl (DPPH) radical scavenging action (Haraguchi et al., 1998a; Cheng et al., 2001). Aoki et al. extracted and characterized three new *C*-geranylated chalcones, designated as xanthokeismins, from the *Angelica keiskei* stems (Aoki et al., 2008). The structures of the new *C*-geranylated chalcones were well characterized by FT-IR, NMR (^1^H, ^13^C NMR, ^1^H-^1^H COSY, DEPT, and HMQC) and mass (MALDITOFMS) spectroscopic methods. All the extracted chalcones showed significant superoxide scavenging property (IC_50_ = 0.51–1.1 µM) superior to the positive control resveratrol (IC_50_ = 5.3 μM). Among the newly isolated *C*-geranylated chalcones, xanthokeismin A had the most significant superoxide-scavenging property (IC_50_ = 0.51 ± 0.023 μM) (Table 4). Furthermore, chalcone compounds have been demonstrated to block prostaglandin E2 (PGE2: involved in several biological functions, mostly in neurological inflammatory diseases) and nitric oxide (NO) synthesis, thereby abrogating inflammatory stimuli and mitigating the impact of inflammation (Figure 5) (Nowakowska, 2007b).

**TABLE 4:** Plant source, doses, and antioxidative and anti-inflammatory roles of different naturally occurring chalcones.

Name and structure of chalcone	Plant source	Smiles	Activity	Dose of drug/metabolite	Targeted model	Mechanism of action	References
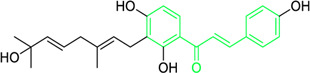 Xanthokeismins A 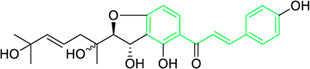 Xanthokeismins B 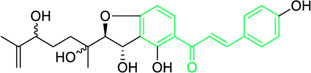 Xanthokeismins C	*Angelica keiskei* (Miq.) Koidz. (Family: *Apiaceae*)	O=C (/C=C/C1 = CC = C(O)C=C1)C2 = C(O)C(C/C=C(C)/C/C=C/C(C) (O)C) = C(O)C=C2 O=C (/C=C/C1 = CC = C(O)C=C1)C2 = C(O)C ([C@H](O)[C@H](O3)C(C) (O)C/C=C/C(C) (O)C) = C3C = C2 O=C (/C=C/C1 = CC = C(O)C=C1)C2 = C(O)C ([C@H](O)[C@H](O3)C(C) (O)CCC(O)C(C) = C) = C3C = C2	Antioxidant	IC_50_ = 0.51 ± 0.023, 0.69 ± 0.017, and 1.1 ± 0.12 µM respectively for A, B and C	*In vitro* model	Scavenges superoxide radical	Aoki et al. (2008)
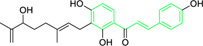 Xanthoangelol B	*Angelica keiskei* (Miq.) Koidz. (Family: *Apiaceae*)	O=C (/C=C/C1 = CC = C(O)C=C1)C2 = C(O)C(C/C=C(C)/CCC(O)C(C) = C) = C(O)C=C2	Antioxidant	IC_50_ = 0.92 ± 0.16 µM	*In vitro* model	Superoxide radical scavenging	Aoki et al. (2008)
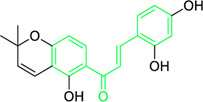 3″,3″-dimethylpyrano [3′,4′]2,4,2′-trihydroxychalcone	*Artocarpus communis*. J.R.Forst. and G.Forst. (Family: *Moraceae*)	O=C (/C=C/C1 = C(O)C=C(O)C=C1)C2 = C(O)C3 = C(OC(C) (C)C=C3)C=C2	Anti-inflammatory. Inhibits NO generation from LPS initiated RAW264.7 cells	IC_50_ = 18.8 µM	RAW264.7 cells	Inhibition of iNOS.	Han et al. (2006)
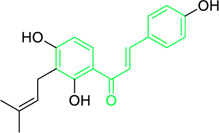 Isobacachalcone	*Artocarpus communis* J.R.Forst. and G.Forst. (Family: *Moraceae*)	OC1 = C(C(/C=C/C2 = CC = C(O)C=C2) = O)C=CC(O) = C1C/C=C(C)/C	Anti-inflammatory	IC_50_ = 6.4 µM	RAW264.7 cells	Inhibition of iNOS.	Han et al. (2006)
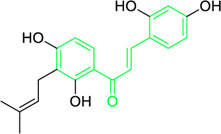 Morachalcone A	*Artocarpus communis*. J.R.Forst. and G.Forst. (Family: *Moraceae*)	O=C (/C=C/C1 = CC = C(O)C=C1O)C2 = C(O)C(C/C=C(C)\C) = C(O)C=C2	Anti-inflammatory	IC_50_ = 16.4 µM	RAW264.7 cells	Inhibition of iNOS.	Han et al. (2006)
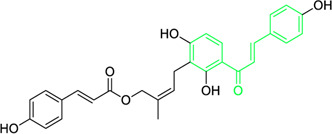 Gemichalcones B	*Artocarpus communis*. J.R.Forst. and G.Forst. (Family: *Moraceae*)	O=C (/C=C/C1 = CC = C(O)C=C1)C2 = C(O)C(C/C=C(C)\COC(/C=C/C3 = CC = C(O)C=C3) = O) = C(O)C=C2	Anti-inflammatory	IC_50_ = 9.3 µM	RAW264.7 cells	Inhibition of iNOS.	Han et al. (2006)
Xanthohumol	*Humulus lupulus* L (Family: *Cannabaceae*)	O=C (/C=C/C1 = C(O)C=C(O)C=C1)C2 = C(OC)C=C(O)C(C/C=C(C)\C) = C2O	Anti-inflammatory	0.5–5 μg/mL	BV2 cells	Upregulation of downstream HO-1 factors Induction of Nrf2-ARE signaling pathway	Lee et al. (2011)
Cardamonin	*Alpinia conchigera* Griff. (Family: *Zingiberaceae*)	OC1 = C(C(/C=C/C2 = CC = CC = C2) = O)C(OC) = CC(O) = C1	Anti-inflammatory Activity	50 mg/kg 30 µM	C57BL/6 Mice RAW264.7 cells	Suppression of Nuclear Factor-B signal cascade Suppresses the expression of TNF-α, NO and inducible NO synthase and COX-2	Lee et al. (2006)
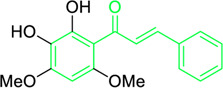 2′,3′-dihydroxy-4′,6′-dimethoxychalcone (DDC)	*Perilla frutescens* var. Crispa (Thunb.) H.Deane (Family: *Lamiaceae*)	OC1 = C(C(/C=C/C2 = CC = CC = C2) = O)C(OC) = CC(OC) = C1O	Antioxidant activity	Less than 30 μM	PC12 cells	Activated Nrf2-antioxidant response element (ARE)	Izumi et al. (2012)
Hesperidin methyl chalcone	Semi synthetic	OC1 = C(C(/C=C/C2 = CC(O) = C(OC)C=C2) = O)C(OC) = CC(O [C@@H]3O [C@H](CO [C@@H]4 [C@@H](O)[C@@H](O)[C@H](O)[C@@H](C)O4)[C@@H](O)[C@H](O)[C@H]3O) = C1	TNF α, IL-1β, IL-6, and IL-33 suppression in colon cells	Not reported	Male Swiss, C57BL/6 mice	Inhibits the colon’s NF-κB pathway	Guazelli et al. (2021)
Hesperidin methyl chalcone	Semi synthetic	OC1 = C(C(/C=C/C2 = CC(O) = C(OC)C=C2) = O)C(OC) = CC(O [C@@H]3O [C@H](CO [C@@H]4 [C@@H](O)[C@@H](O)[C@H](O)[C@@H](C)O4)[C@@H](O)[C@H](O)[C@H]3O) = C1	Analgesic, anti-inflammatory, and antioxidant properties	10, 30, or 100 mg/kg	Swiss mice	Attenuation of ROS production Impair macrophage NF-κB activation	Rasquel-Oliveira et al. (2020)
Hesperidin methyl chalcone	Semi synthetic	OC1 = C(C(/C=C/C2 = CC(O) = C(OC)C=C2) = O)C(OC) = CC(O [C@@H]3O [C@H](CO [C@@H]4 [C@@H](O)[C@@H](O)[C@H](O)[C@@H](C)O4)[C@@H](O)[C@H](O)[C@H]3O) = C1	Activate the Nrf2 signaling pathway Antioxidant activity	0.03–3 mg/kg	Swiss mice	Dose-dependent alteration of diclofenac by reducing urea and creatinine levels Reduce the IL-6, IFN-γ, and IL-33 expression Upregulation of IL-10 with reduction of kidney swelling, and urine NGAL.	Bussmann et al. (2022)
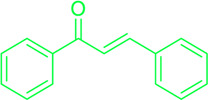 *Trans*-Chalcone	*Piper methysticum G.Forst. (Family: Piperaceae); Didymocarpus corchorifolius Wall. ex A.DC. (Family: Gesneriaceae); and Aniba riparia (Nees) Mez (Family: Lauraceae)*)	O=C (/C=C/C1 = CC = CC = C1)C2 = CC = CC = C2	Activity of TC in inflammatory cytokines induced joint stiffness and flexion pain	30, 60, and 120 mg/kg	Sprague−Dawley rats	Inhibition of IL-17, IL-1β, and IL-6 mRNA expression	Jabbar et al. (2024)
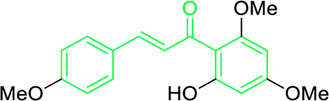 Flavokawain A	*Piper methysticum* G.Forst (Family: *Piperaceae*)	O=C(C1 = C(O)C=C(OC)C=C1OC)/C=C/C2 = CC = C(OC)C=C2	Antioxidant molecular mechanisms	2–30 μM (*in vitro*) 30 mg/kg (*in vivo*)	RAW264.7 cells and BALB/c female mice	Diminished the expression of TNF-α, IL-1β, and IL-6 Upregulation of IL-10 Impaired the LPS-triggered ROS production Inhibition of NF-κB (p65) pathway leads to attenuation of iNOS, COX-2, TNF-α, and IL-1β expression Triggered Nrf2 nuclear translocation leads to activating antioxidant proteins like HO-1, NQO-1, and γ-GCLC.	Yang et al. (2020)
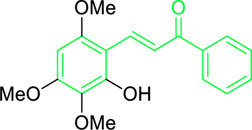 2-hydroxy-3,4,6-trimethoxychalcone	*Toussaintia orientalis* Verdc. (Family: *Annonaceae*)	O=C(C1 = CC = CC = C1)/C=C/C2 = C(OC)C=C(OC)C(OC) = C2O	Anti-inflammatory activity	30 μg/mL (Inhibition of COX-2)	Cell-free *in-vitro* assay and male Sprague-Dawley rats	Potent anti-inflammatory property by preventing the COX-2 expression	Nyandoro SS et al. (2012)
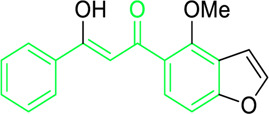 Pongamol	*Pongamia pinnata* (L.) Pierre (Family: *Fabaceae*)	O/C(C1 = CC = CC = C1) = C\C(C2 = CC = C(OC = C3)C3 = C2OC) = O	Anti-inflammatory property	IC_50_ = 72.2 μM (Inhibition of soy lipoxygenase-1) IC_50_ = 12.2 μg/mL (antioxidant activity)	Wistar rats	Reduced the edema by74% in the ear and 55% in the paw Inhibition of lipoxygenase-1	Rekha et al. (2020)

Prenylated chalcones are substituted with at least one lipophilic side chain of variable lengths, and numerous studies have demonstrated that, compared to parent chalcones, the prenyl motif has many benefits. Prenylation often increases affinity to the target site’s cell membrane. Prenylation also boosts lipophilicity, which improves target protein interaction and affinity for biological membranes. The prenylated chalcones are more extensively found in tissues and accumulate longer than their parent chalcones (Sychrová et al., 2022). Han et al. were the first to describe the extraction and characterization of a new prenylated chalcone known as 3″,3″-dimethylpyrano [ 3′,4′]2,4,2′-trihydroxychalcone from the *Artocarpus communis*. Several other chalcones, such as isobacachalcone, morachalcone A, gemichalcones B, and C were also extracted and characterized from *Artocarpus communis* for the first time. The newly isolated 3″,3″-dimethylpyrano [ 3′,4′]2,4,2′-trihydroxychalcone was characterized by NMR (^1^H, ^13^C NMR, ^1^H–^1^H COSY, ^1^H-^13^C HSQC, and ^1^H-^13^C HMBC) and mass (FABMS and HRFABMS) spectroscopic method. The isolated chalcones reduced NO generation from LPS-activated RAW264.7 mouse macrophage cells by reducing inducible nitric oxide synthase (iNOS: an inflammatory molecule that aids in synthesizing the NO and leads to inflammation). 3′′,3′′-dimethylpyrano [3′,4′]2,4,2′-trihydroxychalcone, morachalcone A, and gemichalcones B, displayed promising selectivity indices (3.1, 3.5, and 3.8, respectively), which show their ability to inhibit iNOS without causing cytotoxicity (Table 4) (Han et al., 2006). Some of the potential chalcones that cause inhibition of NO production are broussochalcone A, xanthohumol, cardamonin, isoliquiritigenin, isobacachalcone, morachalcone A, gemichalcones B, (Miranda et al., 2000b; Zhao et al., 2003; Ban et al., 2004; Lee et al., 2006; Lee et al., 2011). Lee et al. described that in lipopolysaccharide (LPS)-triggered microglial BV2 cells, xanthohumol reduced the stimulation of NF-κB signaling and decreased the inflammatory factors NO, IL-1β, and TNF-α (Figure 5). Furthermore, in LPS-induced BV2 cells, xanthohumol augmented the nuclear translocation of NRF2 and stabilized its cytoplasmic level, activating the intracellular production of glutathione (GSH), heme oxygenase-1 (HO-1), and NAD(P)H quinone oxidoreductase 1 (NQO1) (Table 4). These findings suggest that xanthohumol protects against LPS-triggered brain injury (Lee et al., 2011). Ban and colleagues also reported that cardamonin mediates anti-inflammatory function via suppressing the nuclear translocation of NF-κB (Ban et al., 2004).

Through blocking NF-κB signaling, cardamonin extracted from *Alpinia conchigera* reduces the expression of TNF-α, inductive NO synthase, and COX-2. Cardamomin decreased the NF-κB reporter gene generated by LPS in a dose-dependent way, having an IC_50_ results of 1.2 µM. Moreover, cardamomin had IC_50_ of 1.0 and 1.5 µM, respectively, and reduced the production of TNF and NO-triggered by LPS in a dose-dependent way (Table 4). Additionally, pretreatment with cardamomin (50 mg/kg) significantly diminished the mortality caused by LPS in C57BL/6 mice. Although cardamomin (50 mg/kg) pretreatment decreased the mice’s serum level of TNF-α, it had no significant consequence on the LPS-induced death of C57BL/6 mice after treatment (Lee et al., 2006).

Isoliquiritigenin inhibits the expression of inflammation regulatory molecule intercellular adhesion molecule-1 (ICAM-1) and vascular cell adhesion molecule-1 (VCAM-1) on the cell surface (Tanaka et al., 2001). Another class of chalcone named mallotophilippens C, D, and E downregulate the expression of inflammatory molecules COX-2, IL-6, and IL-1beta (Daikonya et al., 2004). 2′,3′-dihydroxy-4′,6′-dimethoxychalcone (DDC) was extracted from green *Perilla frutescens* var. Crispa f. viridis and characterized by NMR (^1^H, ^13^C, DEPT, TOCSY, HMQC, and HMBC) and high-resolution MS analysis. The authors also prepared DDC by using the Friedel-Crafts method from 2′-hydroxy-4′,6′-dimethoxyacetophenone, and *trans*-cinnamoyl chloride, and proton NMR analysis revealed that the isolated and synthetic DDC had the same chemical shifts and peak pattern. Through the initiation of the Nrf2-antioxidant response element (Nrf2-ARE) path, DDC promotes an increase in the expression of antioxidant enzymes γ-glutamylcysteine synthetase, NQO1, and HO-1 (Table 4). The authors claim that by increasing the expression of many antioxidant proteins and inhibiting the generation of intracellular ROS, DDC enhanced cellular resistance to 6-OHDA-persuaded cytotoxic activity (Izumi et al., 2012). According to another study, Hesperidin methyl chalcone also demonstrates antioxidant properties, which help heal inflammatory colitis. Increases in ferric reducing antioxidant power (FRAP), 2.2′-azino-bis (3-ethylbenzothiazoline-6-sulfonic acid (ABTS) scavenging, and GSH levels were indicative of an enhancement in total antioxidant capability following hesperidin methyl chalcone treatment. Hesperidin methyl chalcone suppresses the expression of multiple pro-inflammatory cytokines in the colon, such as TNF α, IL-1β, IL-6, and IL-33 (Table 4). The possibility that this substance inhibits the colon’s NF-κB pathway activity has also been explored (Guazelli et al., 2021).

In another study, an inflammation-induced mice model was administrated with varying dosages of hesperidin methyl chalcone (10, 30, or 100 mg/kg) to investigate the involvement of hesperidin methyl chalcone in zymosan-mediated inflammation (Table 4). The macrophage cell (RAW 264.7) was also employed to evaluate oxidative stress concurrently. Analysis of the antioxidative and anti-inflammatory tests revealed that the activation of macrophage NF-κB and the generation of ROS were reduced by hesperidin methyl chalcone treatment. Nevertheless, hesperidin methyl chalcone significantly decreased knee joint edema, mechanical hypersensitivity, and leukocyte recruitment while impairing pro-inflammatory cytokine expression (Rasquel-Oliveira et al., 2020).

Hesperidin methyl chalcone has also been exhibited to trigger the Nrf2 signaling pathway to protect against oxidative stress. Recently, analysis evaluated the function of hesperidin methyl chalcone in controlling the expression of cytokines to explore this situation further. Diclofenac (200 mg/kg) was provided orally to develop an inflammatory mouse model, which was subsequently treated with 0.3–3 mg/kg of hesperidin methyl chalcone. Subsequently, several biological parameters were examined, including kidney edema, histopathology, urine neutrophil gelatinase-associated lipocalin (NGAL), and Nrf2 mRNA expression. As a result, decreased urea levels and creatinine and lipid peroxidation, have been linked to the downregulation of IL-33, IL-6, and interferon-γ (IFN-γ) (Table 4). Not only may hesperidin methyl chalcone cause the Nrf2 pathways to be downregulated, but it also decreases the expression of downstream proteins, including HO-1, NQO1, and Kelch-like ECH-related protein 1 (Keap1) (Bussmann et al., 2022).

Another particular condition that can target the body’s immune system is autoimmune disease. Numerous immune cells and cytokines may contribute to developing these kinds of illnesses. *Trans* chalcone, a naturally occurring chalcone, was given orally to rats with joint tissue stiffness, an autoimmune disease caused by complete Freund’s adjuvant (CFA) to treat this disorder. *Trans* chalcone dosages were examined in increasing order of administration: 30 mg/kg, 60 mg/kg, and 120 mg/kg at the end. ELISA and RT-PCR were employed to measure the expression levels of IL-17, TNF-α, iNOS, and COX-2 to assess the outcome of *trans* chalcone efficacy (Table 4). Consequently, these pro-inflammatory cytokines decrease, demonstrating the efficaciousness of trans chalcone in reducing ameliorating joint stiffness (Jabbar et al., 2024).

Yang et al. demonstrated that flavokawain A, a central metabolite of chalcones (0.46%) isolated from kava extracts, enhanced the expression of antioxidant proteins in primary splenocytes (Yang et al., 2020). *In vitro* analysis demonstrated that nontoxic dosages of flavokawain A (2–30 μM) prominently repressed the release of proinflammatory cytokines (TNF-α, IL-1β, and IL-6) while stimulating the production of interleukin-10, an anti-inflammatory cytokine (Table 4). *In vitro* results are supported by *ex vivo* results from primary splenocytes derived from oral flavokawain A-preadministered BALB/c mice, demonstrating that flavokawain A substantially repressed the secretion of proinflammatory cytokines (TNF-α, IL-1β, and IL-6) in cells stimulated by LPS, Concanavalin A, or control. Significant reductions in the ratios of pro- and anti-inflammatory cytokines (IL-6/IL-10; TNF-α/IL-10) indicate the potent anti-inflammatory characteristics of flavokawain A. Moreover, in BALB/c mice given cholecystokinin (CCK) 8 to induce experimental pancreatitis, pre-treatment with flavokawain A led to decreased blood lipase levels. Another study demonstrated the anti-inflammatory role of isolated metabolites, including 2-hydroxy-3,4,6-trimethoxychalcone from the *Toussaintia orientalis* Verdc leaf extracts. Among these isolated metabolites, 2-hydroxy-3,4,6-trimethoxychalcone showed anti-inflammatory attributes in the inflammation-inducing enzyme COX-2 (Table 4) (Nyandoro SS et al., 2012). Pongamol is a β-hydroxybenzofuranchalcone with double bonds with -OH, -OMe, and ethylene moieties. It can be found in pure enol form in *Tephrosia purpurea* roots and *Pongamia pinnata* (karanjin) seeds. According to the solid-state structure of pongomal, the mean plane of the phenyl group is twisted by 27.9° from the mean plane of the β-hydroxy chalcone, which is twisted by 34.8° from the mean plane of the benzofuran moiety (Parmar et al., 1989). Recently, a study was conducted where pongamol, isolated from the *Pongamia pinnata,* interestingly showed anti-inflammatory activity and antioxidant activities, having an IC_50_ results of 12.2 μg/mL (Table 4). In this study Wistar rats were used to induce edema in the paw and ear by utilising Carrageenan and xylene, which get reduced after administrating the dihydropongamol with a concentration of 50 ppm (Rekha et al., 2020).

Several chalcones exhibit significant anti-inflammatory and antioxidant properties, including xanthohumol, isoliquiritigenin, and hesperidin methyl chalcone. The SAR analysis confirmed that the chalcones having –OH in the B-ring and –OCH_3_ in the *para* position of the A-ring were often potent antioxidant properties (Sivakumar et al., 2011). Furthermore, chalcones’ anti-inflammatory activity is enhanced by a -OH at *p*-position in ring-B. The anti-inflammatory action is further enhanced by substituting -OH and -OCH_3_ on both rings, suppressing NO generation (Ur Rashid et al., 2019). The existence of -OH and -OCH_3_ moieties in chalcones also represses the activity of adenosine receptors-A1, A2A and shows antioxidant and anti-inflammatory properties. They achieve this by inhibiting pro-inflammatory cytokines, suppressing enzymes like COX-2, and activating the Nrf2 pathway to boost antioxidant defenses. Studies in various models, including LPS-induced microglial cells and inflammation-induced mice, demonstrate these metabolites’ potential to diminish inflammation, oxidative stress, and related conditions such as autoimmune diseases and joint stiffness. Flavokawain A, another chalcone, also shows strong anti-inflammatory effects, enhancing antioxidant protein expression while reducing pro-inflammatory cytokines.

### 4.4 Chalcones as modifiers of enzyme actions in a biological system

Chalcones modify various enzyme actions and reshape the functions and metabolic activities within the biological body. Chalcone modifies the functions of almost all classes of enzymes, including oxidoreductases, cyclooxygenase and 5-lipoxygenase, aldose reductase, thioredoxin reductase, monoamine oxidase, proteases, esterase, etc., (Zhou, 2015). By modifying these enzyme activities, chalcone compounds can potentially change various metabolic events, biosynthesis of substances, disease development and progression, cancer, and many other pathological conditions. Chalcones have been found to modify mitochondrial enzymes, decrease cholesterol and melanin synthesis, modify estrogen biosynthesis, and many more. As the mechanism of modifying the enzyme actions, chalcone compound acts like competitive inhibitors and causes reversible inhibition of enzymes. Inhibiting some of the enzyme actions leads to the antidiabetic activity of the chalcone compounds, which we will discuss in the coming sections.

Many chalcones including isoliquiritigenin, 4-Hydroxychalcone, butein, 2,4,2′,4′-tetrahydroxy-3-(3-methyl-2-butenyl)-chalcone (TMBC), and 2′,4′,4-trihydroxychalcone etc., possess tyrosinase inhibitory activity (Nerya et al., 2003; 2004a; Zhang et al., 2009; Singh et al., 2020). Zhang and his group isolated TMBC, from the *Morus nigra* stem and characterized by NMR (^1^H and ^13^C NMR) and EIMS mass spectroscopic method (Zhang et al., 2009). It was found that TMBC reduced tyrosinase action and total melanin content of B16 cells without causing appreciable cytotoxicity. Furthermore, TMBC (IC_50_ = 0.95 ± 0.04 µM) was found to have around 25 times the potency of a recognised tyrosinase inhibitor kojic acid (IC_50_ = 24.88 ± 1.13 µM) (Table 5).

**TABLE 5:** Plant source, doses, and enzyme modifiers actions of different natural chalcones.

Name and structure of chalcone	Plant source	Smiles	Activity	Dose of tested drug	Target	Mechanism of action	References
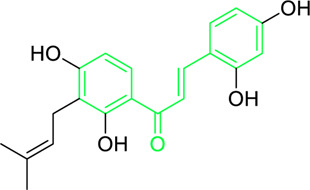 2,4,2′,4′-Tetrahydroxy-3-(3-methyl-2-butenyl)-chalcone (TMBC)	*Morus nigra* L. (Family: *Moraceae*)	O=C (/C=C/C1 = C(O)C=C(O)C=C1)C2 = C(O)C(C/C=C(C)\C) = C(O)C=C2	Inhibit both cellular tyrosinase action in tumor cells	IC_50_ = 0.95 ± 0.04 µM	B16 (melanoma cell)	Direct inhibition of tyrosinase activity	Zhang et al. (2009)
Xanthohumol 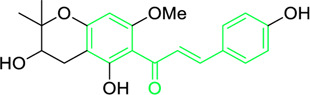 Xanthohumol B 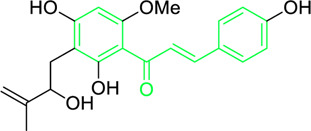 Xanthohumol D	*Humulus lupulus* L. (Family: *Cannabaceae*)	O = C (/C=C/C1 = C(O)C=C(O)C=C1)C2 = C(OC)C=C(O)C(C/C=C(C)\C) = C2O O=C (/C=C/C1 = CC = C(O)C=C1)C2 = C(O)C3 = C(OC(C) (C)C(O)C3)C=C2OC O=C (/C=C/C1 = CC = C(O)C=C1)C2 = C(O)C(CC(O)C(C) = C) = C(O)C=C2OC	Induces the quinone reductase (QR) activity	Tested concentration of drug is 20 μM	Hepa 1c1c7 (mouse hepatoma cell line)	Not Known	Yu et al. (2014)
Isoliquiritigenin	*Dipteryx odorata*. (Aubl.) Forsyth f. (Family: *Fabaceae*)	OC(C=C1O) = CC = C1C(/C=C/C2 = CC = C(O)C=C2) = O	Induces quinine reductase activity	Tested concentration of drug is 2–30 μM	Hepa 1c1c7 cells	Not known	Cuendet et al. (2006)
Isoliquiritigenin 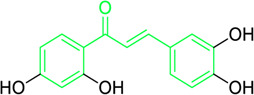 Butein 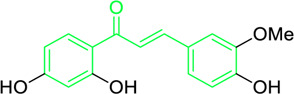 Homobutein	Not reported	OC(C=C1O) = CC = C1C(/C=C/C2 = CC = C(O)C=C2) = O OC1 = CC = C(C(/C=C/C2 = CC = C(O)C(O) = C2) = O)C(O) = C1 OC1 = CC = C(C(/C=C/C2 = CC = C(O)C(OC) = C2) = O)C(O) = C1	Anti-inflammatory and anti-tumor activity	IC_50_ = 60–190 µM (with inhibition of HDAC) IC_50_ = 8–41 µM (TNFα-triggered NF-κB activation-inhibition)	K562 cells	Inhibit the expression of Histone deacetylase enzymes Attenuated TNFα-triggered NF-κB activation	ORLIKOVA et al. (2012)
Morachalcone A 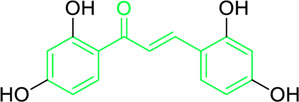 2,4,2′,4′-tetrahydroxychalcone	Twigs of *Morus alba* L. (Family: *Moraceae*)	O=C (/C=C/C1 = CC = C(O)C=C1O)C2 = C(O)C(C/C=C(C)\C) = C(O)C=C2 O=C (/C=C/C1 = CC = C(O)C=C1O)C2 = C(O)C=C(O)C=C2	Tyrosinase Inhibition metabolites activity characterization	IC_50_ = 0.07 ± 0.02 µM (2,4,2′,4′-tetrahydroxychalcone) IC_50_ = 0.08 ± 0.02 µM (Morachalcone A)	Not Reported	Inhibition of tyrosinase better than positive control kojic acid	Zhang et al. (2016)
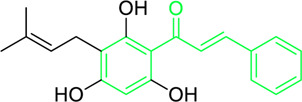 2′,4′,6′-trihydroxy-3′-prenylchalcone	*Helichrysum teretifolium* (L.) Sweet (Family: *Asteraceae*)	O=C (/C=C/C1 = CC = CC = C1)C2 = C(O)C(C/C=C(C)/C) = C(O)C=C2O	Oxidative Stress as well as Skin Aging-associated enzymes inhibition	4,529.01 ± 2.44; 4,170.66 ± 6.72 μM TE/g	Not Reported	Modulated biological activity than other isolated metabolites including isoxanthohumol from the same source	Popoola et al. (2015)
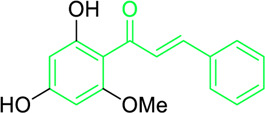 2′4′-dihydroxy-6′-methoxy-chalcone	Leaves and stems from *Loranthus acutifolius* Ruiz & Pav. (Family: *Loranthaceae*)	OC1 = CC(O) = C(C(/C=C/C2 = CC = CC = C2) = O)C(OC) = C1	Melanin production and tyrosinase inhibition activity	IC_50_ = 5.7 ± 0.02 μM (For tyrosinase inhibition)	B16-F10 cells	Melanin production and tyrosinase activity inhibition	Apaza Ticona et al. (2021)
Cardamonin	Not reported	OC1 = C(C(/C=C/C2 = CC = CC = C2) = O)C(OC) = CC(O) = C1	Nrf2-mediated antioxidant enzymes biosynthesis in colon cancer cells	50 µM	Caco-2 cells	Not elevated gene expression of TrxR1 and glutathione peroxidase 2 (GPx2) Attenuation of O-phosphoseryl-tRNA (Sec) kinase (PSTK) associated with the translation of selenoprotein mRNAs This metabolite interferes biosynthesis of Nrf2-regulated selenoenzymes	De Spirt et al. (2016)

Tyrosinase is an important enzyme that works on substrates like l-tyrosine and l-DOPA to catecholates, benzoquinones etc., and act as a critical role in melanogenesis and skin pigmentation (Olivares and Solano, 2009). Expression of tyrosinase is also reported to increase during tumorigenesis, and it may accelerate tumor growth (Boyle et al., 2002). Development of effective tyrosinase inhibitors will therefore be useful in managing skin pigmentation and cosmetic industries and more. Chalcone compounds also cause inhibition of ornithine decarboxylase (Luyengi et al., 1994), aromatase, 17β-hydroxysteroid dehydrogenase (Le Bail et al., 2001) and 5α-reductase enzyme. These enzymes act in the biosynthetic pathway of sex steroids: aromatase in estrogen synthesis, 5α-reductase, and 17β-hydroxysteroid dehydrogenase in androgen synthesis. Chalcones isolated from *Broussonetia papyrifera* cause inhibition of aromatase enzyme, reducing estrogen biosynthesis in human placental microsomal preparation (Le Bail et al., 2001). Ornithine decarboxylase and aromatase activity increase during various types of cancer, mediate some molecular events in cancer pathogenesis, and thus can be a probable therapeutic target (Chumsri et al., 2011; Kim et al., 2017). Aromatase and ornithine decarboxylase inhibitors were very effective in treating cancer (Kim et al., 2017; Kharb et al., 2020). Chalcones also inhibit the enzyme cytochrome P450 1A (CYP1A), whose activity is essential for the metabolic initiation of polycyclic aromatic hydrocarbons and cause chemoprevention (Forejtníková et al., 2005). Essential functions in the redox control of multiple cellular signaling pathways comprised in proliferation and cell growth are played by thioredoxin reductase (TrxR: an essential protein that regulates the redox environment in cells) (Mahmood et al., 2013; Lu and Holmgren, 2014). Since many cancers overexpress the thioredoxin system, which confers drug resistance to cancer chemotherapy, there has been growing evidence in recent years that TrxR is a potential target for developing new anti-cancer drugs (Kim et al., 2005). Hatfield et al. provided proof of the physiological importance of TrxR for the progression of tumors, demonstrating that TrxR knockdown decreases the proliferation and DNA replication of melanoma cells and the development of tumors (Yoo et al., 2007). Xanthohumol, xanthohumol B and D, and xanthohmol analogs isolated from *Humulus lupulus* L. cause potent inhibition of the enzyme aromatase, quinone reductase, and thioredoxin reductase (Monteiro et al., 2007; Yu et al., 2014). An investigation into the potential effects of xanthohumol on the control of estrogen synthesis was conducted on the aromatase-expressing breast cancer cell Sk-Br-3 (Monteiro et al., 2007). According to the investigators, the aromatase activity in Sk-Br-3 was observed to be reduced by incubation with different dosages of xanthohumol (IC_50_ = 3.2 µM).

Further research on the relationship between the inhibition of cellular proliferation and aromatase inhibition showed that xanthohumol treatment on Sk-Br-3 cells for 72 h decreased protein synthesis dose-dependent (IC_50_ = 7.1 µM). Of all the substances tested, Sk-Br-3 cells treated with xanthohumol showed the greatest reduction in DNA synthesis (IC_50_ = 0.52 µM). The authors’ explanation of xanthohumol included reduction of the aromatase enzyme and a decrease in the rate of estrogen production (Monteiro et al., 2007).

Yu et al. isolated a novel prenylated chalcone xanthohumol, and the first new natural bichalcone humulusol having a prenyl substitution and methylene connector along with six known chalcones from *Hmulus lupulus* and evaluated their quinone reductase (QR) induction properties using hepa 1c1c7 cells (Table 5) (Yu et al., 2014). Newly isolated chalcones were characterized by HR-ESI-MS, and spectroscopic methods (^1^H NMR, ^13^C NMR, and HMBC). All the isolated chalcones demonstrated significant electrophilic ability, good solubility, and good QR induction activity. SAR studies on the isolated chalcones indicated that the QR induction activity is attributed to the *trans* double bond of the chalcones.

Another well-studied chalcone named isoliquiritigenin reported quinine reductase activity at micromolar concentration and was found helpful in cancer chemoprevention (Cuendet et al., 2006). It was found that isoliquiritigenin (2–30 μM), a monofunctional inducer with a maximum of 7 times induction at the highest tested concentration, could activate quinone reductase in Hepa 1c1c7 cells of the wild type, hence reducing the risk of cancer (Table 5). Furthermore, treatment with isoliquiritigenin (7.5–30 μM) did not exhibit any cytotoxicity and dramatically increased luciferase expression through interaction with ARE in a dose-dependent path.

A study explored the role of four major chalcones, including butein, isoliquiritigenin, glycoside marein, and homobutein out of 21 isolated natural chalcones for inhibiting the expression of HDACs, as HDACs regulate the NF-κB transcription factor for promoting the inflammation-mediated cancer progression. These four metabolites showed the IC_50_ = 60–190 µM as well as inhibit the TNFα-triggered NF-κB activation with IC_50_ value 8–41 µM. Interestingly, soliquiritigenin, butein, and homobutein showed an inhibitory role against the expression of total HDAC activities of classes I, II, and IV and TNFα-triggered NF-κB activity (Table 5) (ORLIKOVA et al., 2012). Zhang et al. also reported the inhibitory role of 2,4,2′,4′-tetrahydroxychalcone, and morachalcone A, isolated from the *Morus alba* L. towards tyrosinase. These metabolites disclosed a stronger repressive activity against tyrosinase than positive control kojic acid. 2,4,2′,4′-tetrahydroxychalcone showed IC_50_ = 0.07 ± 0.02 µM, and morachalcone A showed IC_50_ = 0.08 ± 0.02 µM (Table 5) (Zhang et al., 2016).

Furthermore, to study the reduction of oxidative stress and skin aging-associated enzymes, 2′,4′,6′-trihydroxy-3′-prenylchalcone extracted from the *Helichrysum teretifolium* methanolic extract. This metabolite showed modulated biological activity than other isolated metabolites, including isoxanthohumol from the same source. For instance, it exhibited some of the highest TEAC results (4,529.01 ± 2.44; 4,170.66 ± 6.72) μM TE/g (Table 5) (Popoola et al., 2015). Nevertheless, 2′4′-dihydroxy-6′-methoxy-chalcone isolated from the *Loranthus acutifolius* leaves and stems was also reported as a potential inhibitor in melanin production and tyrosinase activity. To delve into this activity, this metabolite was tested on B16-F10 cells, where it determined the IC_50_ value with 1.6 ± 0.03 μM. The prospect of tyrosinase inhibition by this same metabolite was also reported with IC_50_ = 5.7 ± 0.02 μM (Table 5) (Apaza Ticona et al., 2021). Interestingly, cardamonin a natural chalcone, was also reported as antioxidant activity by Nrf2 pathway-mediated enzymes (selenoenzymes) biosynthesis activation in 50 µM concentration in Caco-2 cell line (Table 5) (De Spirt et al., 2016).

Chalcones, such as isoliquiritigenin, TMBC, and xanthohumol, exhibit strong inhibitory effects on enzymes like tyrosinase, aromatase, and quinone reductase. These activities make them effective against skin pigmentation, cancer cell proliferation, and oxidative stress. The SAR studies about the tyrosinase inhibition potency indicate that the position of the -OH on A and B rings, having a substantial preference for a 4-substituted B ring, rather than a substituted A ring, is the most important factor in their efficacy; neither the number of -OH nor the incidence of a catechol moiety on ring B interrelated with enhancing tyrosinase reduction potency (Nerya et al., 2004b). TMBC, for example, is 25 folds more effective than kojic acid in preventing tyrosinase, a key enzyme in melanogenesis. Xanthohumol shows significant potential in reducing estrogen synthesis in breast cancer cells. Chalcones like butein and isoliquiritigenin also inhibit HDAC enzymes, further demonstrating their potential in cancer prevention and therapy.

### 4.5 Antiobesity and cardioprotective activity of naturally occurring chalcones

Cardiovascular diseases (CVDs) are among the foremost causes of human mortality globally, and CVDs caused 20.5 million deaths in 2021 alone, or over one-third of all fatalities worldwide, and are predicted to impact 23.3 million humans by year 2030 (Lindstrom M et al., 2022). Ischaemic heart disease caused by cholesterol deposition and the development of atherosclerotic plaque inside the coronary arteries has become one of the leading causes of death globally. Cardiovascular health is determined by numerous aspects, such as the cholesterol and triglyceride levels in the body, obesity, atherosclerosis of the blood vessels, and blood pressure, blood parameters, as well as the musculature of the heart, and all the factors regulating the myocardial activity. Variations in gene sequences may also be caused by chronic pathological diseases like cardiovascular disease. In this regard, a good number of studies found a link between the advancement of CVD and single-nucleotide polymorphisms (SNPs: variation in one building block of the genetic sequence) in inflammatory genes. Numerous SNPs linked to inflammatory molecules, for example, C-reactive protein (CRP), IL-10, IL-1, TNF-α, IL-6, and transforming growth factor-β (TGF-β), have been associated with the development of CVD (Deb et al., 2022). Chalcone compounds working on any of those mentioned above and also target the lipoprotein lipase (LPL), pancreatic lipase (PL), angiotensin-converting enzyme (ACE), cholesterol acyltransferase (ACAT), diacylglycerol acyltransferase (DGAT), cholesteryl ester transfer protein (CETP), thromboxane A2 (TXA2), calcium-potassium channel, and thromboxane B2 (TXB2), COX-1 can affect the cardiovascular health (Mahapatra and Bharti, 2016). Chalcones can be used as potential cardiovascular agents to treat and manage many cardiovascular disease conditions like the management of hypertension, prevention of atherosclerosis, impeding ischemia-induced myocardial infarction, and improving overall myocardial health (Mahapatra and Bharti, 2016; Khan et al., 2021; Maisto et al., 2023). Treatment with many chalcone compounds like 4-hydroxyderricin, xanthoangelol, and isoliquiritigenin reduced the ischemia-induced myocardial infarction and protected the antioxidant system (Ogawa et al., 2005; AN et al., 2006).

**FIGURE 6:**
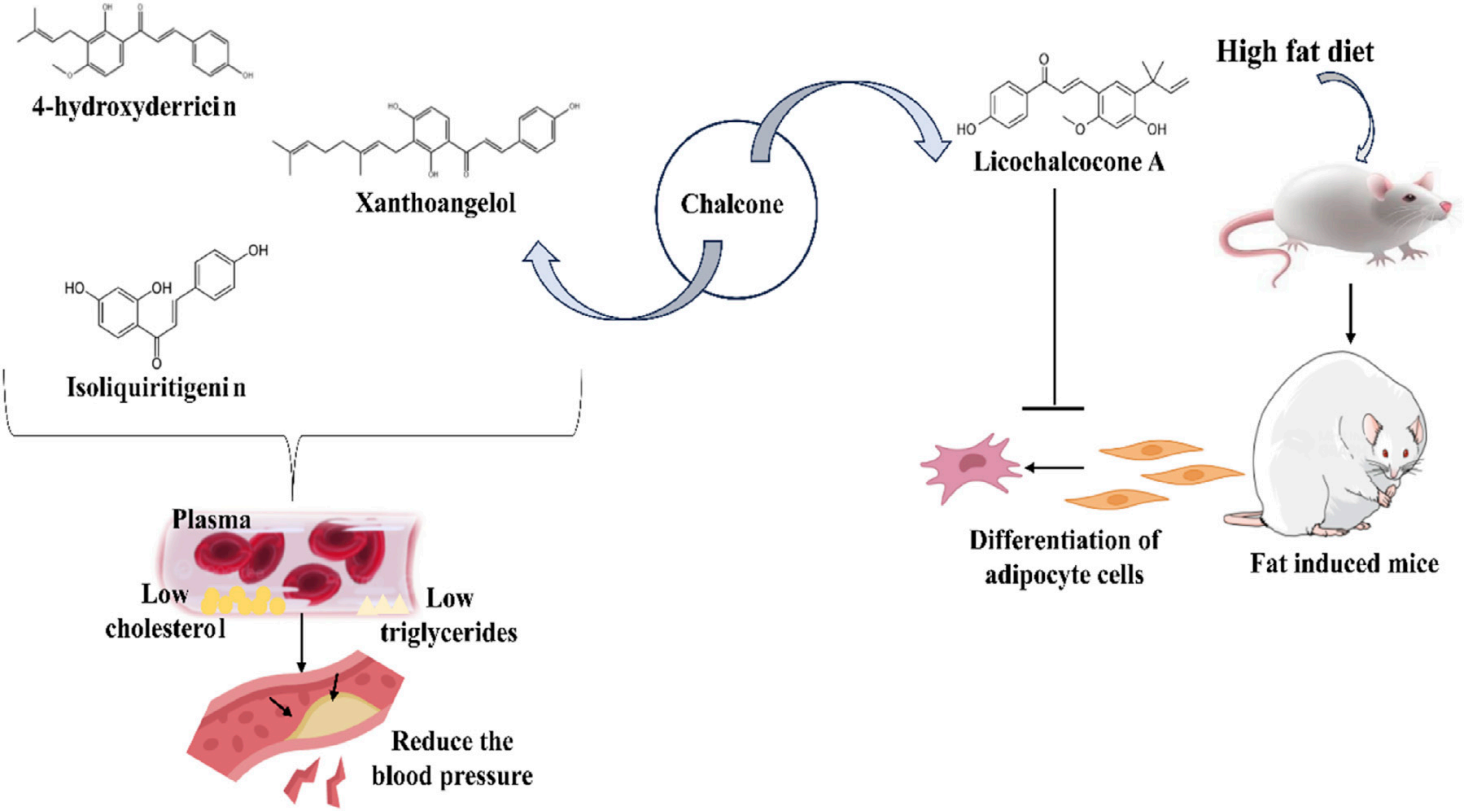
Different naturally isolated chalcones in maintaining blood pressure and fat deposition. 4-hydroxyderricin, xanthoangelol, and isoliquiritigenin reduce the deposition of cholesterol and triglycerides in arteries to prevent high blood pressure. In contrast, licocholane A can also inhibit the differentiation of the adipocyte cells in the HFD-induced fatty mice model.

Chalcones also reduce blood pressure effectively and prevent atherosclerosis (Avila-Villarreal et al., 2013; Chen et al., 2020) by reducing the plasma cholesterol, low-density lipoprotein (LDL), and triglycerides levels (Ogawa et al., 2007; Birari et al., 2011). A 3-prenylated chalcone with a C5-isoprenoid unit at the 3-position is called 4-hydroxyderricin. In stroke-prone spontaneously hypertensive rats (SHRSP), Ogawa and colleagues extracted 4-hydroxyderricin from the *Angelica keiskei* stems and examined the dietetic results of 4-hydroxyderricin on lipid metabolism and blood pressure (Ogawa et al., 2005). The authors attributed that 4-hydroxyderricin (Figure 6) inhibited the rise in systolic blood pressure, lowered the levels of serum very-low-density lipoprotein (VLDL), and reduced hepatic triglyceride in SHRSP (Table 6). A study looking at the hepatic mRNA expression of lipid metabolism-related proteins suggested that the drop in serum VLDL levels could be caused by a significant decrease in microsomal triglyceride transfer protein, and the drop in hepatic triglyceride content could be triggered via significant decreases in fatty acid synthase and adipocyte determination in addition to differentiation factor 1.

**TABLE 6:** Plant source, doses, and cardioprotective roles of different natural chalcones.

Name and structure of chalcone	Plant source	Smiles	Activity	Dose of drug	Target model	Mechanism of action	References
4-hydroxyderricin	*Angelica keiskei*. (Miq.) Koidz. (Family: *Apiaceae*)	OC1 = C(C(/C=C/C2 = CC = C(O)C=C2) = O)C=CC(OC) = C1C/C=C(C)/C	Cardioprotective Reduction of serum VLDL and hepatic triglyceride	Diet containing 0.07% 4-hydroxyderricin	Hypertensive rats	Reduced expression of mRNA is responsible for lipid metabolism such as microsomal triglyceride with downregulation of fatty acid synthase	Ogawa et al. (2005)
Xanthoangelol	*Angelica keiskei*. (Miq.) Koidz. (Family: *Apiaceae*)	OC1 = C(C/C=C(C)/CC/C=C(C)/C)C(O) = C(C(/C=C/C2 = CC = C(O)C=C2) = O)C=C1	Cardioprotective Reduced serum LDL, triglyceride contents, and total cholesterol in liver tissue	Diet containing 0.10% xanthoangelol	Hypertensive rats	Increased hepatic PPARα mRNA expression as well as hepatic ACO and ACS mRNA.	Ogawa et al. (2005)
Isoliquiritigenin	*Glycyrrhiza glabra. *L. (Family: *Fabaceae*)	OC(C=C1O) = CC = C1C(/C=C/C2 = CC = C(O)C=C2) = O	Cardioprotective Attenuates severity of myocardial reperfusion	20 mg/kg body weight	Rats with myocardial ischemia-reperfusion	Upregulation of metallothionein (MT) expression	AN et al. (2006)
Isoliquiritigenin	*Glycyrrhiza glabra*.L. (Family: *Fabaceae*)	OC(C=C1O) = CC = C1C(/C=C/C2 = CC = C(O)C=C2) = O	Antiobesity and lipid-lowering effects	IC_50_ for pancreatic lipase inhibition is 7.3 µM *In vivo* treatment dose is 30 mg/kg body weight/day	Male SD rats fed	Inhibition of pancreatic lipase (PL)	Birari et al. (2011)
Licochalcone A	*Glycyrrhiza sp*.	OC1 = C(C(C) (C)C=C)C=C (/C=C/C(C2 = CC = C(O)C=C2) = O)C(OC) = C1	Suppresses adipocyte differentiation	Treatment given are 5 and 10 mg/kg body weight	HFD-induced ICR mice	Downregulation of peroxisome proliferator-triggered receptor γ, fatty acid synthase, and other enzymes like stearoyl-CoA desaturase 1 to reduce obesity	Quan et al. (2012)
Licochalcone A	*Glycyrrhiza uralensis*.Fisch. ex DC. (Family: *Fabaceae*)	OC1 = C(C(C) (C)C=C)C=C (/C=C/C(C2 = CC = C(O)C=C2) = O)C(OC) = C1	Decrease the level of plasma cholesterol	10 mg/kg body weight	HFD-induced C57BL/6 mice	Instigates the expression of uncoupling protein 1 (UCP1) for ant-obesity in 3T3-L1 adipocyte cells	Lee et al. (2018)
Flavokawains A Flavokawains B 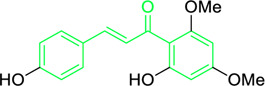 Flavokawains C	*Kaempferia angustifolia* Roscoe (Family: *Zingiberaceae*)	O=C(C1 = C(O)C=C(OC)C=C1OC)/C=C/C2 = CC = C(OC)C=C2 O=C(C1 = C(O)C=C(OC)C=C1OC)/C=C/C2 = CC = CC = C2 O=C(C1 = C(O)C=C(OC)C=C1OC)/C=C/C2 = CC = C(O)C=C2	Cardioprotective	10 μg/mL	3T3-L1 murine model	Inhibited triglyceride accumulation	Hanif et al. (2022)
Licochalcone A	*Glycyrrhiza uralensis* Fisch. ex DC. (Family: *Fabaceae*)	OC1 = C(C(C) (C)C=C)C=C (/C=C/C(C2 = CC = C(O)C=C2) = O)C(OC) = C1	Potential activity of ameliorating obesity	1.5–12 μM (in HepG2 cells)	HepG2 hepatocytes Male C57BL/6 mice	Triggered the sirt-1/AMPK path Downregulate the fatty acid chain synthesis Enhance lipolysis and β-oxidation in liver cells	Liou et al. (2019)
Xanthohumol	*Humulus lupulus* L. (Family: *Cannabaceae*)	O=C (/C=C/C1 = C(O)C=C(O)C=C1)C2 = C(OC)C=C(O)C(C/C=C(C)\C) = C2O	Regulatory activity of lipid metabolism	10 and 20 μM	HepG2 and Huh7 cells Zebrafish model	Attenuated the expression of ANGPTL3 mRNA and protein	Gao et al. (2024)

The same authors also extracted xanthoangelol (Figure 6) from *A. keiskei*, and inspected the result of dietetic xanthoangelol on blood pressure and lipid metabolism in SHRSP (Ogawa et al., 2007). It was noted that the xanthoangelol increased the expression of LDL-receptor (LDL-R) mRNA in the liver, and it caused a decline in blood LDL. In addition to lowering liver weight in SHRSP, xanthoangelol (0.10%) also caused a decline in the hepatic cholesterol pool via increasing fecal cholesterol excretion (Table 6). Furthermore, xanthoangelol significantly enhanced the expression of peroxisome proliferator activated receptor-α (PPAR-α) in the liver in conjunction with increases in the expression of Acyl-coenzyme A oxidase (ACO) and Acyl-CoA synthetase (ACS) in the liver, suggesting an acceleration of fatty acid β-oxidation.

In an *in vivo* analysis of MI/R rats, An and his group observed that isoliquiritigenin (Figure 6) prominently decreased the myocardial infarct size and avoided arrhythmias caused by reperfusion (AN et al., 2006). Lactate dehydrogenase (LDH) and creatinine phosphokinase (CPK) activity in the isoliquiritigenin (20 mg/kg) group were 38.4% and 51.3% lower, respectively, than the vehicle group (Table 6). In isoliquiritigenin-treated groups, there was an increase in metallothionein (MT) synthesis along with enhanced JAK 2/STAT 3 phosphorylation, but not in COX-2 or iNOS production. AG490 can notably reduce isoliquiritigenin-induced cardioprotection and stop MT expression from rising and JAK 2/STAT 3 phosphorylation from happening.

Birari and colleagues extracted twelve flavonoids, including the chalcone isoliquiritigenin from the *Glycyrrhiza glabra* roots and examined the pancreatic lipase (PL) repressive action *in vitro* (Birari et al., 2011). Isoliquiritigenin among the evaluated metabolites exhibited a substantial inhibitory result towards PL, with an IC_50_ = 7.3 µM (Table 6). The antiobesity and lipid-lowering outcomes of chalcone isoliquiritigenin in male SD mouse fed a high-fat diet (HFD) were observed. These rats’ body weight enhanced by only 23.2 ± 3.6 g when supplemented with isoliquiritigenin, whereas the body weight of the HFD control group enhanced by 64.2 ± 0.5 g, suggesting the significance of the chalcone moiety as a cause for stopping obesity. Moreover, isoliquiritigenin decreased plasma total cholesterol to 84.6 ± 1.4 mg/dL and plasma total triglycerides to 128.8 ± 6.0 mg/dL.

The mechanistic study performed on antihypertensive action revealed reduction of ACE to blocking of calcium channel and *β* receptors by various chalcone compounds (Kumar et al., 2015; Goshain and Ahmed, 2019). Licochalcone A shows potent antiobesity property in HFD-fed ICR mice and suppresses adipocyte differentiation. Treatment with licochalcone A lowers gain in body weight and diminishes plasma levels of triglyceride, cholesterol, and nonesterified fatty acid. When compared to the HFD control mouse, the total fat volume of the mice given 5 and 10 mg/kg of licochalcone A reduced by 60% and 80%, respectively (Table 6) (Quan et al., 2012). Additionally, in a mouse model, the group provided 10 mg/kg licochalcone A had triglyceride, cholesterol, and no esterified fatty acid levels that were 14.0%, 48.2%, 58.9%, and 73.5% of body weight, respectively, lower than the control group. In 3T3-L1 adipocyte cells, a mechanistic study showed downregulation of sterol regulatory element-binding protein 1c, fatty acid synthase, glycerol-3-phosphate acyltransferase, stearoyl-CoA desaturase 1, and peroxisome proliferator-activated receptor γ. Additionally, it induced the expression of uncoupling protein 1 (UCP1) (Quan et al., 2012). Lee et al. also explored the antiobesity action of licochalcone A (Lee et al., 2018). Experimental results indicated that licochalcone A treatment causes browning of the subcutaneous adipocytes in the HFD-induced obesity mouse through promoting the expression of essential markers specific to brown adipocytes, which in turn drives the thermogenic gene program (Table 6). The authors attributed that licochalcone A may increase fat oxidation, lipolysis, and thermogenesis while decreasing lipogenesis.

Recently it was described that at 10 μg/mL concentration in a murine model of pre-adipocytes, a natural chalcone flavokawains A extracted from the rhizomes of the Indonesian terrestrial plant *Kaempferia angustifolia* exhibits antiobesity activity and reduced cytotoxic effect (EC_50_ = 39.2 µM) (Table 6) (Hanif et al., 2022). The authors also synthesized flavokawains A, flavokawains B, and flavokawains C to determine the activity of natural flavokawains A and investigated the results of electron-donating moieties of the B ring. Synthetic flavokawains A’s EC_50_ was 64.4 µM, comparable to natural flavokawains A’s value. It was also found that flavokawains C was more selective than the number flavokawains A, the authors attributed that the B ring’s reactivity has been decreased by a *p*-hydroxy (electron-donating) group and the antiobesity effect may be ascribed to the electron-donating moiety on the B ring.

A study revealed the potential antiobesity and protective role against nonalcoholic fatty liver diseases of licochalcone A, derived from *Glycyrrhiza uralensis*. This isolated metabolite was tested on HFD-mediated obesity-induced male C57BL/6 mice. Simultaneously, the oleic acid-induced fatty liver model was generated in the HepG2 cell line. After administration of licochalcone A (Figure 6), the weight of liver tissue decreased compared to high fat-induced fatty liver. Nevertheless, licochalcone A also reduced the expression of a transcription factor that helps in lipogenesis and synthesis of fatty acids.

Interestingly, it has also been observed that licochalcone A was able to activate the sirt-1/AMPK path to attenuate the fatty acid chain synthesis and to enhance the lipolysis and β-oxidation in hepatocytes (Table 6) (Liou et al., 2019). Another study was also conducted to evaluate the anti-atherosclerotic cardiovascular diseases where Hops (*Humulus lupulus* L.) was selected and isolated different metabolites, including xanthohumol. To delve into this context, HepG2 and Huh7 cells were used as *in vitro* models, and zebrafish were used as an *in vivo* model. As a result, xanthohumol reduced the expression of angiopoietin-like protein-3 (ANGPTL3) in both mRNA and protein level that function as inhibitor of lipoprotein lipase and thereby enhance the expression of lipoprotein lipase (Table 6) (Gao et al., 2024).

According to SAR research, prenylated chalcones have demonstrated superior pharmacokinetic and pharmacodynamic profiles compared to commercially available conventional medications. Prenylated chalcones including xanthohumol, xanthoangelol, isobavachalcone, 4-hydroxyderricin, and 2′,4′-dihydroxy-4-methoxy-3′-prenyldihydrochalcone had antiobesity potential and exhibited inhibition of AA (Arachidonic Acid), CETP, and DGAT. Calcium channel blockage requires the presence of a 1, 4-dihydropyridyl group at the 2- or 6-position of the A-ring, as well as a 4-OH substitution at B ring.3-, 4-, and 5-trimethoxy substitution is critical for inhibiting ACE, while the inhibition of PL requires the substitution of 3-OH or 4-OH at the B-ring and 2, 4-dihydroxy at the A-ring (Mahapatra and Bharti, 2016). Chalcones, such as 4-hydroxyderricin and xanthoangelol, exhibit significant cardiovascular benefits, including reducing blood pressure, cholesterol, and triglycerides and preventing atherosclerosis. Isoliquiritigenin shows potential in reducing myocardial infarct size and obesity. Licochalcone A, another chalcone, demonstrates antiobesity effects by promoting fat oxidation and thermogenesis while reducing lipogenesis. Chalcones like flavokawains and xanthohumol also show potential in protecting against nonalcoholic fatty liver disease and reducing atherosclerotic risk by regulating lipid metabolism pathways.

### 4.6 Antidiabetic roles of naturally occurring chalcones

Diabetes and related complications, such as diabetic neuropathy, retinopathy, and nephropathy, are main reasons of health suffering, morbidity, and mortality of diabetic patients. Globally, diabetes-related mortalities accounted for approximately 6.7 million deaths in 2021, and predicts that 537 million people globally were living with diabetes in 2021. If effective preventive measures are not taken, this figure is expected to rise to 643 million by 2030 (Federation ID, 2021). A variety of chalcone compounds exert antidiabetic activities (Rocha et al., 2020). Chalcones are among the molecules that have given promising results when examined for their antidiabetic actions (Mahapatra et al., 2015a). Antidiabetic property of chalcone derivatives is basically due to their inhibitory roles in glycolytic pathways and insulin-like activities (Rocha et al., 2020). Some of the important enzymes in this pathway are aldose reductase, α-amylase, α-glucosidase, etc. Apart from these enzymes the therapeutic target for the chalcones in control of diabetes also includes Sodium Glucose Cotransporter 2 (SGLT2), Glucose Transporter Type 4 (GLUT4), Peroxisome Proliferator-activated Receptor-gamma (PPAR-γ), Protein Tyrosine Phosphatase 1B (PTP1B), Dipeptidyl Peptidase 4 (DPP-4), and AMPK (Mahapatra et al., 2015a; Rocha et al., 2020).

Aldose reductase is one of the vital enzymes significantly related to the pathogenesis of diabetes mellitus. This enzyme catalyzes the reduction reaction of glucose and converts it into sorbitol, considered an aetiological factor for many diabetic complications. Therapeutic targeting of aldose reductase is therefore proposed as a noble strategy for treating diabetes mellitus (Suzen and Buyukbingol, 2003; Maccari and Ottanà, 2015). Aldose reductase inhibitors can be, therefore, the promising class of molecules for the treatment of diabetic patients. Similarly, α-glucosidase and α-amylase are the two other enzymes that make glucose available in the blood. Chalcones were found to inhibit not only the aldose reductase but also carbohydrate hydrolyzing enzymes, including α-glucosidase and α-amylase (Rocha et al., 2019) and also target many signaling pathways and proteins to mediate their antidiabetic effects (Adelusi et al., 2021).

In recent days, researchers are developing chalcone-based amines and hybrid chalcone molecules to increase the antidiabetic potential of existing chalcones (Mahapatra et al., 2017). From *Sophora flavescens* roots, Jung and associates isolated five prenylated flavanones and two prenylated chalcones, including β-hydroxy chalcone kuraridin, and investigated their repressive outcomes against the formation of advanced glycation endproducts (AGE), human recombinant aldose reductase (HRAR), and rat lens aldose reductase (RLAR) (Jung et al., 2010). All the isolated prenylated flavanones and chalcones act as strong inhibitor of RLAR and also inhibit AGE formation. When compared to the strong AR inhibitor epalrestat (IC_50_ = 0.28 µM), all tested prenylated metabolites, including kuraridin (IC_50_ = 0.27 µM) demonstrated considerable inhibitory effects (Table 7). SAR analysis on the isolated metabolites designated that the prenyl moiety at C-8 and the 3,4′-dihydroxyl moieties may, be responsible for the outcomes of diabetic complications, which include the suppression of RLAR, HRAR, and AGE production.

**TABLE 7:** Plant source, doses, and antidiabetic roles of different natural chalcones.

Name and structure of chalcone	Plant source	Smiles	Activity	Dose of drug	Target model	Actions	References
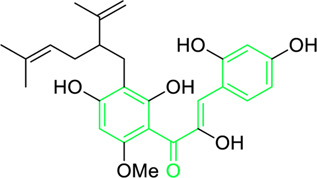 Kuraridin	*Sophora flavescens* Aiton (Family: *Fabaceae*)	O=C (/C(O) = C/C1 = CC = C(O)C=C1O)C2 = C(OC)C=C(O)C (CC(C(C) = C)C/C=C(C)/C) = C2O	Antidiabetic activity	IC_50_ = 0.27 µM	Lenses collected from Sprague–Dawley rat’s eyes	Suppression of RLAR, HRAR, and AGE production	Jung et al. (2010)
Isoliquiritigenin	*Glycyrrhiza glabra*.L. (Family: *Fabaceae*)	OC(C=C1O) = CC = C1C(/C=C/C2 = CC = C(O)C=C2) = O	Shows an antihyperglycemic effect	200 mg/kg	Male swiss albino mice	Changes in intracellular enzyme activity like glucosidase	Gaur et al. (2014)
4-hydroxyderricin (4-HD)	*Angelica keiskei*. (Miq.) Koidz. (Family: *Apiaceae*)	OC1 = C(C(/C=C/C2 = CC = C(O)C=C2) = O)C=CC(OC) = C1C/C=C(C)/C	Prevents progression of diabetes	Basal diets containing 0.15% of drug	KK-Ay/Ta mice (Genetically hyperglycemic)	Insulin-like action leads to increased uptake of glucose by cells	Enoki et al. (2007)
Xanthoangelol	*Angelica keiskei*. (Miq.) Koidz. (Family: *Apiaceae*)	OC1 = C(C/C=C(C)/CC/C=C(C)/C)C(O) = C(C(/C=C/C2 = CC = C(O)C=C2) = O)C=C1	Prevents progression of diabetes	Basal diets containing 0.15% of drug	KK-Ay/Ta mice (Genetically hyperglycemic)	Mimic insulin actions	Enoki et al. (2007)
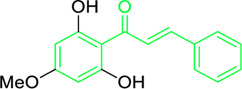 2′,6′-dihydroxy-4′-methoxychalcone	*Piper Claussenianum*. (Miq.) C.DC. (Family: *Piperaceae*)	OC1 = C(C(/C=C/C2 = CC = CC = C2) = O)C(O) = CC(OC) = C1	Lowers the blood glucose levels	2 mg/kg	Male Wistar rats (Streptozotocin induced diabetes)	Inhibition of enzymes like protein tyrosine phosphatase 1B, aldose reductase, and α-glucosidase Enhanced secretion of insulin	Sudo et al. (2015)
Xanthohumol	*Humulus lupulus* L. (Family: *Cannabaceae*)	O=C (/C=C/C1 = C(O)C=C(O)C=C1)C2 = C(OC)C=C(O)C(C/C=C(C)\C) = C2O	Alleviates hyperglycemia	IC_50_ = 8.8 µM	Caco-2 cells	Inhibition of *α*-glucosidase enzyme Xanthohumol directly binds to the α-glucosidase to change the molecular structure for inhibiting the enzymes	Liu et al. (2014b)
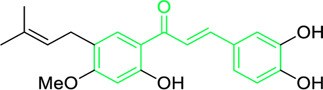 Broussochalcone A	*Broussonetia papyrifera*. (L.) L'Hér. ex Vent. (Family: Moraceae)	OC1 = CC(OC) = C(C/C=C(C)/C)C=C1C(/C=C/C2 = CC = C(O)C(O) = C2) = O	Antidiabetic	IC_50_ = 5.3 µM	*In-vitro* assay	Inhibition of α-glucosidase enzyme	Ryu et al. (2010a)
Isoliquiritigenin	*Glycyrrhiza uralensis*. Fisch. ex DC. (Family: *Fabaceae*)	OC(C=C1O) = CC = C1C(/C=C/C2 = CC = C(O)C=C2) = O	Antidiabetic activity by inhibiting hyperglycemia-induced inflammatory response	Up to 40 μM dose, no significant toxicity induced	H9c2 cells and male C57BL/6 mice	Attenuation of cardiac hypertrophy to protect the cardiac function Downregulation of mitogen-activated protein kinases and upregulation of Nrf2 signaling cascade	Gu et al. (2020)
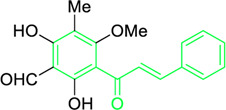 New chalcone	*Cleistocalyx operculatus* (Roxb.) Merr. and L.M.Perry (Family: *Myrtaceae*)	O=C (/C=C/C1 = CC = CC = C1)C2 = C(O)C(C=O) = C(O)C(C) = C2OC	Antidiabetic activity	IC50 value ranging from 0.9 ± 0.2 to 3.9 ± 0.7 μM		Inhibits the activity of protein tyrosine phosphatase 1B	Mai et al. (2024)

Isoliquiritigenin and its derivatives show very good antihyperglycemic properties against streptozotocin-nicotinamide-induced diabetic male Swiss albino mice (Table 7) (Gaur et al., 2014). SAR studies demonstrated that the occurrence of ether and ester moieties in isoliquiritigenin derivatives is vital for displaying the activity. Twenty types of chalcones are found in *Angelica keiski*, which is essential as a dietary supplement. Enoki and his group isolated two main chalcones 4-hydroxyderricin and xanthoangelol from *Angelica keiskei* ethanol extract (Enoki et al., 2007). Both the chalcones exhibited insulin-like actions by triggering PPAR-γ (Table 7). Furthermore, in genetically compromised KK-A^y^ mice, which acquire diabetes and exhibit hyperglycemia with aging due to resistance of insulin, 4-hydroxyderricin (0.15%) also reduced the progression of diabetes.

Another chalcone named 2′, 6′-dihydroxy-4′-methoxychalcone was described to prominently lesser the blood glucose levels in streptozotocin-induced diabetes rats model suppression of enzymes including protein tyrosine phosphatase 1B, α-glucosidase aldose reductase, and increased secretion of insulin (Sudo et al., 2015). After 12 days, the blood glucose levels of the rats administered 2′, 6′-dihydroxy-4′-methoxychalcone (2 mg/kg) dropped from 277.4 ± 7.7 mg/dL before treatment to 158.8 ± 9.2 mg/dL (Table 7).

Xanthohumol and papyriflavonol A alleviate hyperglycemia by inhibiting of α-glucosidase enzyme. The ability of xanthohumol (isolated from *Humulus lupulus* L.) to attach to α-glucosidase, decrease its hydrophobicity, and cause conformational changes in the enzyme structure to cause inhibition was studied by Liu and colleagues (Liu et al., 2014). The results demonstrated that xanthohumol inhibited α-glucosidase (IC_50_ = 8.8 μM) reversibly and noncompetitively (Table 7). Additionally, xanthohumol prevented glucose from being released from maltose on the apical side of the Caco-2 cell monolayer.

Ryu et al. isolated four chalcones and eight bioactive metabolites from the chloroform extract of *Broussonetia papyrifera* roots and examined their α-glucosidase inhibitory property (Ryu et al., 2010). ^1^H and ^13^C NMR, HMBC, HREIMS, and EIMS characterized the structure of the extracted metabolite. With an IC_50_ of 5.3 μM, the maximum effective chalcone-derived inhibitor was the prenylated chalcone broussochalcone A, which has a resorcinol moiety in the A ring and a catechol in the B ring (Table 7). From SAR studies it emerges that hydrophobic moieties around the aromatic core and a higher number of prenyl moieties enhance the effectiveness of the inhibitor. Furthermore, in kinetic studies, all the extracted chalcones displayed noncompetitive inhibition characteristics.

A natural chalcone, isoliquiritigenin, was evaluated for anti-inflammatory and antioxidant activity. To see these activities, streptozotocin-induced diabetic mice were used where isoliquiritigenin was administrated. Thus, high hyperglycemia was also induced using the H9c2 cells, which was generated from the embryonic rat heart. Consequently, isoliquiritigenin successfully inhibited apoptosis, fibrosis, and hypertrophy in H9c2 cells by lowering oxidative stress and the inflammatory response (Table 7). Additionally, it increased the Nrf2 signaling pathway and downregulated the MAPKs (Gu et al., 2020).

Recently, Mai and colleagues isolated four novel compounds (3 racemic chalcone-monoterpene hybrids and a novel chalcone) from the buds of *Cleistocalyx operculatus* and investigated the inhibitory effects on PTP1B. The isolated compounds’ structures were determined by analyzing NMR data and validated by computational techniques. The chalcone-myrcene compounds were found to be promising inhibitors by the *in vitro* PTP1B inhibitory experiment, and also isolated novel chalcone demonstrated good efficacy as well, with an IC_50_ of 3.9 ± 0.7 µM (Mai et al., 2024) (Table 7). PTP1B plays a significant role in insulin signal transduction, further regulating insulin receptor activity and its downstream signaling proteins well (Johnson et al., 2002).

Aldose reductase plays a vital role in diabetes, and inhibiting this enzyme, and others like α-amylase and α-glucosidase, is a promising strategy for treating diabetic complications. Chalcones have shown potential in inhibiting these enzymes and regulating various antidiabetic pathways. Chalcones’ structural modifications make them a prospective treatment candidate for diabetes control. The 2′-hydroxyl group is a crucial metabolite of natural chalcones, providing substantial activity through the formation of hydrogen bonds and maintaining the moiety’s structural stability. Chalcones with a 2′- and 4′-OH or with additional hydroxylation in ring B demonstrate strong antidiabetic properties (Rammohan et al., 2020). Furthermore, PTP1B inhibitory activity was significantly increased by adding two -OH groups to the A ring’s 2- and 4-positions. Moreover, the -OH group at 4 or 5 position of ring A and the -OCH_3_ group at 4 position of ring B in the chalcone were crucial for the activation of PPARγ(Mahapatra et al., 2015a). Chalcones like xanthohumol, 4-hydroxyderricin, and isoliquiritigenin exhibit strong inhibitory effects on diabetic markers and have shown positive results in reducing blood glucose levels, improving insulin sensitivity, and protecting against diabetes-related oxidative stress and inflammation.

### 4.7 Neuroprotective activities of naturally occurring chalcones

Neurodegenerative ailments like Alzheimer’s disease (Author Anonymous, 2024), and Parkinsons’s disease (PD) are very common among the aged person due to neuronal death. Global data as of 2020 indicated that 9.4 million people worldwide have Parkinson’s disease, with 930,000 of those cases occurring in the United States alone. Germany and Japan, probably in the hundreds of thousands. According to information from the WHO, approximately 329,000 fatalities worldwide occurred as a result of Parkinson’s disease till 2021 (https://ourworldindata.org/grapher/deaths-from-parkinsons-disease-ghe#sources-and-processing, n.d.). However, 6.9 million Americans are thought to be suffering from AD. By 2060, it is projected that this data might rise to 13.8 million. AD government data indicates that 119,399 deaths have been reported (2024 Alzheimer’s disease facts and figures, 2024). AD is a progressive neurologic syndrome that causes brain atrophy and leads to progressive deterioration of cognitive functions and memory loss. PD, in turn triggered due to loss of dopaminergic neurons in the substantia nigra. These settings are found to be contributed by neuroinflammation and oxidative stress in neuronal structures (Kim et al., 2012). Therefore, the agents that can check oxidative stress and neuroinflammation, in general, have neuroprotective activity and can give therapeutic benefits against neurodegenerative diseases. Many natural chalcones and their derivatives were reported to have neuroprotective action. The polar surface areas of chalcones are tiny, which helps them pass through the blood-brain barrier (BBB) and act on the central nervous system (CNS). This characteristic is mostly related to the two aromatic nuclei of the A and B rings’ hydrophobic nature (Mathew et al., 2015). The most often recommended course of treatment for AD is acetylcholinesterase inhibitors (AChEIs). It has been discovered that AChEIs increase attention span and slow the disease’s progression. Chalcones exhibit promising anti-neuroinflammatory action (suppression of iNOS or initiation of Nrf2 signaling) and are prospective enzyme inhibitors (MAO B, COMT, AChE), α-synuclein imaging probes, and antagonists of adenosine A1 and/or A2A receptors. Chalcones offer a powerful neuroprotective approach by regulating neurotrophic, inflammatory, and oxidative pathways.

*In vitro* and *in vivo* models of gliosis and neurodegeneration have been done by Kim et al. to assess licochalcone E’s capability to trigger the Nrf2/ARE path (Kim et al., 2012). Licochalcone E inhibited the inflammatory reactions that LPS caused in BV2 cells and shielded neuronal SH-SY5Y cells (Figure 6) from 6-OHDA cytotoxicity (Table 8). Licochalcone E stimulates the Nrf2-ARE pathway and upregulates NQO1 and HO-1 in downstream (Figure 6). Accompanying the *in vivo* 1-methyl-4-phenyl-1.2,3,6-tetrahydropyridine (MPTP) animal model, licochalcone E’s cytoprotective effect and upregulation of HO-1 and NQO1 were displayed. A specific HO-1 or NQO1 inhibitor, and siRNA-mediated Nrf2-silencing cells, were used to validate Nrf2’s role in licochalcone E’s cytoprotective and anti-inflammatory activities.

**TABLE 8:** Plant source, doses, and neuroprotective activity of different natural chalcones.

Name of chalcone	Plant source	Smiles	Activity	Dose of drug	Target	Mechanism of action	References
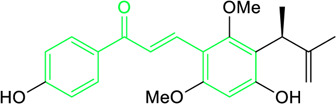 Licochalcone E	*Glycyrrhiza inflata* Batalin (Family: *Fabaceae*)	OC1 = CC = C(C(/C=C/C2 = C(OC)C=C(O)C ([C@@H](C)C(C) = C) = C2OC) = O)C=C1	It shows neuroprotective effect against dopaminergic neurodegeneration	10 mg/kg	Chemical induce neurodegenerative C57BL6 mice	Initiation of Nrf2-antioxidant response Up-regulations of the NQO1 and HO-1	Kim et al. (2012)
Butein	*Rhus vernciflua* Stokes (Family: *Anacardiaceae*)	OC1 = CC = C(C(/C=C/C2 = CC = C(O)C(O) = C2) = O)C(O) = C1	Inhibit neuroinflammation and production of NO. Memory enhancing effects	IC_50_ = 10.9 ± 2.3 µM	LPS induced BV2 cells (Mouse microglia cell line) Scopolamine induced memory-impaired Male ICR (Harlan Sprague–Dawley) mice	Suppression of COX-2 as well as iNOS expression Activation of cAMP responsive neurotrophic factor (BDNF) pathway	Cho et al. (2013)
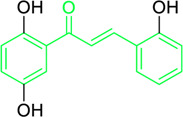 2.2′,5′-trihydroxychalcone (225THC)	Botanical drugs extracted chemical marketed by Indofine Chemical Company Inc.	O=C (/C=C/C1 = CC = CC = C1O)C2 = C(O)C=CC(O) = C2	Exerts anti-apoptotic and anti-inflammatory activity in primary rat neuronal cell culture Also inhibits LPS-mediated secretion of IL-6, and TNF-α	50 or 500 *μ*M	Primary Cultures of Microglia from Sprague Dawley rat pups	Not reported clearly	Jiwrajka et al. (2016)
Chalcones from Ashitaba (ChA)	*Angelica keiskei* (Miq.) Koidz. (Family: *Apiaceae*)		Neuroprotective effect	300 or 600 mg/kg	C57BL/6 mice	Attenuated the extent of demyelination in TNFα (corpus callosum and brain levels)	Rowhanirad and Taherianfard (2023)
Licochalcone A	Not reported	OC1 = C(C(C) (C)C=C)C=C (/C=C/C(C2 = CC = C(O)C=C2) = O)C(OC) = C1	Antioxidant and neuroprotective	IC_50_ = 42.28 ± 0.06 μM (inhibiting the butyrylcholinesterase) IC_50_ 23.41 ± 0.02 μM (inhibiting acetylcholinesterase)	Not reported	It showed potent antioxidant properties, and AChE/BChE inhibition activity	Budziak-Wieczorek et al. (2023)

The neuroprotective actions of natural chalcones are because of their capability to prevent the enzyme monoamine oxidases (MAOs), and cholinesterases, reverse neuroinflammation, and prevent neuronal apoptosis. It is a fact that elevated monoamine oxidase activity is related with the accumulation of ROS, depression, neurodegeneration, and hampered cognitive abilities (Naoi et al., 2012). Encoded by the MOA-B gene, this enzyme is known for its vital role in the catabolism of biogenic amines, including 2-phenylethylamine, benzylamine, and dopamine, in the CNS. Similarly, cholinesterases, that catalyze the breakdown of the neurotransmitter acetylcholine are involved in many neuronal diseases. The use of cholinesterase inhibitors remains one of the important approaches for the treatment of AD and many others (Larner, 2010). Therefore, MAOs and cholinesterases are therapeutic targets for treating many neuronal or brain health problems. Identifying chalcone compounds inactivating monoamine oxidase and cholinesterase can be helpful in developing pharmaceuticals for various neuronal disorders, such as PD and AD, managing stress, neuroinflammation, etc., (Cesura, 2007; Chimenti et al., 2009). Numerous natural chalcones and their derivatives have been described to have inhibitory action on MAO-A and -B, AChE, and butyrylcholinesterase (BChE) (Tanaka et al., 1987; HARAGUCHI et al., 2004; Kang et al., 2012; Robinson et al., 2013; Liu et al., 2014; Zhang et al., 2019).

**FIGURE 7:**
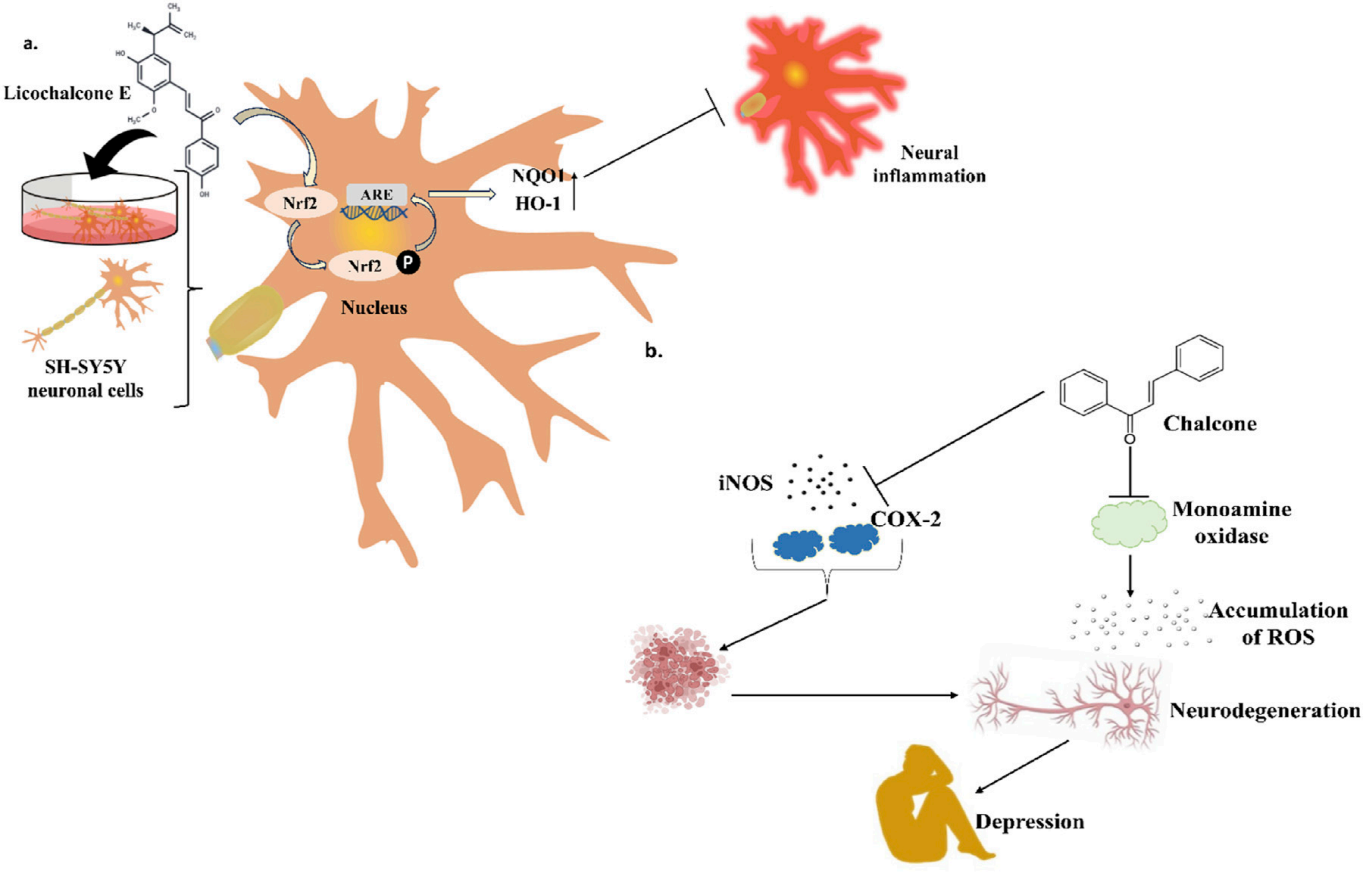
**(a)** Licochalcone E triggers the upregulated expression of NQO1 and HO-1 by regulating the Nrf2-ARE path to prevent neural inflammation. **(b)** Chalcones are associated with treating neurodegenerative diseases like depression by modulating the role of different signaling proteins, enzymes, and molecules, including NF-κB, STAT3, MMP-9, COX-2, and iNOS.

Chalcones and their derivatives also exert neuroprotective activity in *in vitro* neuronal cell culture and prevent inflammation and apoptosis by inhibiting iNOS and COX-2 expression (Figure 7) (Cho et al., 2013; Jiwrajka et al., 2016). Cho and colleagues isolated and characterized by spectroscopic methods chalcone butein along with flavonoid fisetin from ethyl acetate part of *Rhus verniciflua* bark and assessed their neuroprotective and anti-inflammatory activities (Cho et al., 2013). To delve into this study, it has been reported that a chalcone, 2,2′,5′-trihydroxychalcone isolated from the botanical drugs extraction showed anti-inflammatory and anti-apoptotic action in neural cells in 50 or 500 *μ*M concentration (Table 8). It also inhibits the secretion of TNF-α as well as IL-6 pro-inflammatory cytokines that are triggered by LPS (Jiwrajka et al., 2016). The authors attributed that reducing the expression of this gene caused in a less neurotoxic microglial phenotype, which could prove advantageous for certain neurodegenerative illnesses where abnormal microglial inflammatory reactivity is connected. In *in vivo* system, the application of chalcones prevents toxic chemical-induced neurodegeneration, upregulates the secretion of neurotrophic factors as well and enhances memory (Kim et al., 2012; Cho et al., 2013; Liu et al., 2013).

Chalcone butein exhibited strong neuroprotective properties towards neuroinflammation and neuronal death caused by glutamate and LPS. The strong inhibitory properties of butein *in vitro* have been attributed to the cytotoxicity of chalcone, which has an α, β-unsaturated double bond, a 4-OH group, and no C ring. One typical example of a disease related to the central nervous system is multiple sclerosis. Currently, cuprizone-induced multiple sclerosis in C57BL6 mice, a multiple sclerosis model, can be reduced by chalcones isolated from the *Ashitaba*. It was observed that these chalcones can diminish the expression of TNF-α in serum and the brain. Importantly, it improved the behavioral responses significantly (Table 8) (Rowhanirad and Taherianfard, 2023).

The plant-derived chalcone cardamonin is present in some plant species, including *Alpinia conchigera* and *Alpinia katsumadai*. In addition to reducing oxidative stress and regulating inflammatory responses, this plant-derived chalcone can regulate brain diseases. It's fascinating to note that it can alter the expression of NF-κB and STAT3. Furthermore, cardamonin can change the expression of certain enzymes, including MMP-9 and COX-2, as well as proteins associated with apoptosis, including Bcl-2 and cyclin D1 (Table 8). Significantly, the therapeutic aspect of neurodegeneration has also been documented to benefit from its modulatory effects on miRNA (Barber et al., 2023).

A recent study explored the role of natural chalcones including cardamonin, isobavachalcone, xanthohumol, 2′-hydroxy-4,4′,6′-trimethoxychalcone, and licochalcone A as a neurodegenerative disease protector. For the first time, these isolated natural chalcones were characterized based on a spectroscopic study to analyze the structural features with variable numbers and positions of -OH moieties in rings. To delve into the antioxidant and anti-neurodegenerative properties, 1,1- diphenyl-2-picrylhydrazyl was used as a free-radical scavenging reagent. Thus, licochalcone A inhibited butyrylcholinesterase (IC_50_ = 42.28 ± 0.06 μM) and acetylcholinesterase (IC_50_ = 23.41 ± 0.02 μM), demonstrating possible antioxidant action with a neuroprotective role (Table 8) (Budziak-Wieczorek et al., 2023). The actions of naturally occurring chalcones in terms of their neuroprotective ability have been listed in Table 8.

Chalcones, natural metabolites with neuroprotective properties, can inhibit enzymes like MAOs and cholinesterases, which are crucial in managing AD and PD. Studies have shown that chalcones activate protective pathways, reduce neuroinflammation, and prevent neuronal apoptosis. According to the SAR studies AChE inhibitory activity was increased upon the existence of electron-donating groups, whereas activity was decreased upon the existence of electron-withdrawing groups (Aslan et al., 2019). Furthermore, the presence of several functional moieties originating from -O and -N improved the overall inhibitory characteristics of AChE. Moreover, by regulating the expression of the cell death signal factor, chalcones with a prenyl moiety also showed neuroprotective properties.

Specific chalcones, such as licochalcone E, cardamonin, and butein, have demonstrated neuroprotective and anti-inflammatory activity, highlighting their significance as therapeutic agents for neurodegenerative disorders.

## 5 Marketed and clinically approved chalcones

**FIGURE 8:**
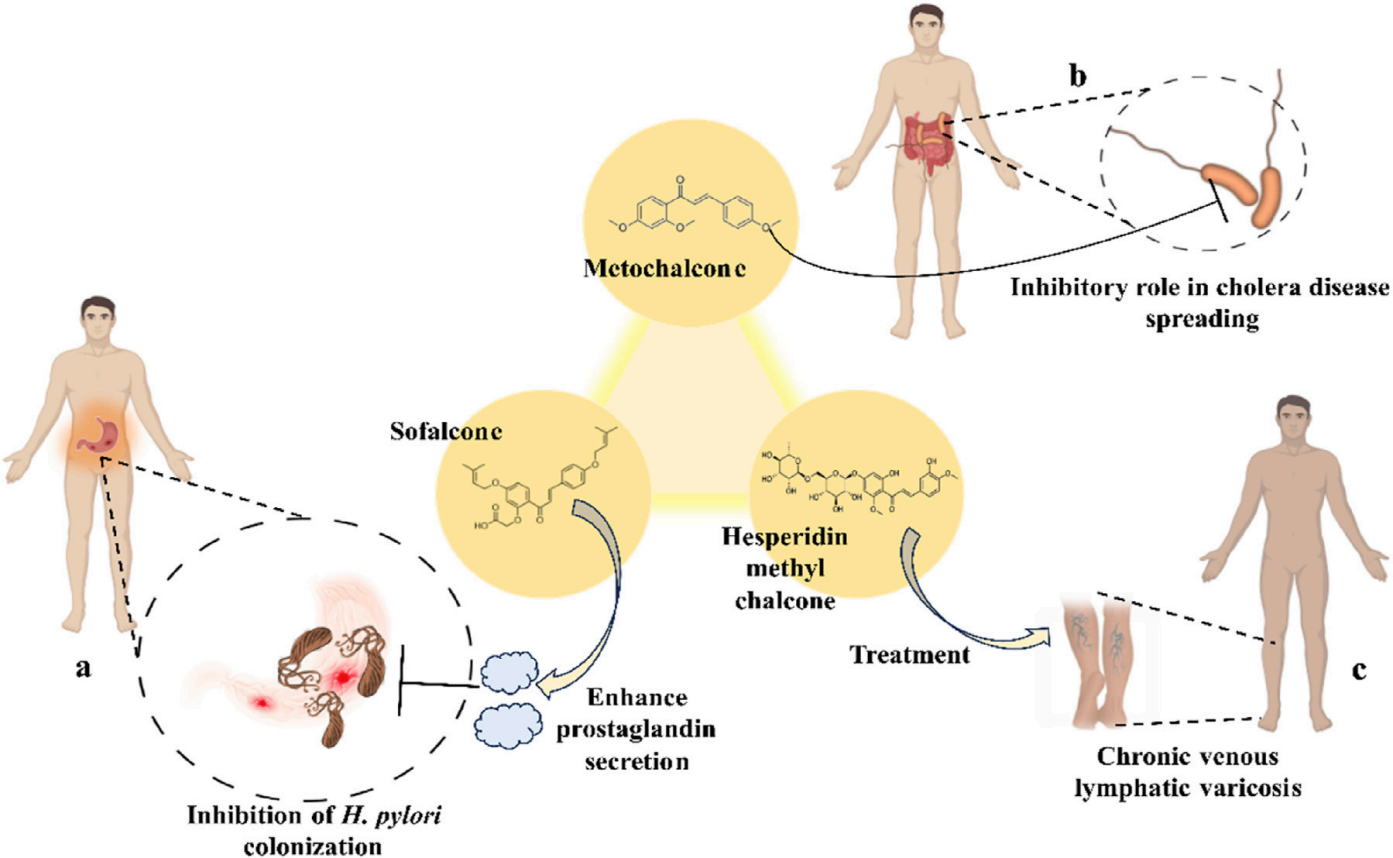
Role of marketed and clinically approved chalcones in different disease treatments. **(a)** Sofalcone can reduce the colonization of *H. pylori* by upregulating the expression of prostaglandin; **(b)** Metochalcone can also be a pharmacologically active reagent to treat cholera disease; **(c)** Hesperidin methyl chalcone has been applied clinically to treat chronic venous lymphatic varicosis.

Several chalcones, including sofalcone, metochalcone, and hesperidin methylchalcone, have been approved for clinical uses and usage in clinical settings (Cheng et al., 2020). To prevent *Helicobacter pylori* from advancing the condition, sofalcone (Figure 8) has been approved as an anti-ulcer drug that enhances prostaglandin in the mucosal area (Higuchi et al., 2010). Conversely, hesperidin methylchalcone (Figure 8) was employed in a clinical trial (Weindorf and Schultz-Ehrenburg, 1987; Gomes et al., 2017), to treat chronic venous lymphatic varicosis, and metochalcone (Figure 8) was approved to treat cholera infections (Sahun et al., 2012b). These chalcones are used in various clinical applications (Jandial DD et al., 2014; Gomes et al., 2017). They can also be altered by the addition of functional groups such as phenyl, halogens, hydroxyl, aryls, and carboxyl (Gomes et al., 2017) to enhance their target specificity when combined with other molecules in potential therapeutic applications. Furthermore, by hybridizing chalcone with other anti-cancer drugs, which helps to overcome the drug resistance of melanoma cells, chalcones are turning into a very important molecule for developing novel anti-cancer therapeutics.

## 6 Importance of chalcone as a traditional medicine over chemotherapy

Chalcone is an essential step in the flavonoid biosynthesis pathway, and plants containing chalcone have been utilized in traditional medicine since antiquity. The chemical structure of α,β-unsaturated ketones significantly influences pharmacological actions such as anti-cancer, antibacterial, immunosuppressive, and anti-inflammatory properties (Rudrapal et al., 2021). The privileged structure of a molecule is fundamental in medicinal chemistry, particularly in drug discovery. In this regard, chalcone is a typical simple scaffold as a natural form that can be utilized to synthesize a large number of derivatives. However, its conjugated structure with electron pulling and pushing functional groups on the benzene rings, can also be employed in imaging-based disease diagnosis (Zhuang et al., 2017c). It was also discovered that chalcone derivatives can be easily synthesized using various synthetic methods. These synthetic analogs have been demonstrated to have bioactivities to their natural counterparts, but with increased potency and lower toxicity (Jasim et al., 2021). On the other hand, synthetic compounds used for different medicinal purposes can have a variety of side effects, some of which are severe. As a result, chalcone-based conventional therapies may be a promising area of medicinal chemistry based on its unique structural feature, pharmacological activities, and less toxic characteristics.

## 7 Critical finding in chalcone-based drug designing

Chalcone is a highly effective plant-derived natural chemical due to its structural properties, which may be easily modified to produce a wide range of derivatives against various diseases such as bacterial infection, inflammatory diseases, cancer, neurological disorders, and so on. It was also shown that various plant-based chalcone derivatives can target cellular signaling pathways to modify metabolic activities, hence improving therapeutic efficacy. However, rigorous study has revealed that balancing the safety and efficacy of chalcone-based medicines is equally vital. In this connection, it was discovered that chalcone-based derivatives in plants can likewise produce the required effects without hurting the primary organs (Muller and Milton, 2012). However, this metabolite may also pose a hurdle because it has demonstrated distinct therapeutic windows in a number of *in vitro* and *in vivo* studies. Another crucial result that should be taken seriously is the contribution of multidrug resistance via chalcone derivatives in cancer cells when efflux pumps are overexpressed (Hba et al., 2023a). To investigate this context, it was first discovered that various possible chalcones have been employed as modulators of resistance to conventional medications by targeting P-glycoproteins (multidrug efflux transporters) (Bois et al., 1998; Parveen et al., 2014; Ngo et al., 2016), and multidrug resistance protein 1 (Nguyen et al., 2003; Lindamulage et al., 2017). However, these transporters contribute significantly to multidrug resistance in cancer cells by accumulating medicines (Xiao et al., 2021).

## 8 Limitations and problems raised in chalcone-based drug designing

In drug design, various constraints and issues that arise in the form of unwanted qualities such as local irritations, toxicity, short half-life, poor absorption, and, most critically, chemical instability and low water solubility. It has also been noted that some medications can be discovered in an inactive state that undergoes *in vivo* biotransformation through enzyme activity, facilitating drug accumulation at the site of action (Rautio et al., 2008; Jornada et al., 2015). In this regard, it was determined that some chalcone derivatives may work as good anti-inflammatory drugs by neutralizing CXCL12 (CXC motif chemokine ligand 12), preventing it from acting on CXCR4 and CXCR7 receptors. However, its decreased solubility in water, it cannot function adequately (Hachet-Haas et al., 2008). On the other hand, in drug synthesis, employing chalcone as a scaffold, green synthesis is becoming a significant feature in reducing drug toxicity. However, due to its low solubility in water, water cannot be used as a green solvent to synthesis chalcone derivatives, which is another limitation (Marotta et al., 2022).

## 9 Future perspective

Overall, chalcones’ complex role in treating various diseases provides an opportunity for more investigation into them during the drug discovery process. However, it has numerous obstacles, which have been addressed in the limitations and critical assessment of chalcone-based drug design. When it comes to their administration as possible medicinal agents, chalcones’ poor solubility and the extent of their dissolution in the gastrointestinal tract pose serious challenges (Hba et al., 2023b). These limitations can be changed with hydrophilic polymers, which improve the plant-derived compounds’ solubility, bioavailability, and pharmacokinetics in many therapeutic applications.

Additionally, to fully utilize chalcones’ medicinal potential and incorporate them into robust pharmaceutical formulations, their poor solubility, stability, and toxicity must be resolved. Nanoparticles (NPs) are a new delivery method for chalcones that researchers have recently begun using. The numerous advantages that NPs provide improve the solubility, effectiveness, and possible uses of chalcones in drug delivery, making them a fascinating and attractive field for pharmaceutical study. Future research should concentrate on enhancing chalcone solubility, figuring out the best therapeutic dosages, identifying resistance mechanisms, and investigating combination treatments that incorporate chalcones and chalcone-based nanoparticles. To realize the full therapeutic potential of chalcone-based pharmaceuticals and enhance human health outcomes, more investigation is needed into their mechanisms of action and optimization.

## 10 Conclusion

In conclusion, the various bioactive chalcones extracted from the different parts of plants display an extensive range of pharmacological properties, for example, anti-inflammatory, antiviral, anti-cancer, and antidiabetic properties. Researchers are becoming more interested in chalcones for developing pharmacological substances because of their superior bioavailability and high tolerance in the body. Numerous chalcones found in nature have demonstrated one or more pharmacological properties. Additionally, we provided an overview of the SAR studies and discussed the pharmacological potential of the various chalcones isolated from the plants. Chalcones are unique, versatile scaffolds that have undoubtedly demonstrated great promise in medicinal chemistry. Some chalcones have superior activity than conventional medications and may eventually be introduced to the market as novel drugs. These substances might act as lead compounds in the development of novel drugs. Licochalcones, for instance, has shown substantial potential in inhibiting the growth of protozoan species responsible for leishmaniasis and malaria and demonstrating anti-cancer and antimicrobial activities. Moreover, chalcones like isoliquiritigenin and xanthohumol have been identified as potent inhibitors of multiple signaling pathways related to cancer progression and inflammation, with notable effects on angiogenesis, apoptosis, and enzymatic activities related to tumor growth.

Plant-derived natural chalcone compounds exhibit significant potential for managing diabetes and its related complications and neurodegenerative diseases. Their antidiabetic effects are primarily attributed to their inhibitory roles in key enzymes and pathways related to glucose metabolism, such as aldose reductase, α-amylase, and α-glucosidase. Additionally, natural chalcones have shown promising neuroprotective activities by targeting enzymes like monoamine oxidases and cholinesterases, which are crucial for developing neurodegenerative disorders, for example, Alzheimer’s and Parkinson’s diseases.

This thorough analysis delves deeply into the medicinal chemistry of natural chalcones and their therapeutic potential. Chalcones have a variety of modes of action and show promise in pharmacological activities, as evidenced by the results of multiple investigations. This review underscores the therapeutic potential of naturally occurring chalcone compounds in developing novel therapeutics for treating various diseases, leveraging their capability to modulate key biological pathways. Although experimental studies have demonstrated numerous pharmacological activities of chalcones, additional comprehensive research investigations are necessary to address the toxicological and pharmacokinetic concerns, especially in preclinical and clinical studies.

## Glossary

FDA: US Food and Drug Administration
^**1**^ **H-NMR**: Proton Nuclear Magnetic Resonance
SAR: Structure-activity Relationship
NADPH: Nicotinamide Adenine Dinucleotide Phosphate
NaOH: Sodium hydroxide
KOH: Potassium hydroxide
NaOEt: Sodium Ethoxide
**PdCl**_**2**_: Palladium(II) Chloride
**Na**_**2**_ **CO**_**3**_: Sodium Carbonate
**CeCO**_**3**_: Cerium Cobalt
**Pd(OAc)**_**2**_: Palladium(II) Acetate
**Ph**_**3**_ **P**: Triphenylphosphine
**K**_**2**_ **CO**_**3**_: Potassium Carbonate
DMF: Dimethylformamide
CO: Carbon Monoxide
**PdCl**_**2**_ **(PPh3)**_**2**_: bis(triphenylphosphine)palladium(II) dichloride
THF: Tetrahydrofuran
**AlCl**_**3**_: Aluminium Chloride
**N**_**2**_: Nitrogen Gas
**Ba(OH)**_**2**_: Barium Hydroxide
**Ca(OH)**_**2**_: Calcium Hydroxide
**Sr(OH)**_**2**_: Strontium Hydroxide
CaO: Calcium Oxide
NaH: Sodium hydride
LiHMDS: Lithium Hexamethyldisilazide
LiOH: Lithium Hydroxide
HCl: Hydrogen Chloride
**BF**_**3**_ **-Et**_**2**_ **O**: Boron Trifluoride Diethyl Etherate
**SOCl**_**2**_: Thionyl chloride
*p*-TsOH: para-Toluenesulfonic Acid
**CH**_**3**_ **CN**: Acetonitrile
CuI: copper(I) iodide
DBU: 1,8-Diazabicyclo[5.4.0]undec-7-ene
DCM: Dichloromethane
**CHCl**_**3**_: Chloroform
^**13**^ **C NMR**: Carbon-13 Nuclear Magnetic Resonance
TNF-α: Tumor Necrosis Factor-α
IL-1β: Interleukin-1 β
IL-6: Interleukin-6
MIC: Minimum Inhibitory Concentration
HRMS: High-Resolution Mass Spectrometry
NorA: A multidrug efflux pump expressed in methicillin-resistant *Staphylococcus aureus* (MRSA)
MDR: Multidrug Resistance
bc1 complex: The cytochrome bc1 complex
Complex II: Complex II or succinate dehydrogenase
P. yoelii YM: A clone of rodent malaria parasite *Plasmodium yoelii*
FRD: Fumarate Reductase
BCG: *Bacillus* Calmette-Guerin
**IC**_**50**_: Half-maximal Inhibitory Concentration
BVDV: Bovine Viral Diarrhea Virus
HCV: Hepatitis C Virus
HSV: Herpes Simplex Virus
HIV: Human Immunodeficiency Viruses
RSV: Rous Sarcoma Virus
YFV: Yellow Fever Virus
PBMC: Peripheral Blood Mononuclear Cell
TI: Therapeutic Index
NA: Neuraminidase
H1N1: Type-1 hemagglutinin (H) protein and a type-1 neuraminidase (N) protein
WT: Wild Type
HMB: 2-hydroxy-3-methyl-3-butenyl alkyl
HSQC: Heteronuclear Single-Quantum Correlation Spectroscopy
HMBC: Heteronuclear Multiple Bond Correlation
COSY: Correlation Spectroscopy
ROESY: Rotating Frame Overhauser Effect Spectroscopy
NCDs: Noncommunicable Diseases
DNA: Deoxyribonucleic acid
Rb: Retinoblastoma
TRAIL: Tumor Necrosis Factor-related Apoptosis-inducing Ligand
HDACs: Histone Deacetylase Enzymes
NF-κB: Nuclear Factor Kappa B
EGFR: Epidermal Growth Factor Receptor
MMPs: Matrix Metalloproteinases
VEGF: Vascular Endothelial Growth Factor
ERK: Extracellular Signal-regulated Kinase
P13-K/Akt: Phosphoinositide-3-kinase–protein Kinase B
MAPKs: Mitogen-activated Protein Kinases
HUVECs: Human Umbilical Vein Endothelial Cells
HIF-1α: Hypoxia-inducible Factor-1α
TUNEL: Terminal Deoxynucleotidyl Transferase dUTP nick-end labeling
HCC: Hepatocellular Carcinoma
CCA: Cholangiocarcinoma
DRI Values: Dietary Reference Intake Values
ATM: Ataxia Telangiectasia Mutated
CRC: Colorectal Carcinoma
CDKs: Cyclin-dependent Kinases
NSCLC: Nonsmall Cell Lung Carcinoma
JNK: Jun N-terminal Kinase
c-IAP: Cellular Inhibitor of Apoptosis Protein
XIAP: X-linked Inhibitor of Apoptosis Protein
c-FLIPL: Cellular FLICE (FADD-like Il-1β-converting enzyme)-inhibitory Protein
RIP1: Receptor Interacting Protein-1
Glut1: Glucose Transporter 1
PDK1: Phosphoinositide-dependent Kinase 1
Ras: Rat sarcoma
Raf: Rapidly Accelerated Fibrosarcoma
MEK or MAPKK: Mitogen-activated Protein Kinase Kinase
PD-L1: Programmed Cell Death Ligand 1
GADD153: Growth Arrest and DNA Damage-inducible Gene 153
MEKC: Micellar Electrokinetic Chromatography
ROS: reactive Oxygen Species
p-mTOR: Phosphorylated Mammalian Target of Rapamycin
Akt/PKB: Protein kinase B, a serine/threonine protein kinase that regulates many cellular functions
P70S6K: p70 Ribosomal Protein S6 Kinase
PI3K: Phosphatidylinositol 3-kinase
BCL2: B-cell Lymphoma 2, a protein important for cell survival and apoptosis
NHEM: Normal Human Epidermal Melanocytes
NHDF: Normal Human Dermal Fibroblasts
PARP: Poly (ADP-ribose) Polymerase
DMC: 2′,4′-Dihydroxy-6′-methoxy-3′,5′-dimethylchalcone
5-FU: 5-fluorouracil
p-GSK3β: Phospho-glycogen Synthase Kinase 3 β
CH2Cl2: Dichloromethane
HTMC: 2′-hydroxy-2,3,4′,6′-tetramethoxychalcone
EAC: Ehrlich Ascites Carcinoma
TNBC: Triple-negative Breast Cancer
DDC: 2′,4-dihydroxy-4′,6′-dimethoxy-chalcone
FOXO3a: Forkhead Box Class O 3a
PUMA: p53 Upregulated Modulator of Apoptosis
Bax: Bcl-2 Associated X Protein
LC3: Microtubule-associated Protein 1A/1 B-light Chain 3
Wnt: Wingless and Int-1
Nrf2: Nuclear Factor Erythroid 2-related Factor 2
DPPH: 2,2-diphenyl-1-picrylhydrazyl
FT-IR: Fourier Transform Infrared Spectroscopy
DEPT: Distortionless Enhancement by Polarization Transfer
HMQC: Heteronuclear Multiple-Quantum Coherence
MALDI-TOF MS: Matrix-assisted Laser Desorption Ionization time-of-flight Mass Spectrometry
PGE2: Prostaglandin E2
NO: Nitric Oxide
FABMS: Fast Atom Bombardment Mass Spectroscopy
HRFABMS: High-resolution Fast Atom Bombardment Mass Spectroscopy
iNOS: Inducible Nitric Oxide Synthase
COX: Cyclooxygenase
LPS: Lipopolysaccharide
GSH: Glutathione
HO-1: Heme Oxygenase-1
NQO1: NAD(P)H Quinone Oxidoreductase 1
ICAM-1: Intercellular Adhesion Molecule-1
VCAM-1: Vascular Cell Adhesion Molecule-1
MS: Mass Spectrometry
Nrf2-ARE: Nrf2-antioxidant Response Element
FRAP: Ferric Reducing Antioxidant Power
ABTS: 2,2′-azino-bis (3-ethylbenzothiazoline-6-sulfonic acid
NGAL: Neutrophil Gelatinase-associated Lipocalin
IFN: Interferon
Keap1: Kelch-like ECH-associated protein 1
CFA: Complete Freund’s Adjuvant
CCK: Cholecystokinin
TMBC: 2,4,2′,4′-Tetrahydroxy-3-(3-methyl-2-butenyl)-chalcone
EIMS: Electron Ionization Mass Spectroscopy
HREIMS: High Resolution Electron Ionization Mass Spectroscopy
CYP1A: Cytochrome P450 1A
TrxR: Thioredoxin Reductase
QR: Quinone Reductase
HR-ESI-MS: High-resolution Electrospray Ionization Mass Spectrometry
GPx: Glutathione Peroxidase
PSTK: Phosphoseryl-tRNA Kinase
CVDs: Cardiovascular Diseases
SNPs: Single Nucleotide Polymorphisms
CRP: c - reactive protein
TGF-β: Transforming Growth Factor- β
LPL: Lipoprotein Lipase
PL: Pancreatic Lipase
ACE: Angiotensin-converting Enzyme
ACAT: Acyl-coenzyme A cholesterol acyltransferase
DGAT: Diacylglycerol Acyltransferase
CETP: Cholesteryl Ester Transfer Protein
TXA2: Thromboxane A2
TXB2: Thromboxane B2
LDL: Low-density Lipoprotein
SHRSP: Stroke-prone Spontaneously Hypertensive Rats
VLDL: Very-low-density Lipoprotein
LDL-R: LDL-receptor
PPAR-α: Peroxisome Proliferator Activated Receptor-α
ACO: Acyl-coenzyme A Oxidase
ACS: Acyl-CoA Synthetase
LDH: Lactate Dehydrogenase
CPK: Creatinine Phosphokinase
JAK/STAT: Janus Kinase/Signal Transducer and Activator of Transcription
MT: Metallothionein
HFD: High-fat Diet
UCP-1: Uncoupling Protein 1
ANGPTL3: Angiopoietin-like Protein-3
AA: Arachidonic Acid
SGLT2: Sodium Glucose Cotransporter 2
PPAR-γ: Peroxisome Proliferator-activated Receptor-gamma
PTP1B: Protein Tyrosine Phosphatase 1B
DPP-4: Dipeptidyl Peptidase 4
AMPK: Adenosine Monophosphate (AMP)-activated Protein Kinase
AGE: Advanced Glycation End-products
HRAR: Human Recombinant Aldose Reductase
RLAR: Rat Lens Aldose Reductase
AD: Alzheimer’s Disease
PD: Parkinsons’s Disease
BBB: Blood-brain Barrier
CNS: Central Nervous System
AChE: Acetylcholinesterase
AChEIs: Acetylcholinesterase Inhibitors
MPTP: 1-methyl-4-phenyl-1.2,3,6-tetrahydropyridine
MAOs: Monoamine Oxidases
BChE: Butyrylcholinesterase
